# Impact of Azobenzene Side Chains on the Ultraviolet–Visible and Fluorescence Properties of Coumarin Derivatives

**DOI:** 10.1021/acsomega.5c07385

**Published:** 2025-10-16

**Authors:** Yasemin Akdis, Akin Akdag

**Affiliations:** Department of Chemistry, Middle East Technical University, Ankara 06800, Türkiye

## Abstract

The effect of photoswitchable compounds on the light-emitting properties of nanoparticles has been drawing increasing attention. To investigate the effect of photoswitchable azobenzene units on the photophysical properties of coumarin, a biologically relevant fluorophore, photoresponsive azobenzene-coumarin derivatives were synthesized and characterized. The investigation of the effect of azobenzene isomerization on the ultraviolet (UV)–visible absorption and fluorescence properties of coumarin was explored. The azobenzene unit, attached to the coumarin chromophore at different positions via 3 and 8 carbon linkers, exhibited significant *trans* *–cis* isomerization upon UV irradiation at 365 nm, which affected both the absorption and fluorescence spectra of the coumarin part. The study demonstrated that the *trans*-to-*cis* transformation of the azobenzene moiety influences fluorescence intensity, with an increase observed in 7-hydroxycoumarin-derived compounds. However, for derivatives of 7-amino coumarin, the fluorescence intensity decreased. Density functional theory (DFT) and time-dependent DFT calculations suggested that the observed fluorescence changes are unrelated to the electronic coupling of the azobenzene and coumarin units. The increase in fluorescence is rather related to the absorption of excitation photons by *trans* azobenzene, and the decrease in fluorescence is due to the emitted photons absorbed by the azobenzene’s n*–*π* region.

## Graphical abstract

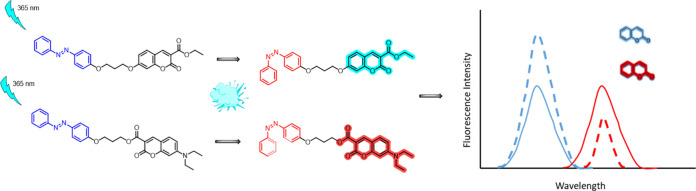

## Introduction

Photoresponsive chromophores have attracted considerable attention in molecular design due to their ability to modulate photophysical and photochemical properties in response to light. Azobenzene, a well-known photoisomerizable moiety, is a compound in which two aromatic moieties are linked via *–*N=N*–* bonding (Figure 1). This chromophore has been studied intensively for its photoresponsive character^1–4^ due to its reversible *trans* *–* *cis* isomerization, making it a versatile tool in supramolecular chemistry, materials science, and bioresponsive systems. The azo-compounds are thermodynamically more stable in their *trans* configuration than their *cis* configuration.^5,6^ As the *trans* *–* *cis* configuration change is usually attained by light, the *cis* configuration is converted back to *trans* thermally or photolytically.^7–9^ This configuration change has been exploited for studying the molecular motion.^8–15^

**Figure 1:**
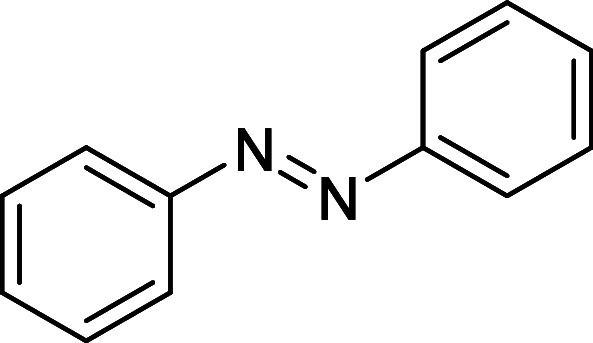
Azobenzene.

Exposure of *trans*-azobenzene to light between 320 and 380 nm is known to yield *trans* *–* *cis* isomerization, whereas exposure of the *cis* isomer between 400 and 450 nm has been observed to promote *cis* *–* *trans* isomerization (Figure 2). By irradiation with light of a specific wavelength, it is possible to control the configuration of each of the *trans* *–* *cis* photostable states.^8^

**Figure 2:**
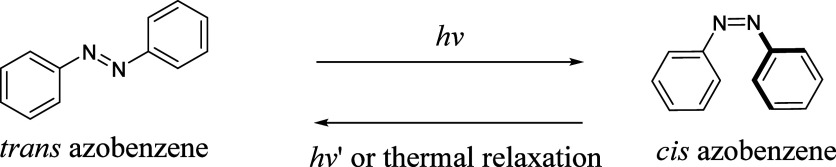
*Trans* *–* *cis* isomerization of the azobenzene.

Studies have demonstrated that the isomerization of azobenzene can influence nearby fluorophores through either conformational changes or electronic communications.^16^ In another study, azobenzene derivatives coupled with fluorophores such as cyanine dyes displayed near-infrared (NIR) fluorescence control through supramolecular encapsulation and isomerization, indicating potential in advanced imaging applications.^17^ The promise of azobenzene in the design of light-regulated fluorescence materials is further demonstrated by azobenzene-functionalized conjugated polymers, in which the fluorescence is modified as a result of changes in polymer conformation upon isomerization.^18^ Similarly, azobenzene-containing methacrylate polymers have been investigated for their dipole alignment and optical responses, indicating possible uses in optoelectronics.^19^ Furthermore, hybrid systems comprising porphyrins and azobenzene have shown adjustable photophysical characteristics, with photoisomerization influencing both emission and absorption patterns.^20^ When combined, these investigations demonstrate that azobenzene functions as a modulator of photophysical activity, specifically fluorescence, in addition to being a photoswitch.

Coumarin, on the other hand, is a well-known fluorophore in chemical sensing and bioimaging due to its inherent fluorescence, high quantum yields, and sensitivity to changes in the environment (Figure 3).^21^

**Figure 3:**
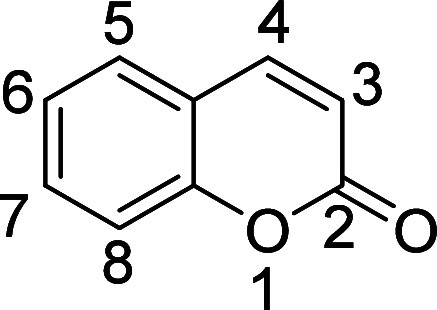
Structure and numbering of the coumarin.

Coumarin is a highly versatile chromophore that has been utilized to detect many species through its fluorescence properties.^21–23^ Furthermore, the UV–vis absorption profile of the coumarin unit can be tuned by installing different substituents on it.^24–26^ With its easy accessibility and being a good sensorial material, these qualities make coumarin derivatives useful for various applications.^21–28^ Studies using coumarin in luminescent probes and chromophoric materials have demonstrated that coumarin units can be successfully integrated into supramolecular structures and dye systems to investigate environment-sensitive fluorescence.^29^

Additionally, coumarin and/or azobenzene-containing systems, like dye–polymer systems or conjugated conducting polymers, show improved photostability and emission control, especially when exposed to different polarities or pH levels.^30,31^ Further demonstrating their versatility in multifunctional materials, the azobenzene moieties have also been coupled to electroactive platforms, where electronic influences have modified their fluorescence response.^32,33^

Although azobenzene and coumarin have been extensively studied independently, there are still only a few investigations that combine both chromophores in a single system. Several investigations have incorporated coumarin and azobenzene units into conjugated or polymeric structures, frequently emphasizing electrochemical or structural characteristics while just passingly discussing fluorescence activity.^16,20,33^ Specifically, the modulation of coumarin fluorescence by azobenzene photoisomerization within a covalently coupled molecular system has remained unexplored. Moreover, azobenzene, being a nonfluorescent modulator, and coumarin, being a responsive emitter, in a covalently bonded molecular system, have not been studied.

On the contrary to the molecular systems, nanoparticles were decorated with photoswitchable organic compounds to modulate their fluorescence properties.^34^ Toward this goal, gold nanoparticles were covered with azobenzene units.^35^ This study showed that when nanoparticles covered with azobenzene in the *trans* form were excited, the NIR fluorescence of the gold nanoparticles increased. On the other hand, the *cis* form decreased the NIR fluorescence. The enhancement in fluorescence for the *trans* was attributed to energy transfer from azobenzene to the nanoparticle. In another study, silicon nanoparticles were covalently attached to azobenzene.^36^ The fluorescence properties of the silicon nanoparticles did not change upon *E* to *Z* isomerization of the pendant azobenzene units.

We have initiated a study of coumarin, focusing on its capacity to detect environmental changes, specifically conformation and solvation.^37,38^ A natural legitimate question that emerged from this research is whether the *trans* *–cis* isomerization of the azobenzene side chain influences the UV–vis and fluorescence properties of the coumarin unit inspired by nanoparticle studies. In this regard, we synthesized new coumarin derivatives that incorporate azobenzene as a side chain and conducted a series of experiments to explore the properties of these compounds.

In this study, we aimed to address this untapped area of research by examining a unique chemical structure in which azobenzene and coumarin units are covalently linked and azobenzene isomerization directly affects coumarin fluorescence.

## Results and Discussion

The backbone of all synthesized compounds, 4-hydroxyazobenzene (**1**), was produced in high yield and analyzed using ^1^H NMR and IR spectroscopy in this investigation. Compounds **1a** and **1b** were produced by synthesizing two derivatives with hydroxyl-terminated alkyl chains using 3-bromo-1-propanol and 8-chloro-1-octanol to facilitate conjugation with coumarin. The purpose of these flexible linkers was to evaluate the impact of the spacer length on photophysical characteristics. Compounds **2a** and **2b** were created by tosylating the hydroxyl groups of **1a** and **1b** to enable nucleophilic substitution. In the meantime, the coumarin core, ethyl 7-hydroxycoumarin-3-carboxylate (**C1**), was created by Knoevenagel condensation. **FC1** and **FC2** were produced by coupling the tosylated azo derivatives to **C1**’s 7-position.

To identify the impact of positional variation, the 7-hydroxy group of compound **C1** was capped with butyl, and the 3-carboxylic ester was hydrolyzed to carboxylic acid (**C1a**), allowing EDC·HCl-mediated coupling with aliphatic azo-alcohols (**1a** and **1b**) to afford **FC3** and **FC4**. In order to examine electron-donating effects, ethyl 7-(diethylamino)­coumarin-3-carboxylate (**C2**) was synthesized and hydrolyzed; its derivatives **FC5** and **FC6** were obtained by coupling with compounds **1a** and **1b**. The characterization of each compound was conducted by using a combination of nuclear magnetic resonance (NMR), infrared (IR) spectroscopy, and high-resolution mass spectrometry (HRMS).

### Synthesis

The 4-hydroxyazobenzene moiety, **1**, which is present in the backbone of all target compounds, was obtained in 90% yield from the reaction of the corresponding diazonium salt obtained from aniline and phenol. It was characterized by ^1^H NMR and IR spectroscopy, and is consistent with the literature.^39^ As seen in Scheme 1, the compound **1** was further alkylated to get compounds **1a** and **1b**. These compounds were tosylated to form compounds **2a** and **2b**. These compounds (**1a**, **1b**, **2a**, and **2b**) will be used to obtain target compounds.

**Scheme 1:**
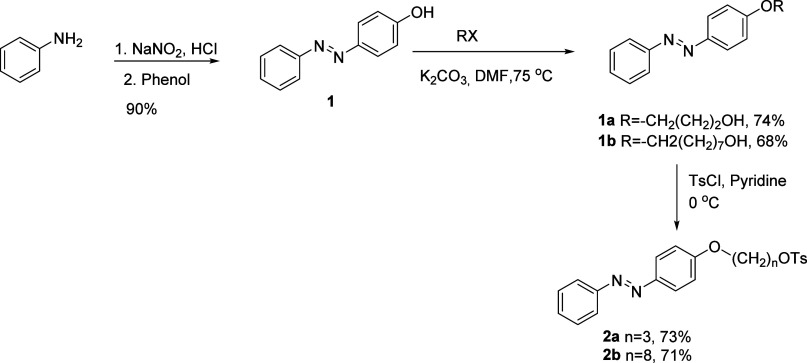
Synthesis of the Azobenzene Backbone

The synthesis of ethyl 7-hydroxycoumarin-3-carboxylate (**C1**) was accomplished through a Knoevenagel condensation, in which 2,4-dihydroxybenzaldehyde and diethyl malonate were reacted in the presence of piperidine as a base and acetic acid as a catalyst, under reflux conditions in ethanol.^40^ The same procedure was applied to synthesize **C2** as depicted in Scheme 2. The compound **C1** was butylated and subjected to saponification to get compound **C1a**.^41^ These compounds are used as reporters for our fluorescence studies.

**Scheme 2:**
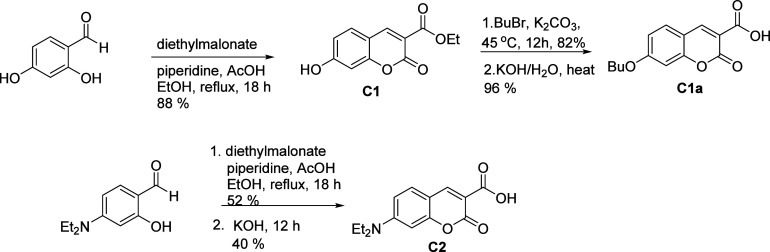
Synthesis of the Coumarin Derivatives

Photoresponsive azobenzene side chains were introduced to the 7-position of the coumarin unit by treating **2a**/**2b** with **C1**. This reaction successfully furnished compounds **FC1** and **FC2** (Scheme 3). To attach the azobenzene unit to the 3-position of coumarin units **C2** and **C1a**, EDC couplings were facilitated to bond **C2** and **C1a** with **1a** and **1b**. These reactions yielded **FC3**, **FC4**, **FC5**, and **FC6** as shown in Scheme 3.

**Scheme 3:**
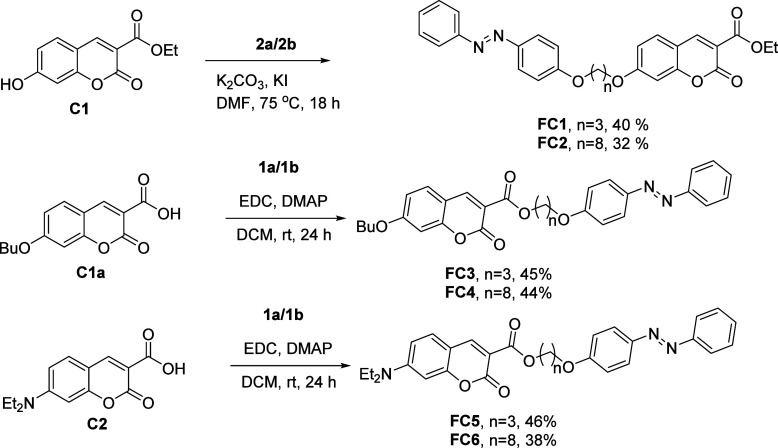
Synthesis of Final Compounds

### UV–Vis and Fluorescence Studies

UV–vis spectroscopy was used to probe the *trans*–*cis* transformation and vice versa. Since the *trans*-azobenzene derivatives have extended conjugation, π–π* transitions absorb light in longer wavelengths than their *cis* form.^3,8,42^ In order to be certain of the conversion of our synthesized aliphatic azo-alcohol, which is an azobenzene derivative, we performed a proof and control experiment with substance **1a** as shown in Figure 4. After measuring the sample prepared in DCM without exposure to light, it was exposed to 365 nm UV light for 10 min and then measured again. Immediately afterward, without intervening time, the same sample was immediately re-exposed to UV light for another 10 min and then measured again. The time expressions given throughout the paper are cumulative.

**Figure 4:**
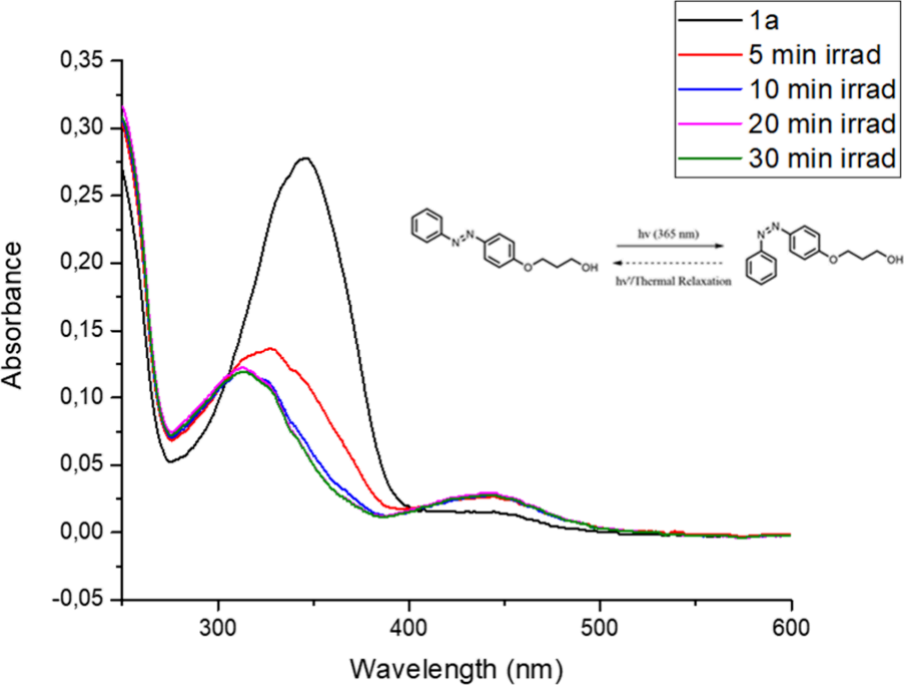
UV–vis absorption spectra of (*E*)-3-(4-(phenyldiazenyl)­phenoxy)­propan-1-ol (**1a**) in DCM and after its exposure to light at 365 nm for the corresponding time (10^–5^ M).

As seen for (*E*)-3-(4-(phenyldiazenyl)­phenoxy)­propan-1-ol (**1a**), the absorption λ_max_ = 320 nm decreases while compound **1a** (in *trans* configuration) is irradiated at 365 nm; that is, compound **1a** in the *trans* form is transformed into the *cis* form. The irradiating light source spectra overlap with aliphatic azo-alcohol **1a** in a small region; therefore, the rapid conversion from the *trans* to *cis* form is not observed. That is why even after 20 min of irradiation, *trans* azobenzene was present in the solution as it can be inferred from Figure 4.

Before the final compounds were studied, the UV–vis spectra of **1a** and **C1a** were also measured to determine the absorption profile of each chromophore. As seen in Figure 5, there is significant overlap between the absorptions of **1a** and **C1a**. Also, it should be noted that irradiation of the 7-butoxy coumarin **C1a** unit with a 365 nm wavelength did not affect the UV–vis spectrum of the compound after the irradiation.

**Figure 5:**
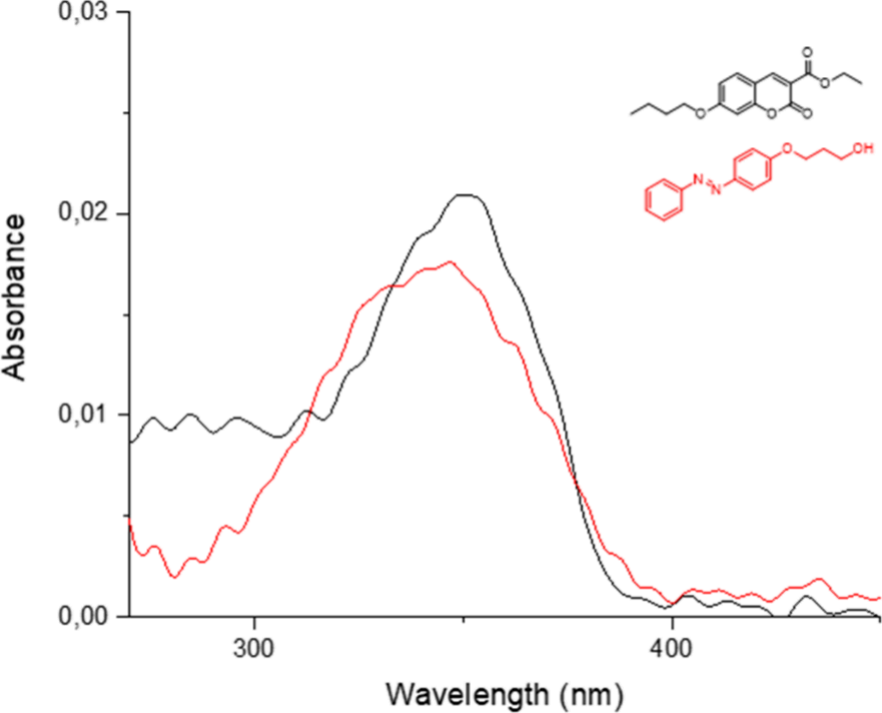
UV–vis spectra of compound **1a** and **ethyl**-**C1a** in DCM (10^–6^ M).

With these results in hand, we started our studies with **FC1**, which has an azobenzene side chain connected to the seventh position of the coumarin unit. The compound **FC1** was studied with the UV–vis spectrum showing λ_max_ = 348, with a shoulder at around 315 nm. After irradiation with 365 nm for 10, 20, and 30 min, the absorption decreased. Expectedly, this shows that the *trans* azobenzene unit transformed into the *cis* azobenzene unit (Figure 6). To investigate if coumarin’s fluorescence properties are changing during *trans*–*cis* isomerization, the emission and excitation spectra of the **FC1** with exposure to the light (λ = 365 nm) were recorded. Although λ_max_ of excitation was similar to that in the UV–vis spectra, as the compound was exposed to light, the excitation intensity increased. As to fluorescence spectra, a similar trend to excitation spectra was observed (Figure 6). Surprisingly, since the solution concentration did not change, this observation was unexpected.

**Figure 6:**
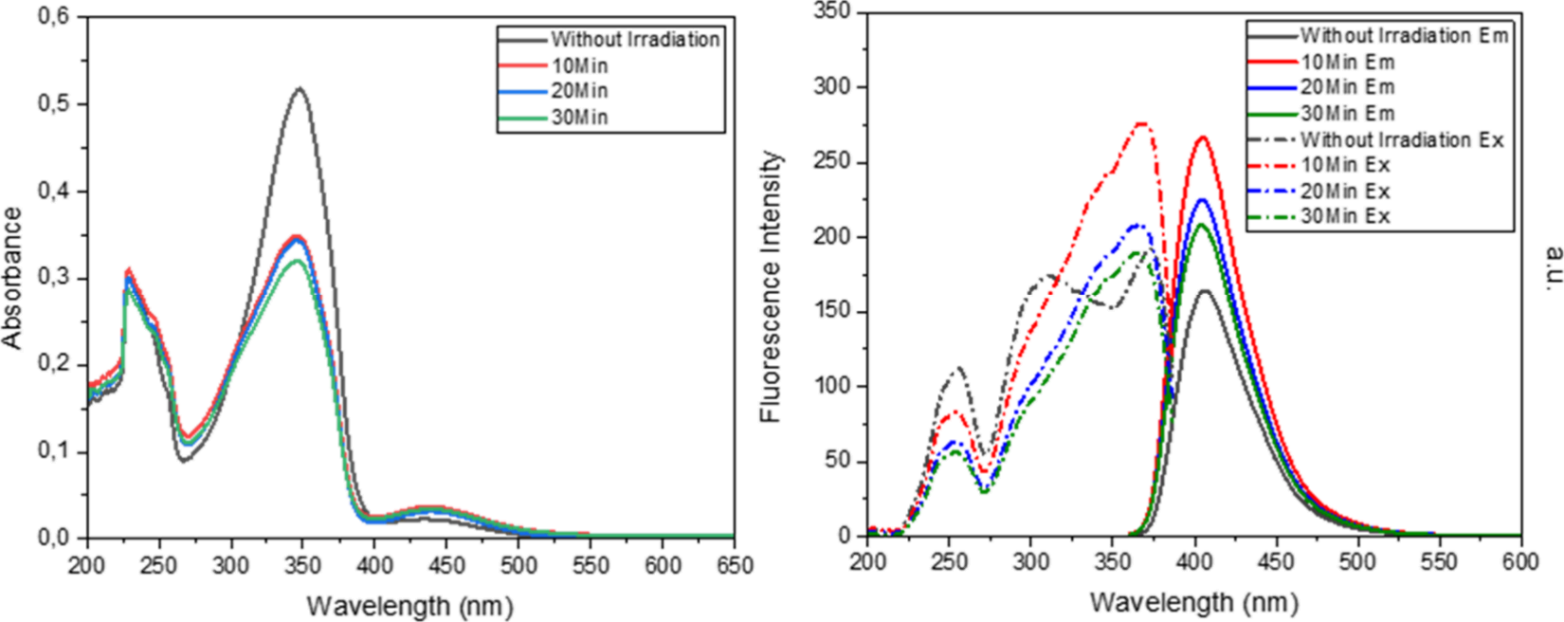
UV–vis and fluorescence spectra of **FC1** in DCM (10^–5^ M) before and after irradiation.

With this surprising observation in hand, to conclude if this observed phenomenon occurred due to the charge transfer, we ran the same experiments within the different solvents, i.e., THF, MeCN, and MeOH. Charge transfer is known to be affected by solvent polarity. The same phenomena as those in DCM were observed for these different solvents (Supporting Information). This excludes the possibility of photoinduced charge transfer affecting the fluorescence spectrum to some extent. If photoinduced charge transfer is a factor in these observations, then the distance between azobenzene and coumarin units should also be affected.

To test this approach, a derivative called **FC2** was constructed, in which an 8-carbon linker separated the azobenzene and coumarin units. The purpose of the longer chain was to test whether the fluorescence behavior is changed by the greater separation between the coumarin core and the photoresponsive azobenzene unit. Through this alteration, we were able to investigate whether the azobenzene group’s proximity promotes or inhibits charge transfer processes or any conformational changes that affect the molecule’s photophysical characteristics. We compared the optical responses of **FC1** and **FC2** to acquire a better understanding of how the relative position of the azobenzene side chain influences the coumarin fluorescence. For this purpose, we applied the same procedure, in which the 365 nm light irradiation was performed for 10, 20, and 30 min, and UV–vis and fluorescence studies operated in DCM (Figure 7). Analyzing the excitation and emission spectra of **FC2** revealed a rise in fluorescence intensity upon azobenzene isomerization, which is consistent with the trend reported in **FC1**, the 3-carbon-linked analog. To check the solvent dependency, we also performed these analyses again in THF, MeCN, and MeOH, and the results were consistent with those in DCM (Supporting Information).

**Figure 7:**
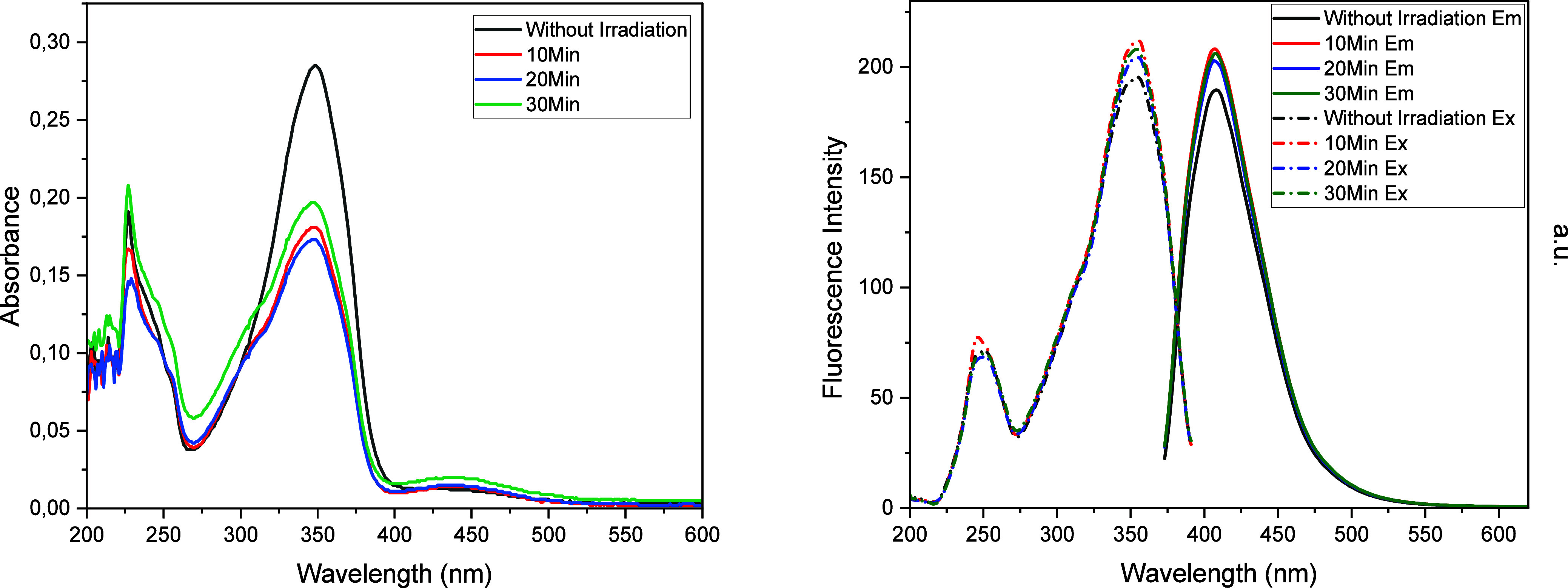
UV–vis and fluorescence spectra of **FC2** in DCM (10^–5^ M) before and after irradiation.

In order to further explore the positional effects of substituents on fluorescence enhancement, two new derivatives were synthesized: **FC3** and **FC4**. In these compounds, the seventh position of the coumarin core was capped with a butyl group, effectively removing any potential interaction at this position. This design serves to isolate the role of the azobenzene-coumarin interaction at a singular position, thereby facilitating a more comprehensive understanding of its influence. This approach enabled us to concentrate specifically on the impact of azobenzene side chains attached to the 3-position via linkers of varying lengths (3-carbon and 8-carbon aliphatic azo-alcohols). With this, **FC3** and **FC4** dissolved in DCM and UV–vis (λ_max_ = 350 nm for both), and a fluorescence study was performed in the same manner as that mentioned above. Results are shown in Figure 8, and the pattern was observed as it was observed for **FC1** and **FC2**. The results demonstrated a consistent enhancement in fluorescence intensity upon the *trans*-to-*cis* isomerization of the azobenzene unit across other solvents: THF, MeCN, and MeOH, each with varying polarity (Supporting Information).

**Figure 8:**
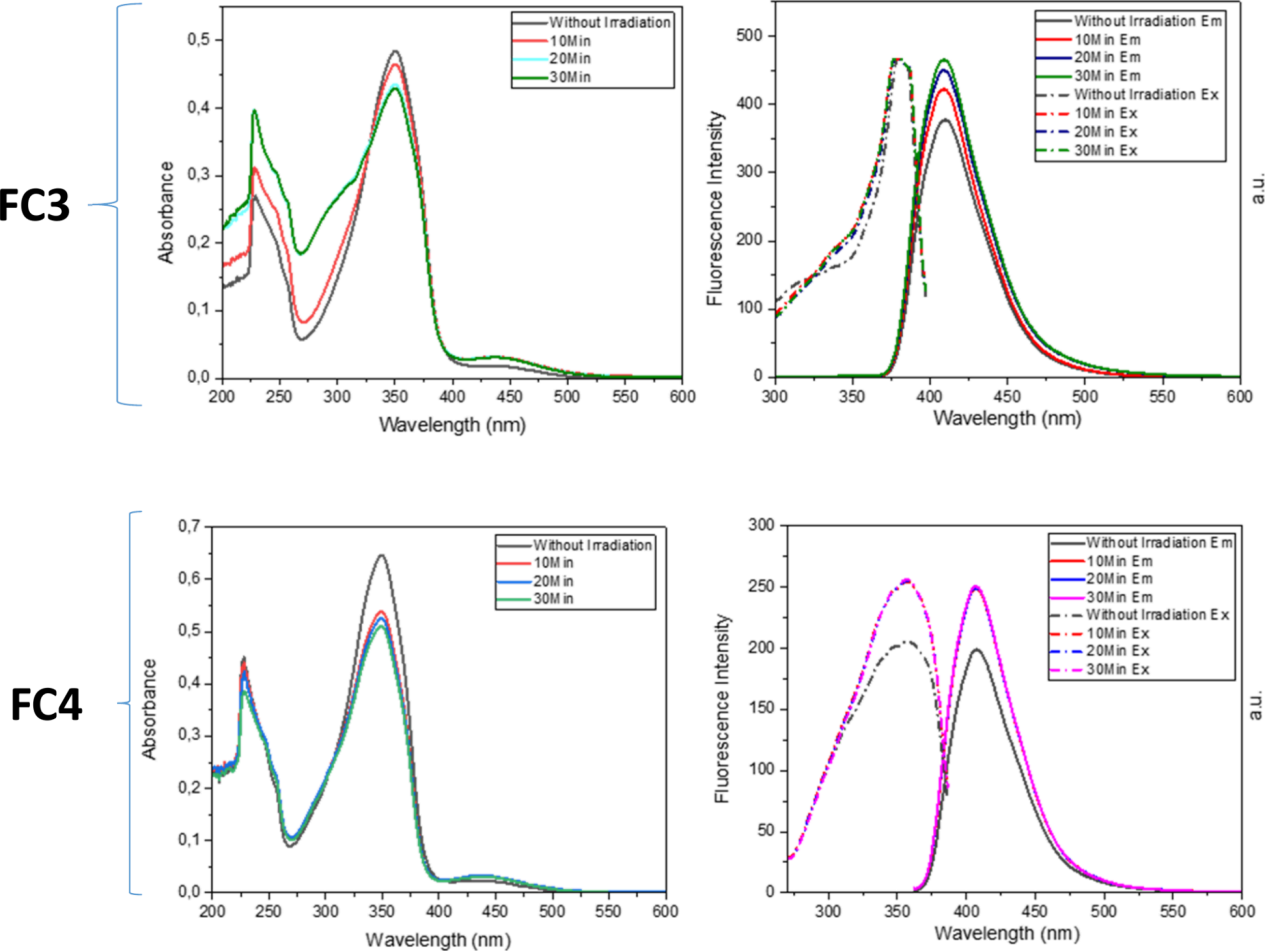
UV–vis and fluorescence spectra of **FC3** and **FC4** in DCM (10^–5^ M) before and after irradiation.

With all of these results in mind, it was decided that the coumarin absorption should be shifted to the bathochromic region compared to the azobenzene absorption region. This shift, in turn, creates an absorption spectrum on which the chromophore units will have separated absorptions. With this, we could control *trans*–*cis* isomerization better without disturbing the coumarin unit’s absorption region so that *trans*–*cis* isomerization interfering with coumarin unit absorption will have a minimum value. For this purpose, we synthesized **FC5** and **FC6**. Installing the diethylamino group at the seventh position of the coumarin shifted the absorption of the coumarin unit to the red region. This choice separated the coumarin chromophore from the azobenzene chromophore in the UV–vis spectrum (Figure 9). Irradiating the compound in DCM at 365 nm led to a disappearance of the absorption at 340 nm (azobenzene region). This shows that the *trans*-azobenzene side chain converted into its *cis* form. The same pattern was observed for different solvents (Supporting Information). As to the emission properties of these compounds with azobenzene in the *trans* form and *cis* form, the spectra were recorded without and after irradiation. When compounds were excited with light of λ_max_ = 398 nm, we observed fluorescence at around 455 nm. Contrary to previous observations, azobenzene in the *cis* form caused a decrease in the fluorescence intensity (Figure 9).

**Figure 9:**
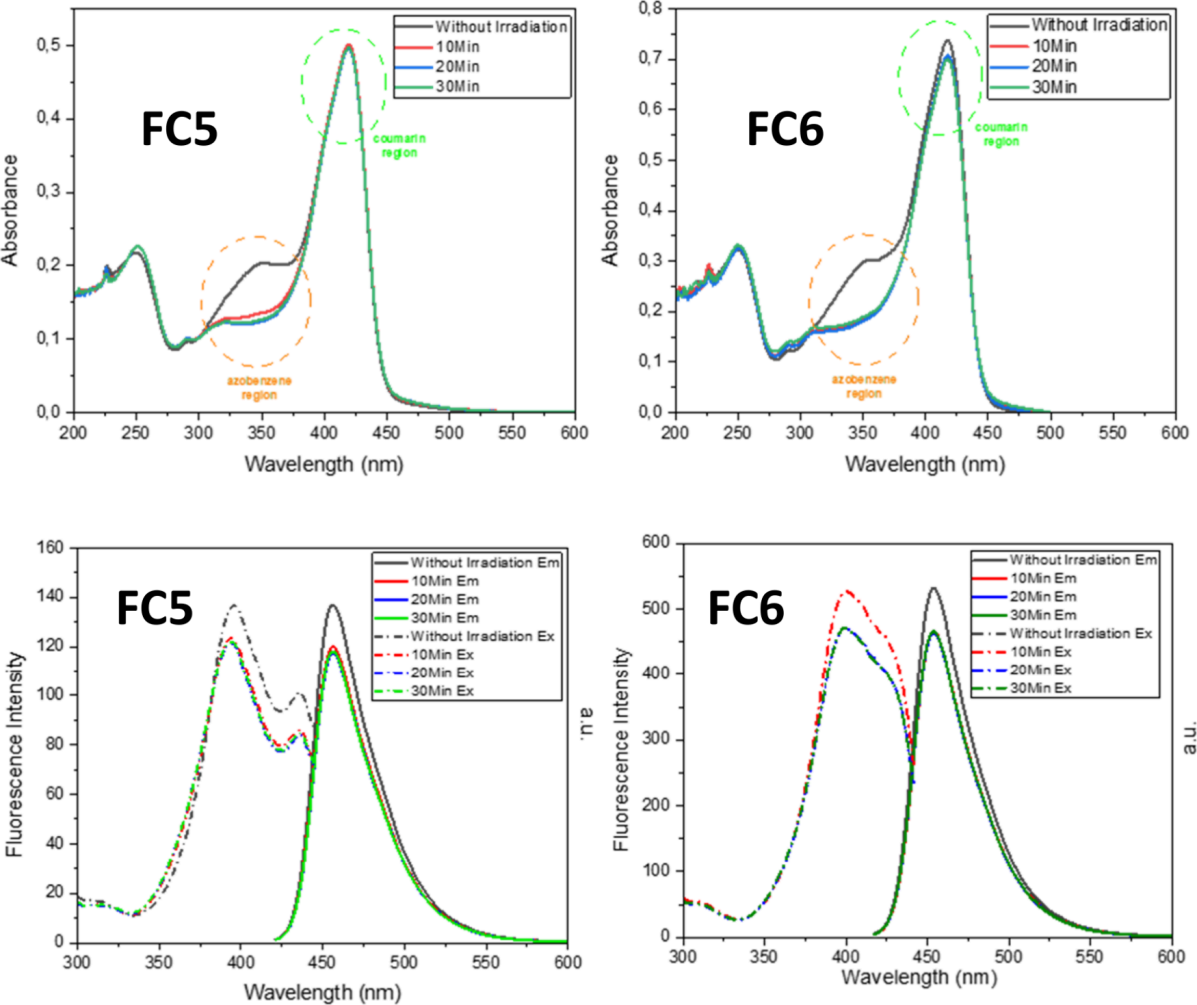
UV–vis and fluorescence spectra of **FC5** and **FC6** in DCM (10^–5^ M) before and after irradiation.

### Theoretical Calculations

To explain this observed abnormal effect of *trans*–*cis* isomerization of these compounds, theoretical calculations were employed. The geometries of all the above-mentioned compounds were optimized at the B3LYP/6-31g­(d) level of theory as implemented in Gaussian 09.^43^ The *cis* forms of these compounds were also optimized at the same level. In order to shine light on the fluorescence properties observed above, we also performed population analysis at the same level of theory and time-dependent DFT calculations at the CAM-B3LYP/6-31g­(d) level of theory. To minimize the computation time for 7-butoxycoumarin compounds (**FC3** and **FC4**), the butyl group at the seventh position of these derivatives was replaced with the methyl group. Optimization shows that the compounds did not aggregate within themselves. This shows that there is no force to stack the coumarin unit with the azobenzene unit. The basic assumption in our calculations is that the octyl linker will not affect the photophysical properties. It was given that no interaction between the azo and coumarin units was observed for the calculations of the propyl linker unit. Furthermore, the frontier orbitals for the compounds with an octyl linker (**FC2**, **FC4**, and **FC6**) show the exact same ordering as with the compounds with a propyl linker. This is consistent with the experimental observations where the octyl linker and the propyl linker compounds’ photophysical responses are the same.

Population analysis was done at the B3LYP/6-31g­(d) level for these optimized geometries. The visualization of molecular orbitals was done by using Jmol.^44^ For the azobenzene unit at its *trans* form, attached to the seventh and third (Figure 10) position of coumarin, this resulted in the same frontier orbitals; that is, HOMO–1 is located on nonbonding orbitals of azo units, the HOMO is located on the π MO of the azo unit, the LUMO is located on coumarin, and LUMO+1 is located on the π* MO of the azo unit. It is more clearly seen that the frontier molecular orbitals of **FC1** and **FC3**, where the coumarin of the azobenzene unit is located at the seventh and third position, respectively, are not only located at the same places but also have similar orbital energies (Figure 10).

**Figure 10:**
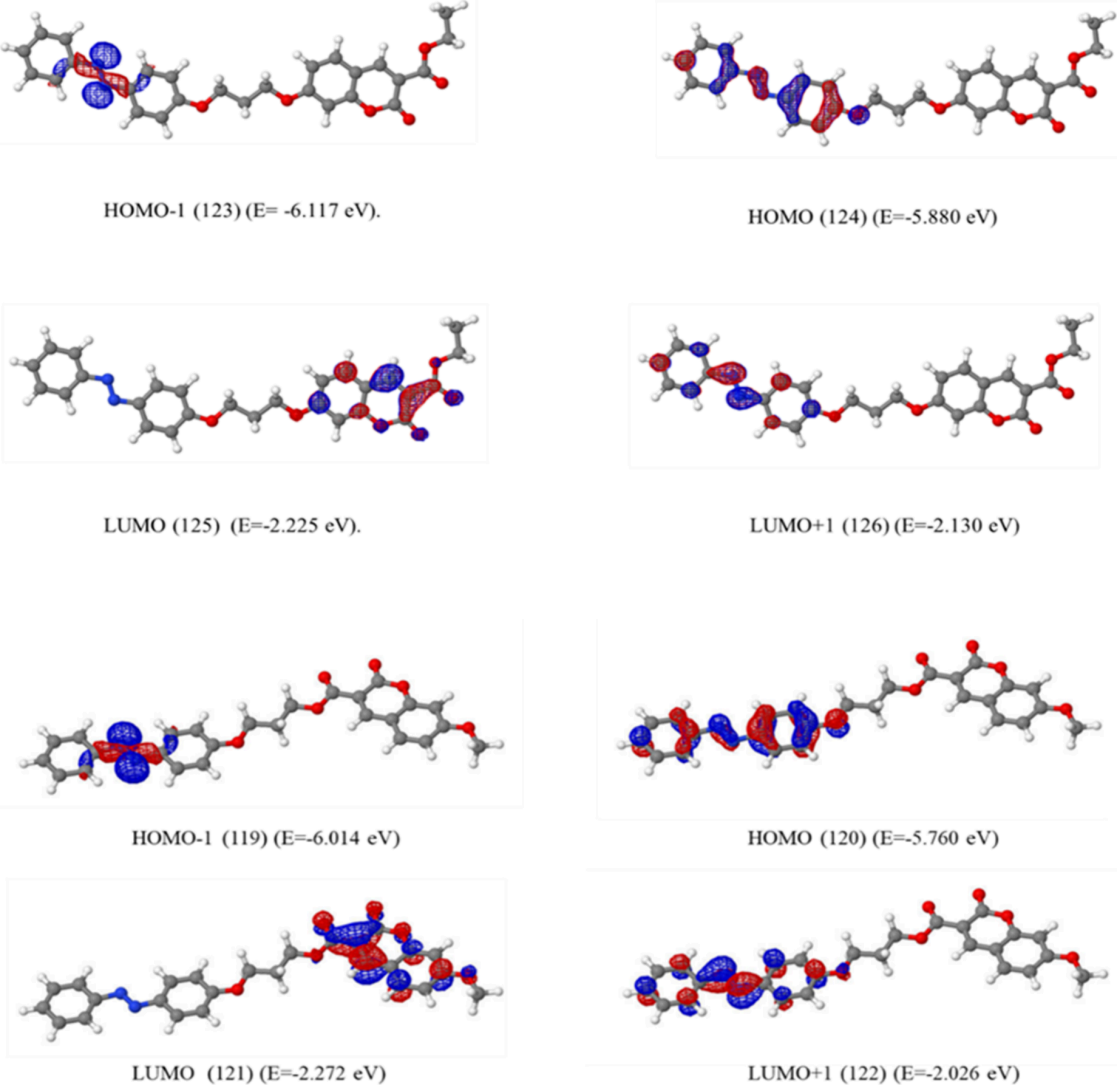
Calculated frontier orbitals of **FC1** and **FC3** in their *trans* form.

For the *cis* form of these compounds (**FC1** and **FC3**), the HOMO, LUMO, and LUMO+1 stayed in the same order, while HOMO–1 is now located on coumarin for both. This could be easily understood due to the conjugation being disrupted for the *cis* form on the azobenzene unit (Figure 11). The calculated frontier orbital energy resemblance for the *trans* form was also observed in the *cis* form of these substances, expectedly.

**Figure 11:**
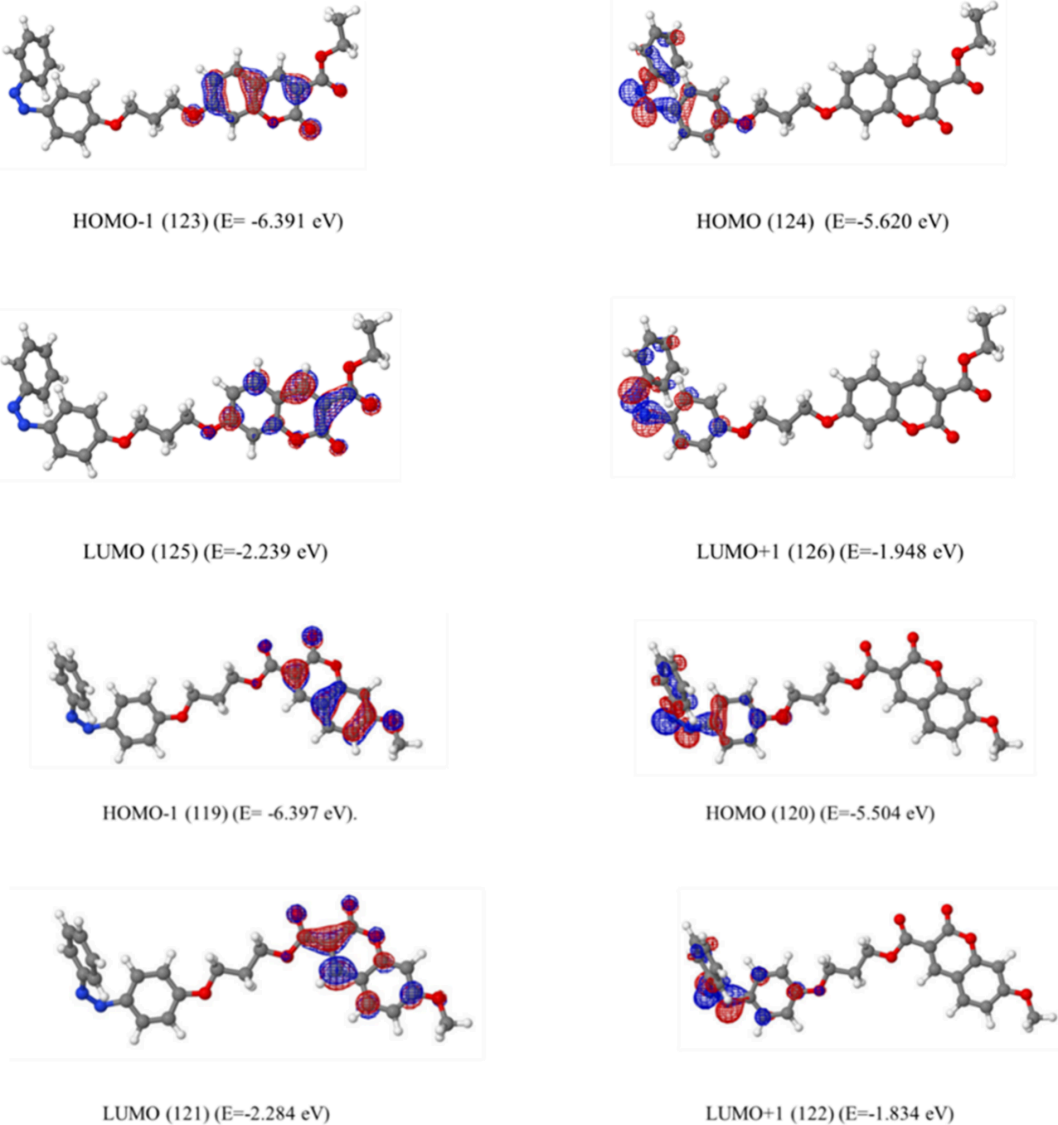
Calculated frontier orbitals of **FC1** and **FC3** in their *cis* form.

For the 7-(diethylamino)­coumarin derivative compound **FC5**, the coumarin unit has more contribution to the frontier molecular orbitals that the HOMO and LUMO+1 are located on coumarin, while HOMO–1 and LUMO are located on the azobenzene unit for the *trans* form (Figure 12). In the *cis* form, the contribution of chromophores to the molecular orbitals exchanged; that is, HOMO–1 and LUMO are located on coumarin while the HOMO and LUMO+1 are located on the azobenzene unit.

**Figure 12:**
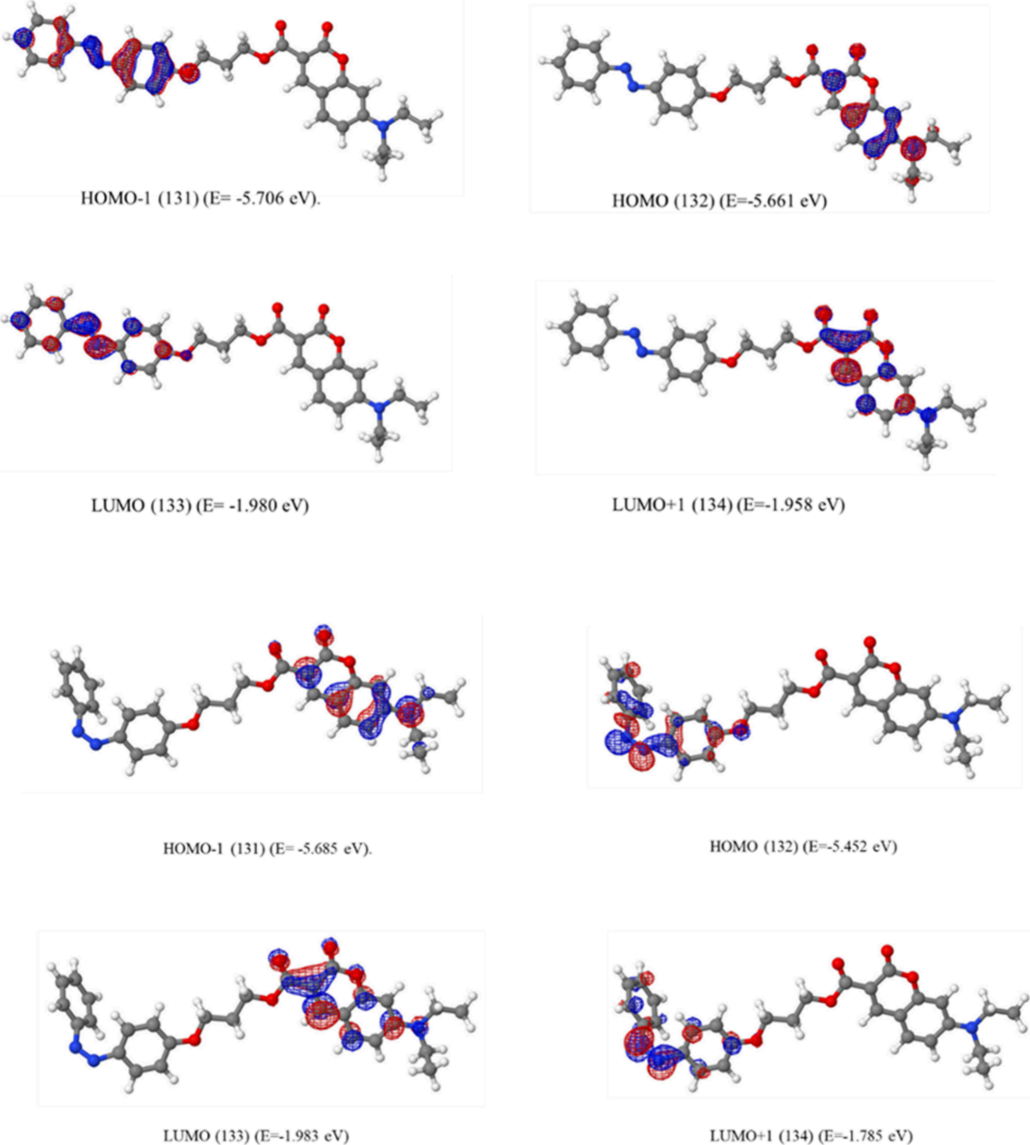
Calculated frontier orbitals of **FC5** in its *trans* and *cis* form.

Time-dependent DFT calculations (CAM-B3LYP/6-31g­(d)) were carried out for these compounds. The results for the corresponding transitions are tabulated in Table 1. Due to the computational cost, we calculated only three vertical excitations to compare the results qualitatively. Vertical transitions show that the transitions are isolated; that means that transitions from the azobenzene unit to the coumarin unit or vice versa are not observed. This is consistent with the experimental observations. As we irradiated *trans*-azobenzene compounds **FC1**, **FC3**, and **FC5** with 365 nm UV light to convert them into the *cis* form, there is no change on the UV–vis spectrum for the corresponding coumarin region. The same is valid for the amino compounds’ coumarin region. These results imply no effect of *cis* and *trans* isomerizations on fluorescence.

**Table 1:** Calculated Vertical Transitions^a^

	***trans***			***cis***		
**compound**	**λ (nm)**	***f* (oscillator strength)**	**transition**	**λ (nm)**	***f* (oscillator strength)**	**transition**
**FC1**	448	0.0000	122–126	467	0.0415	124–126
	320	1.2631	124–126	302	0.6403	123–125
	302	0.3789	123–125	280	0.0010	118–125
**FC3**	476	0.0000	119–122	485	0.0585	120–122
	383	0.0006	120–121	415	0.0001	120–121
	351	0.0000	119–121	328	0.0010	114–121
**FC5**	497	0.0000	130–133	510	0.0491	132–134
	354	1.4709	132–133	348	0.1285	132–133
	346	0.2389	131–134	347	0.6462	131–133

a In **FC1**, 124 is the HOMO, and 125 is the LUMO; in **FC3**, 120 is the HOMO, and 121 is the HOMO; in **FC5**, 132 is the HOMO, and 133 is the LUMO.

With experimental and theoretical results in mind, there was no obvious explanation for the fluorescence intensity increase in **FC1**, **FC2**, **FC3**, and **FC4** and the fluorescence decrease for **FC5** and **FC6**. Theoretical calculations show that the *trans*–*cis* azobenzene conversion does not have any effect on vertical excitations and thus on fluorescence. When we re-examined our experimental results for **FC1**–**4**, we realized that, in the realm of excitation wavelengths, the *trans*-azobenzene unit has an absorption. In the *cis* form, that absorption vanishes; thus, more photons are available for exciting coumarin units, which in turn resulted in a fluorescence intensity increase. On the other hand, for the *trans* form, the number of photons reaching to the coumarin moiety is less than that of the *cis* form, which leads to a lower fluorescence intensity compared to the *cis* form. As a result, as the *trans*-azobenzene converted to the *cis* form, a fluorescence increase was observed for **FC1**, **FC2**, **FC3**, and **FC4**. Furthermore, we have taken the excitation and emission spectra of **FC3** in three different concentrations (Supporting Information). We observed that fluorescence increases in the *cis* form in all concentrations.

As for **FC5** and **FC6**, a fluorescence intensity decrease should be explained differently because the excitation wavelength is not in the region of the azobenzene absorption band. As we converted the *trans*-azobenzene unit of **FC5** into the *cis* form, the n → π∗ band (around 410–450 nm) increased. That region falls into the emitting region of 7-(diethylamino)­coumarin units. This shows that in coumarin fluorescence, the n → π∗ region absorbs these photons. This, in turn, leads to a decrease in the fluorescence of **FC5** and **FC6**.

## Conclusions

In this study, six new coumarin-conjugated azobenzene derivatives were successfully synthesized to explore their optical properties. The structural adjustments were focused on the positional variations of the azobenzene at the 3- and 7-positions and the distance between the coumarin core and azobenzene unit. Their effects on fluorescence were studied experimentally and theoretically. A key finding was that the substitution of amino or butoxy units at the seventh position of the coumarin core led to distinct fluorescence behaviors, that is, as *trans*–*cis* isomerization occurred, fluorescence enhancement was observed when the seventh position of the coumarin unit was capped with butoxy, while a decrease in fluorescence was observed in 7-(diethylamino)­coumarin derivatives. Theoretical studies and experiments in various solvents allowed us to conclude that there is no charge transfer and that chromophoric units act independently. Additionally, computational studies showed that fluorescence is not directly impacted by the *trans*–*cis* isomerization of azobenzene. Experimental findings revealed that azobenzene absorbs at the excitation wavelength in the *trans* form, lowering the amount of photons that reach the coumarin unit; in contrast, this absorption disappears in the *cis* form, increasing the fluorescence for **FC1**–**FC2** and **FC3**–**FC4**. In contrast, compounds **FC5** and **FC6** undergo a fluorescence intensity decrease as a result of the *trans*–*cis* conversion. This decrease was attributed to increases in the n → π* absorption for the *cis* form in the emission region of the 7-(diethylamino)­coumarin unit. In light of these findings, it can be concluded that the utilization of azobenzene-coumarin derivatives, whose absorption regions intersect, can result in an increase in fluorescence. Conversely, the separation of the absorption regions through the use of an electron-rich system can lead to a decrease in fluorescence. In regard to these considerations, it can be stated that the development of new systems that can be easily obtained synthetically could enable the control of fluorescence.

## Experimental Section

The experimental conditions and the instruments used for the measurements are given in the Supporting Information.

### Synthesis of (*E*)-4-(Phenyldiazenyl)­phenol (**1**)

Aniline (5 g, 53.68 mmol) was dissolved in 20 mL of concentrated HCl and 20 mL of distilled water in a round-bottom flask placed in an ice bath. A solution of sodium nitrite (5 g, 72.47 mmol) in 20 mL of water was added to the former stirring solution for diazotization to occur. In another flask, a solution of phenol (5.8 g, 61.63 mmol) in 50 mL of 10% NaOH was prepared. This solution also was cooled to 5 °C by immersion in an ice bath. After 30 min, the phenol solution was strongly stirred along with the slow addition of cold diazonium salt solution. A dark orange-yellow solid product soon appeared and precipitated. The mixture was filtered through a funnel, and the solid product on filter paper was washed several times with cold water and dried for isolation (8.97 g, 90%). IR: 1470 cm^–1^ (N=N), 2800–3200 cm^–1^ (OH).

^1^H NMR (400 MHz, CDCl_3_): δ 7.88 (d, *J* = 4.6 Hz, 2H), 7.86 (d, *J* = 3.0 Hz, 2H), 7.50 (m, 2H), 7.44 (m, 1H), 6.94 (d, *J* = 8.77 Hz, 2H).

### Synthesis of (*E*)-3-(4-(Phenyldiazenyl)­phenoxy)­propan-1-ol (**1a**)

4-Hydroxy azobenzene (**1**) (1.98 g, 9.98 mmol) and 3-bromo-1-propanol (1.08 mL, 12.01 mmol) were dissolved in 30 mL of DMF. K_2_CO_3_ (2.07 g, 14.97 mmol) was added to this solution, and the solution was stirred at 75 °C for 12 h. After cooling to the RT, the solution was poured into 50 mL of cold water and extracted with 50 mL of chloroform. The organic phase was washed with 30 mL of 1 M HCl and 30 mL of brine solution and then dried over anhydrous MgSO_4_. The solvent evaporated under vacuum, and the orange solid product isolated (silica, EtOAc/Hex 1:3–1) (1.90 g, 74%). IR: 1494 cm^–1^ (N=N), 2800–3300 cm^–1^ (OH).

^1^H NMR (100 MHz, CDCl_3_): δ 7.90 (dd, *J* = 9.08, 8.9 Hz, 4H), 7.50 (t, *J* = 7.5 Hz, 2H), 7.44 (t, *J* = 7.2, 1H), 7.02 (d, *J* = 9.1, 2H), 4.20 (t, *J* = 5.9 Hz, 2H), 3.90 (t, *J* = 5.2, 2H), 2.09 (m, 2H), 1.64 (s, 1H).

### Synthesis of (*E*)-8-(4-(Phenyldiazenyl)­phenoxy)­octan-1-ol (**1b**)

The same procedure with the synthesis of (*E*)-3-(4-(phenyldiazenyl)­phenoxy)­propan-1-ol (**1a**) was applied with 8-chloro-1-octanol as the starting material. The orange solid product was obtained (silica, EtOAc/Hex 1:3–1) (0.67 g, 68%) (m.p.: 91.3 °C). IR: 1472 cm^–1^ (N=N), 2800–3280 cm^–1^ (OH).

^1^H NMR (400 MHz, CDCl_3_): δ 7.91 (dd, *J* = 8.9, 2.2 Hz, 2H), 7.85 (m, 2H), 7.50 (td, *J* = 8.0 Hz, 2H), 7.43 (m, 1H), 7.00 (dd, *J* = 9.1, 2.0 Hz, 2H), 4.05 (t, *J* = 6.32 Hz, 2H), 3.65 (q, *J* = 6.3, 2H), 1.8 (m, 2H), 1.5 (m, 2H), 1.37 (m, 8H).

^13^C NMR (100 MHz, CDCl_3_): δ 160.38, 151.42, 145.57, 128.97, 127.70, 123.39, 121.20, 113.33, 31.49, 27.98, 27.81, 24.60, 24.33.

### Synthesis of (*E*)-3-(4-(Phenyldiazenyl)­phenoxy)­propyl 4-Methylbenzenesulfonate (**2a**)

**1a** (0,82 g, 3.19 mmol) was dissolved in 5 mL of pyridine and cooled to 0 °C in an ice bath for 30 min, and then, tosyl chloride (0.7 g, 3.67 mmol) was added slowly to the stirring solution. The solution was kept in an ice bath for 3 h while reaction was proceeding. After 3 h, 30 mL of ice–water was added to quench the reaction. The precipitated solid was filtered and washed several times with cold water. Afterward, the solid was collected on the filter paper dissolved in DCM and extracted with 30 mL of 0.1 M H_2_SO_4_. The compound was dried over MgSO_4_, and the solvent evaporated under vacuum. A brownish-orange solid product was isolated purely (0.95 g, 73%). IR: 1466 cm^–1^ (N=N), 1746 cm^–1^ (C=O), 2939 cm^–1^ (methyl group of *p*-toluene from the tosyl group).

^1^H NMR (400 MHz, CDCl_3_): δ 7.80 (m, 3H), 7.45 (m, 3H), 7.23 (d, *J* = 7.52 Hz, 2H), 6.85 (d, *J* = 8.84, 2H), 4.26 (t, *J* = 6.48 Hz, 2H), 4.02 (t, *J* = 5.56, 2H), 2.35 (s, 3H), 2.15 (t, *J* = 5.36, 2H), 1.37 (m, 8H).

### Synthesis of (*E*)-8-(4-(Phenyldiazenyl)­phenoxy)­octyl 4-Methylbenzenesulfonate (**2b**)

The same procedure with the synthesis of (*E*)-3-(4-(phenyldiazenyl)­phenoxy)­propyl 4-methylbenzenesulfonate was applied with (*E*)-8-(4-(phenyldiazenyl)­phenoxy)­octan-1-ol (**1b**) as a starting material (0.71 g, 71%) (m.p.: 101.8 °C). IR: 1463 cm^–1^ (N=N), 1603 cm^–1^ (C=O), 2939 cm^–1^ (methyl group of *p*-toluene from the tosyl group).

^1^H NMR (400 MHz, CDCl_3_): δ 7.90 (m, 4H), 7.80 (d, *J* = 8.16, 2H), 7.49 (m, 4H), 7.35 (d, *J* = 8.72, 1H), 7.00 (d, *J* = 9.12 Hz, 2H), 4.02 (t, *J* = 6.50, 4H), 2.95 (s, 3H), 1.80 (m, 3H), 1.30 (m, 10H).

^13^C NMR (100 MHz, CDCl_3_): δ 161.66, 152.73, 146.80, 144.66, 133.25, 130.29, 129.73, 129.15, 127.91, 124.60, 122.43, 114.57, 70.61, 68.19, 29.12, 28.84, 28.81, 25.87, 25.25, 21.65.

HRMS (ESI/MS) *m*/*z*: [M + H]^+^ calcd for C_27_H_33_N_2_O_4_S^+^, 481.2161; found, 481.2161.

### Synthesis of Ethyl 7-Hydroxy-2-oxo-2*H*-chromene-3-carboxylate (**C1**)

2,4-Dihydroxybenzaldehyde (1.21 g, 8.77 mmol) and diethyl malonate (1.46 mL, 9.57 mmol) were dissolved in 15 mL of EtOH. Piperidine (0.37 mL) and acetic acid (0.15 mL) were added to this stirring solution, and reaction was carried out under reflux conditions for 18 h. EtOH was evaporated under vacuum, and an oily residue was poured into an ice–water mixture. The precipitated solid was filtered and recrystallized from EtOH. White crystals were dried and isolated (1.80 g, 88%). ^1^H NMR (400 MHz, DMSO-*d*_6_): δ 8.57 (s, 1H), 7.66 (d, *J* = 8.20, 2H), 6.63–6.75 (m, 2H), 4.15 (q, *J* = 7.88, 2H), 1.20 (t, *J* = 6.20, 3H).

### Synthesis of **FC1**

Ethyl 7-hydroxy-2-oxo-2*H*-chromene-3-carboxylate (**C1**) (0.50 g, 2.20 mmol) and K_2_CO_3_ (0.35 g, 2.53 mmol) were dissolved in 25 mL of DMF under slow heating to 60 °C. After an hour, (*E*)-3-(4-(phenyldiazenyl)­phenoxy)­propyl 4-methylbenzenesulfonate (**2a**) (0.90 g, 2.20 mmol) and KI (0.28 g, 1.68 mmol) were added to the mixture and heat rose to 75 °C. After 18 h, the reaction mixture was cooled to RT and then poured into 50 mL of cold water. After precipitation finished, it was filtered and the solid was washed several times with cold water. The compound was recrystallized from EtOH, and a dark orange solid was dried and isolated (0.42 g, 40%) (m.p.: 107.9 °C). IR: 1468 cm^–1^ (N=N), 1750 cm^–1^ (C=O), 2881 cm^–1^ (C–H).

^1^H NMR (400 MHz, CDCl_3_): δ 8.50 (s, 1H), 7.89 (dd, *J* = 9.32, 7.56, 4H), 7.50 (m, 4H), 7.02 (d, *J* = 8.74, 2H), 6.91 (dd, *J* = 1.84 Hz, 1H), 6.85 (s, 1H), 4.40 (q, *J* = 7.28, 6.84, 2H), 4.27 (q, *J* = 7.92, 8.28, 4H), 2.35 (m, 2H), 1.40 (t, *J* = 6.6, 3H).

^13^C NMR (100 MHz, CDCl_3_): δ 188.28, 164.33, 163.46, 161.15, 157.53, 152.75, 148.97, 147.09, 130.80, 130.44, 129.02, 124.81, 122.57, 114.76, 113.81, 111.70, 101.08, 65.18, 64.29, 61.73, 28.87, 14.37.

HRMS (TOF/MS) *m*/*z*: [M + H]^+^ calcd for C_27_H_25_N_2_O_6_^+^, 473.1714; found, 473.1713.

### Synthesis of **FC2**

The same procedure with the synthesis of **FC1** was applied with (*E*)-8-(4-(phenyldiazenyl)­phenoxy)­octyl 4-methylbenzenesulfonate (**2b**) as a starting material (0.095 g, 32%) (m.p.: 114.6 °C). IR: 1473 cm^–1^ (N=N), 1745 cm^–1^ (C=O), 2853 and 2936 cm^–1^ (C–H).

^1^H NMR (400 MHz, CDCl_3_): δ 8.50 (s, 1H), 7.89 (dd, *J* = 7.68, 8.92, 4H), 7.48 (m, 4H), 7.00 (d, *J* = 8.96, 2H), 6.87 (m, 1H), 6.79 (d, *J* = 2.12, 1H), 4.39 (q, *J* = 7.08, 7.06, 2H), 4.04 (m, 4H), 1.83 (m, 4H), 1.41 (m, 11H).

^13^C NMR (100 MHz, CDCl_3_): δ 164.73, 163.51, 161.64, 157.60, 157.25, 152.76, 149.04, 146.86, 130.66, 130.32, 129.02, 124.74, 122.52, 114.68, 114.03, 113.88, 111.47, 100.76, 68.90, 68.25, 61.69, 29.24, 29.21, 29.15, 28.85, 25.94, 25.86, 14.29.

HRMS (TOF/MS) *m*/*z*: [M + H]^+^ calcd for C_32_H_35_N_2_O_6_^+^, 543.2495; found, 543.2495.

### Synthesis of 7-Butoxy-2-oxo-2*H*-chromene-3-carboxylic Acid (**C1a**)

Ethyl 7-hydroxy-2-oxo-2*H*-chromene-3-carboxylate (**C1**) (0.23 g, 1.00 mmol) and K_2_CO_3_ (0.20 g, 1.50 mmol) were dissolved in 7 mL of dry DMF and allowed to stir for an hour with heating to 45 °C (the temperature should not be above 60 °C). After an hour, 1-bromobutane (0.13 mL, 1.20 mmol) was added portionwise to the reaction medium. The reaction was allowed to proceed for 12 h, and after controlling with TLC (EtOAc/Hex 1:1), the reaction was finished and cooled to RT. After cooling, the reaction mixture was poured into an ice–water mixture and filtered, washed with water several times, and dried. The obtained white solid (0.24 g, 0.82 mmol, 82%) was dissolved in 5 mL of 60% EtOH-water solution with KOH (0.08 g, 1.38 mmol). The reaction was allowed to proceed for 5 h at 50 °C and then an additional 1 h at RT. After completion of the reaction, EtOH was removed under vacuum, and the residual solution was acidified to pH = 1 with dilute HCl solution in an ice bath. The precipitated solid was filtered and dried to provide a white solid product (0.21 g, 96%). ^1^H NMR (400 MHz, DMSO-*d*_6_): δ 8.72 (s, 1H), 7.82 (d, *J* = 8.60, 1H), 6.98–7.01 (m, 2H), 4.11 (t, *J* = 6.48, 2H), 1.76 (m, 2H), 1.44 (m, 2H), 0.93 (t, *J* = 7.28, 3H).

### Synthesis of **FC3**

DMAP (0.09 g, 0.76 mmol) and EDC.HCl (0.55 g, 2.85 mmol) were successively added to a stirring solution of 7-butoxy-2-oxo-2*H*-chromene-3-carboxylic acid (**C1a**) (0.50 g, 1.90 mmol) and (*E*)-3-(4-(phenyldiazenyl)­phenoxy)­propan-1-ol (**1a)** (0.48 g, 1.90 mmol) in 20 mL of anhydrous DCM. The mixture was stirred at RT for 24 h under an argon atmosphere. After that, in DCM, extraction was performed with saturated NaHCO_3_ solution, water, and brine and Na_2_SO_4_ was used as a drying agent. The solvent was removed under vacuum, and the solid product was recrystallized from EtOH. Yellow crystals were isolated (0.42 g, 45%) (m.p.: 98.0 °C). IR: 1471 cm^–1^ (N=N), 1724 cm^–1^ (C=O), 2872 and 2959 cm^–1^ (C–H).

^1^H NMR (400 MHz, CDCl_3_): δ 8.50 (s, 1H), 7.90 (m, 4H), 7.50 (m, 4H), 7.04 (d, *J* = 9.04 Hz, 2H), 6.86 (dd, *J* = 6.32, 3.14 Hz, 1H), 6.79 (d, *J* = 2.40, 1H), 4.56 (t, *J* = 6.20 Hz 2H), 4.26 (t, *J* = 5.88 Hz 2H), 4.04 (t, *J* = 6.64 Hz 2H), 2.30 (m, 2H), 1.80 (m, 2H), 1.50 (m, 2H), 0.99 (t, *J* = 7.12, 3H).

^13^C NMR (100 MHz, CDCl_3_): δ 164.90, 163.70, 161.30, 157.69, 157.14, 152.76, 149.31, 147.05, 130.75, 130.36, 129.03, 124.78, 123.14, 122.75, 122.56, 114.79, 114.06, 113.61, 111.43, 100.80, 68.69, 64.74, 62.33, 30.88, 28.58, 19.14, 13.77.

HRMS (TOF/MS) *m*/*z*: [M + H]^+^ calcd for C_29_H_29_N_2_O_6_^+^, 501.2026; found, 501.2025.

### Synthesis of **FC4**

The same procedure with the synthesis of **FC3** was applied with the difference of usage of (*E*)-8-(4-(phenyldiazenyl)­phenoxy)­octan-1-ol (**2b**) as a starting material (0.40 g, 44%) (m.p.: 82 °C). IR: 1472 cm^–1^ (N=N), 1748 cm^–1^ (C=O), 2855 and 2939 cm^–1^ (C–H).

^1^H NMR (400 MHz, CDCl_3_): δ 8.49 (s, 1H), 7.90 (m, 4H), 7.50 (m, 4H), 7.00 (d, *J* = 9.05 Hz, 2H), 6.86 (dd, *J* = 6.44, 2.56 Hz, 1H), 6.78 (d, *J* = 2.40, 1H), 4.33 (t, *J* = 6.60 Hz 2H), 4.04 (m, 4H), 1.79 (m, 5H), 1.45 (m, 11H), 0.98 (t, *J* = 7.32, 3H).

^13^C NMR (100 MHz, CDCl_3_): δ 164.77, 163.65, 161.70, 157.62, 157.19, 152.80, 148.95, 146.86, 130.64, 130.28, 129.01, 124.74, 122.53, 114.70, 114.00, 111.46, 100.79, 68.67, 68.31, 65.74, 30.88, 29.19, 29.15, 29.12, 28.60, 25.92, 25.82, 19.13, 14.12, 13.76.

HRMS (TOF/MS) *m*/*z*: [M + H]^+^ calcd for C_34_H_39_N_2_O_6_^+^, 571.2808; found, 501.2807.

### Synthesis of 7-(Diethylamino)-2-oxo-2*H*-chromene-3-carboxylic Acid (**C2**)

4-Diethylaminosalicylaldehyde (0.96 g, 5.00 mmol) was dissolved in 3.3 mL of EtOH. Diethyl malonate (1 mL, 6.5 mmol) and 0.4 mL of piperidine were added to this solution, and the reaction mixture was refluxed for 3 h. After evaporation of EtOH, reaction medium was diluted with 50 mL of water and extracted with EtOAc (50 mL × 3). It was dried over MgSO_4_, and the solvent was removed under vacuum. The viscous product was purified with column chromatography (silica, EtOAc/Hex 1:5–1), and yellow crystals were obtained (0.75 g, 52%). Afterward, to a solution of this ester product (0.29 g, 1.0 mmol) in 10 mL of EtOH was added 10 mL of 0.5 M NaOH. The reaction was allowed to proceed at RT for 12 h, and after the completion of the reaction, EtOH was evaporated. After adding 5 mL of water to the remaining solution, acidification was performed with 1 M HCl until pH = 4. Then, with the completion of the precipitation, the solid was filtered and washed with water for a few times. The orange solid product was isolated (0.1 g, 40%). ^1^H NMR (400 MHz, CDCl_3_): δ 8.66 (s, 1H), 7.45 (d, *J* = 9.0 Hz, 1H), 6.71 (dd, *J* = 6.58, 4.76, 1H), 6.53 (d, *J* = 2.6 Hz, 1H), 3.49 (q, *J* = 7.12, 4H), 1.26 (t, *J* = 6.68, 6H).

### Synthesis of **FC5**

EDC·HCl (0.15 g, 0.76 mmol) and DMAP (0.03 g, 0.25 mmol) were added to a stirring solution of 7-(diethylamino)-2-oxo-2*H*-chromene-3-carboxylic acid (**C2**) (0.14 g, 0.51 mmol) and (*E*)-3-(4-(phenyldiazenyl)­phenoxy)­propan-1-ol (**1a**) (0.13 g, 0.51 mmol) in 10 mL of anhydrous DCM. The mixture was stirred at RT for 24 h under an argon atmosphere. After that, in DCM, extraction was performed with saturated NaHCO_3_ solution, water, and brine and Na_2_SO_4_ was used as a drying agent. The solvent was removed under vacuum, and the yellow solid product was isolated after column chromatography (silica, EtOAc/Hex 1:3–1) (0.12 g, 45%) (m.p.: 115.0 °C). IR: 1214 cm^–1^ (C–N (aromatic)), 1189 cm^–1^ (C–N (aliphatic)), 1465 cm^–1^ (N=N), 1725 cm^–1^ (C=O).

^1^H NMR (400 MHz, CDCl_3_): δ 8.40 (s, 1H), 7.88 (m, 4H), 7.45 (m, 3H), 7.33 (d, *J* = 9.32 Hz, 1H), 6.58 (dd, *J* = 6.56, 2.32 Hz, 1H), 6.45 (d, *J* = 2.36, 1H), 4.53 (t, *J* = 5.60 Hz, 2H), 4.26 (t, *J* = 6.32 Hz, 2H), 3.43 (q, *J* = 6.96, 7.16 Hz, 4H), 2.30 (m, 2H), 1.23 (m, 6H).

^13^C NMR (100 MHz, CDCl_3_): δ 164.30, 161.27, 158.45, 158.04, 152.84, 152.69, 149.23, 146.92, 130.97, 130.16, 128.87, 124.64, 122.42, 114.71, 109.43, 108.63, 107.59, 96.64, 64.82, 61.70, 44.97, 28.58, 12.31.

HRMS (TOF/MS) *m*/*z*: [M + H]^+^ calcd for C_29_H_30_N_3_O_5_^+^, 500.2185; found, 500.2185.

### Synthesis of **FC6**

The same procedure with the synthesis of **FC5** was applied with the difference of usage of (*E*)-8-(4-(phenyldiazenyl)­phenoxy)­octan-1-ol (**1b**) as a starting material (0.11 g, 38%). M.p.: 84.3 °C. IR: 1215 cm^–1^ (C–N (aromatic)), 1190 cm^–1^ (C–N (aliphatic)), 1471 cm^–1^ (N=N), 1747 cm^–1^ (C=O).

^1^H NMR (400 MHz, CDCl_3_): δ 8.42 (s, 1H), 7.90 (m, 4H), 7.46 (m, 3H), 7.34 (d, *J* = 8.84 Hz, 1H), 7.00 (d, *J* = 8.82 Hz, 2H), 6.58 (dd, *J* = 6.36, 2.36, 1H), 6.45 (d, *J* = 2.2 Hz 1H), 4.31 (t, *J* = 6.48 Hz 2H), 4.04 (t, *J* = 6.56 Hz 2H), 3.43 (q, *J* = 7.12, 7.24, 4H), 1.80 (m, 4H), 1.44 (m, 4H), 1.23 (m, 6H).

^13^C NMR (100 MHz, CDCl_3_): δ 164.39, 161.72, 158.48, 158.29, 152.84, 152.79, 149.15, 146.83, 131.02, 130.29, 129.03, 124.75, 122.53, 114.71, 109.47, 109.02, 107.68, 96.73, 68.33, 65.27, 45.09, 29.22, 29.17, 28.69, 25.93, 25.87, 12.44.

HRMS (TOF/MS) *m*/*z*: [M + H]^+^ calcd for C_34_H_40_N_3_O_5_^+^, 570.2968; found, 570.2968.

## Supplementary Material



## Data Availability

The data supporting this article have been included as part of the Supporting Information.

## Notes

Experiments, characterization raw data, UV–vis spectra, fluorescence spectra, *XYZ* coordinates of the optimized structures, wavelength profile of the irradiation source, and ^1^H NMR of the **FC3** before and after irradiation (PDF)
