# Supplementary material for: Impact of Azobenzene Side Chains on the Ultraviolet–Visible and Fluorescence Properties of Coumarin Derivatives
Source: ACS Omega. 2025 Oct 16;10(42):50326–39. doi: 10.1021/acsomega.5c07385 (PMC12573160; doi:10.1021/acsomega.5c07385)
Supplement: Supplementary file 1 [file ao5c07385_si_001.pdf]

Supporting Information

for

Impact of Azobenzene Side Chains on the UV-Vis and Fluorescence Properties of Coumarin Derivatives

Yasemin Akdis[1], Akin Akdag[1]*

[1]Department of Chemistry, Middle East Technical University, Ankara/Türkiye

*Corresponding author: aakdag@metu.edu.tr

Table of Contents

A. General Information....................2

B. NMR Spectra....................3

C. Infrared (IR) Spectra....................14

D. High Resolution Mass Spectra (HRMS)....................20

E. UV-Vis Spectra....................24

F. Fluorescence Spectra....................33

G. XYZ Coordinates of the Optimized Structures....................42

H. The Wavelength Profile of the Irradiation Source....................59

I. ^{1}H NMR of the FC3 Before and After Irradiation....................60

J. Concentration-Dependent Fluorescence Studies of FC3....................63

A. General Information

All reagents and solvents were sourced from Sigma Aldrich and used without further purification. Reaction progress was monitored via thin layer chromatography (TLC) using Merck Silica Gel 60 F254 plates and visualized under 254 nm UV light.

The UV lamp used for trans-cis isomerization has a wavelength of 365 nm (Philips, TL 8W BLB 1FM/10X25CC). The irradiation experiments were carried out in regular quartz cuvettes that have path length of 1 cm. The wavelength profile of the light source was given in the **H.**

^{1}H and ^{13}C nuclear magnetic resonance spectra of the compounds obtained using a Bruker Avance III Ultrashield 400 MHz spectrometer in deuterated solvents. Chemical shifts (in ppm) were referenced to tetramethylsilane (TMS). Spin multiplicities are denoted as s (singlet), d (doublet), dd (doublet of doublets), t (triplet), and m (multiplet), with coupling constants (*J*) reported in Hz. All NMR spectra were processed with the MestReNova program.

Infrared (IR) spectra were recorded with Thermo Scientific Nicolet iS10 ATR-IR spectrometer. Peak positions are reported in cm^{-1}. All IR spectra were processed using OriginPro 2024.

High-Resolution Mass Spectra (HRMS) were acquired in positive mode (ES^{+}) using a Time of Flight mass analyzer. Measurements were performed by METU-MERLAB.

UV-Vis measurements were performed using Shimadzu UV-2450 spectrophotometer and carried out in DCM, MeCN, THF, and MeOH (all of gradient grade). All UV-Vis spectra were processed using OriginPro 2024.

Fluorescence measurements were recorded using a Perkin Elmer LS55 spectrofluorometer and carried out in DCM, MeCN, THF, and MeOH (all of gradient grade). All fluorescence spectra were processed with OriginPro 2024.

All computational studies were performed using Gaussian 09. Initial structure optimizations were carried out using the B3LYP/6-31g(d) level of theory.

B. NMR Spectra

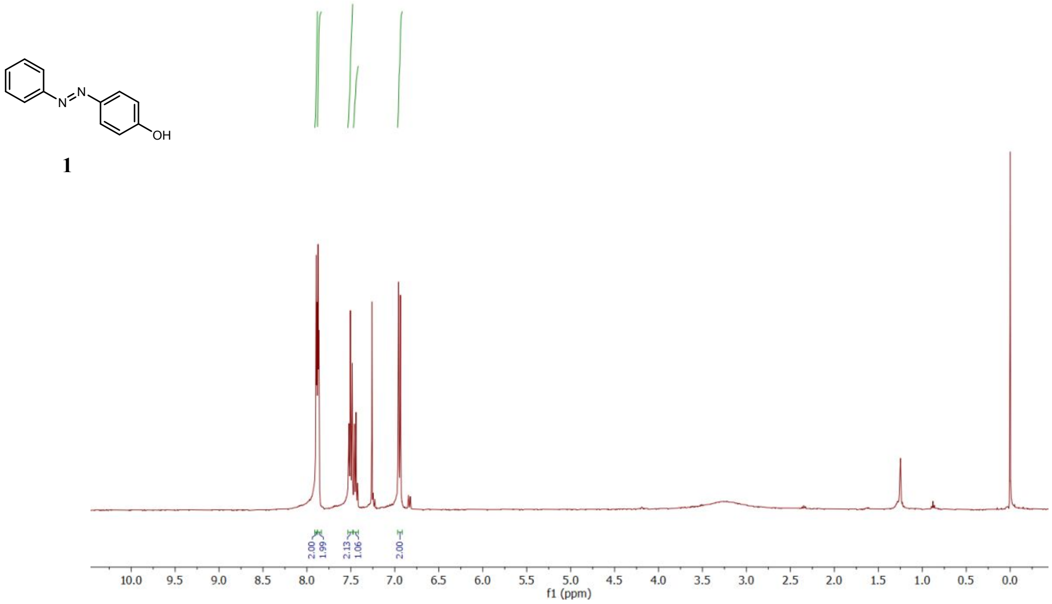

Figure S1. ^{1}H NMR of **1**

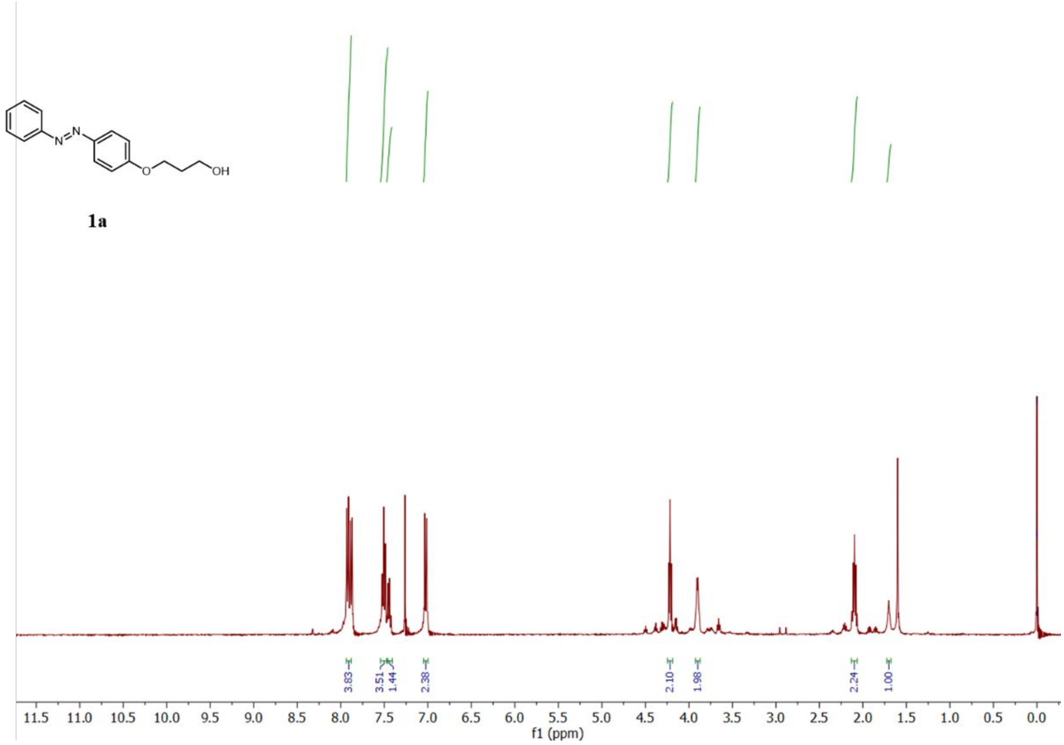

Figure S2. ^{1}H NMR of **1a**

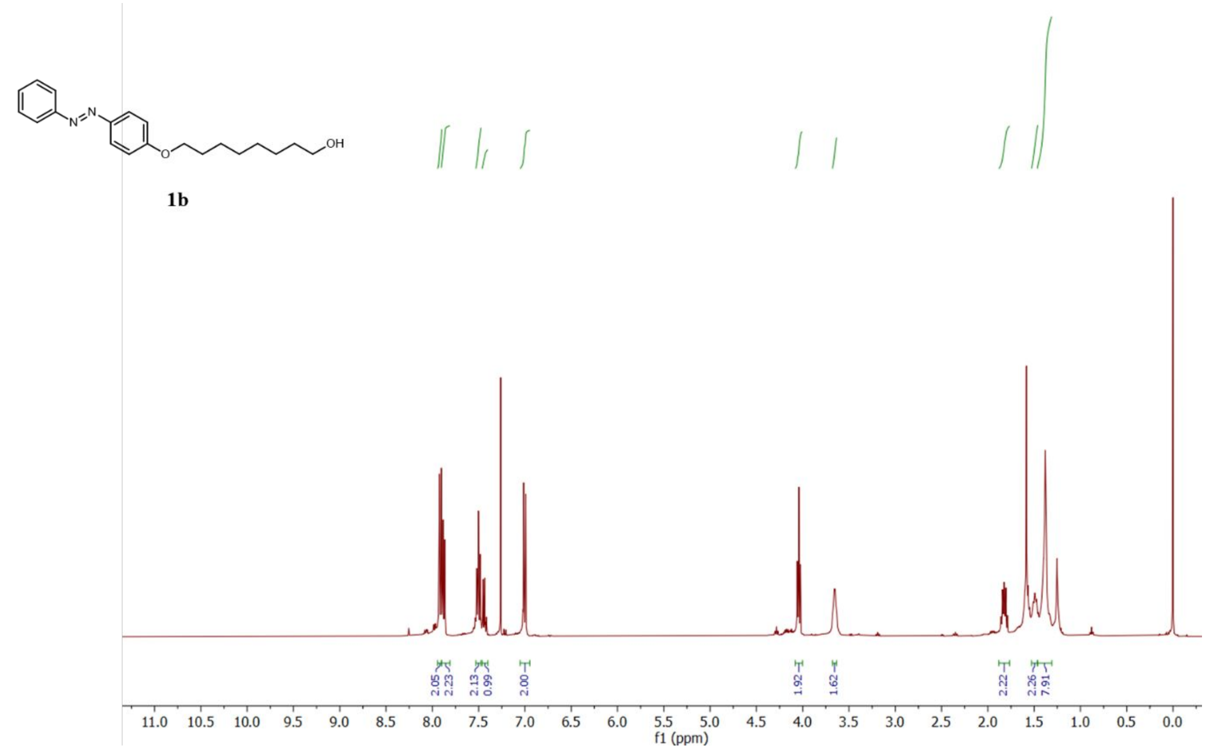

Figure S3. ^{1}H NMR of 1b

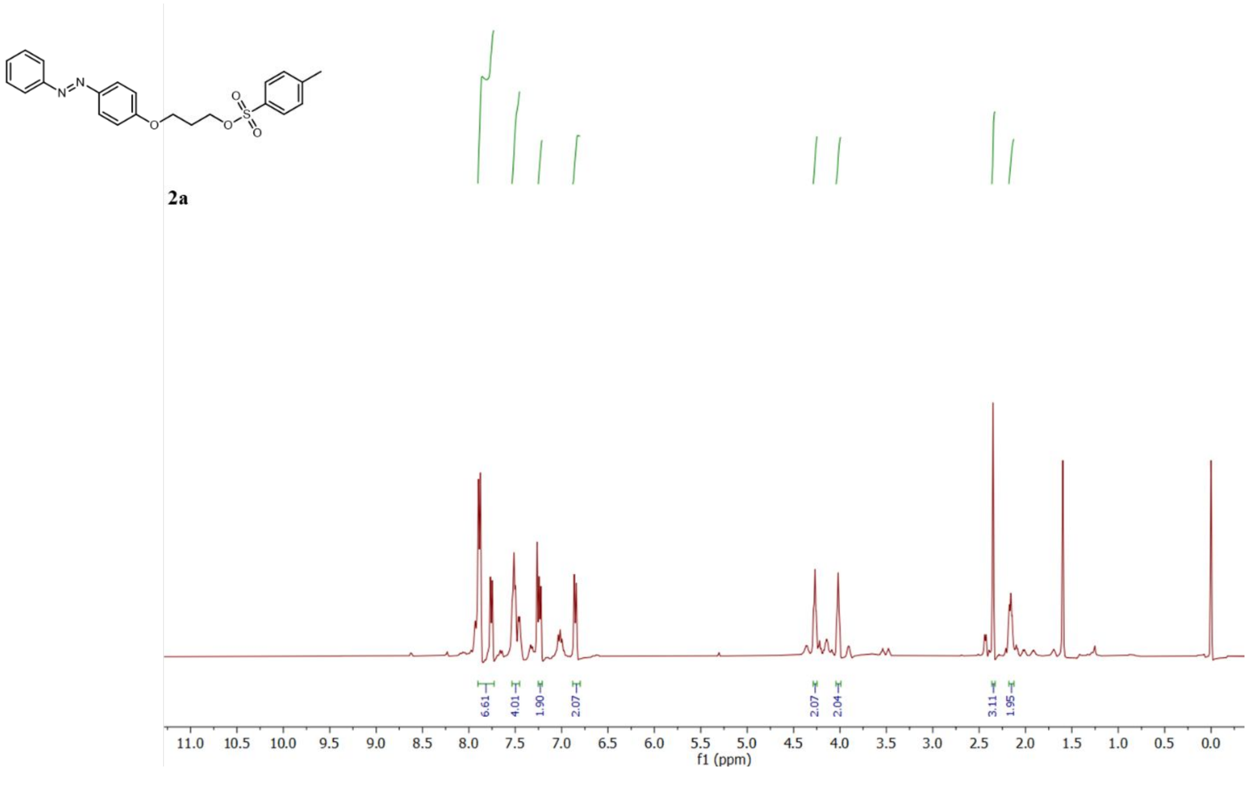

Figure S4. ^{1}H NMR of 2a

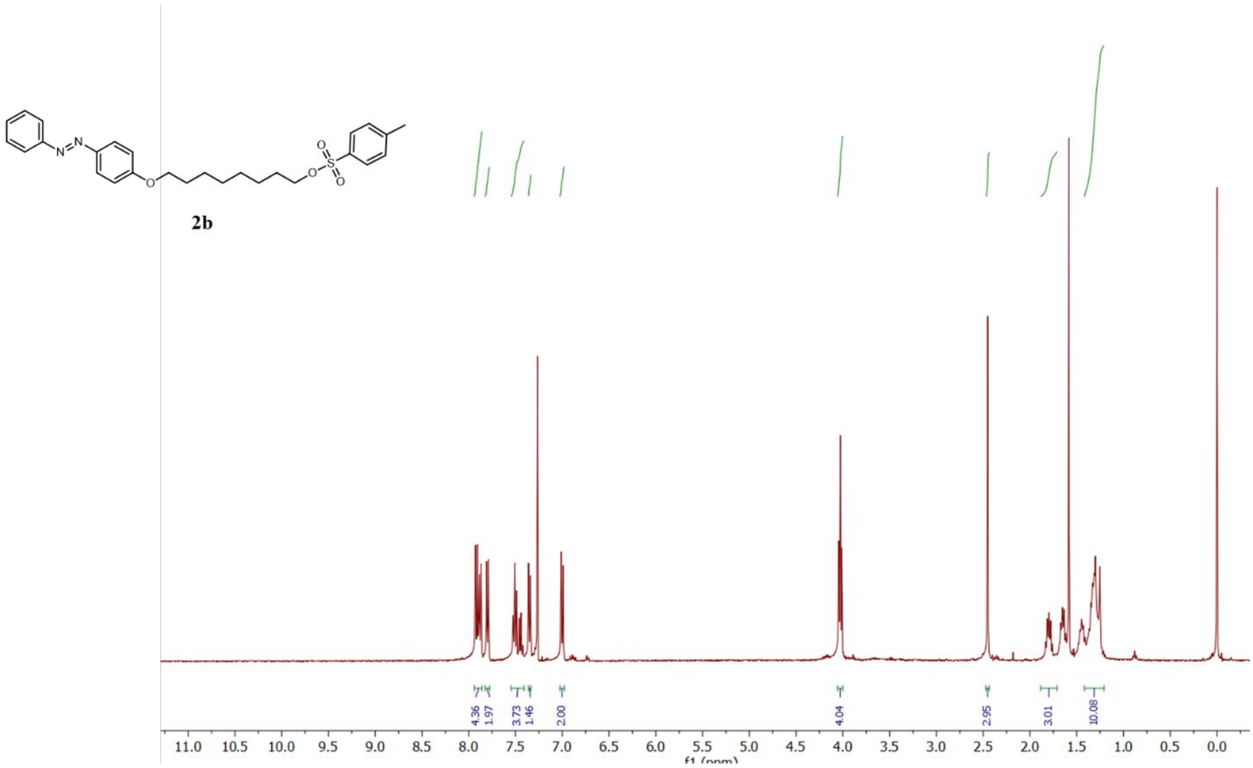

Figure S5. ^{1}H NMR of 2b

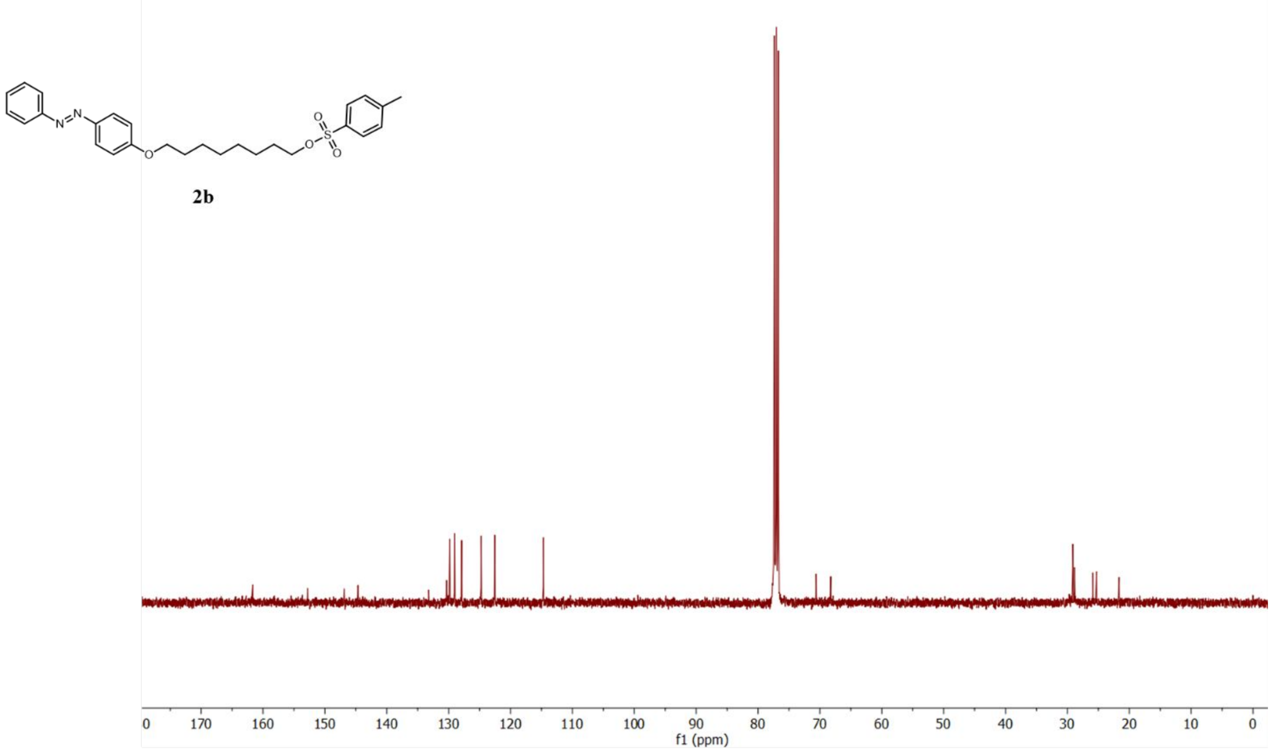

Figure S6. ^{13}C NMR of 2b

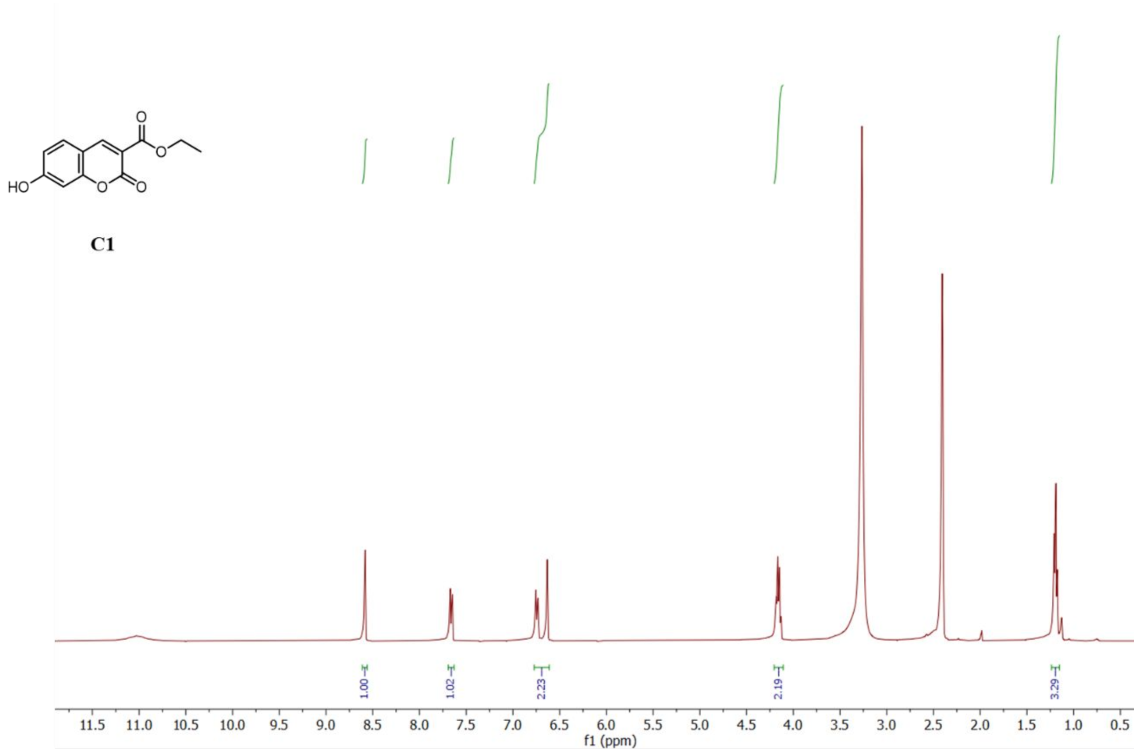

Figure S7. ^{1}H NMR of C1

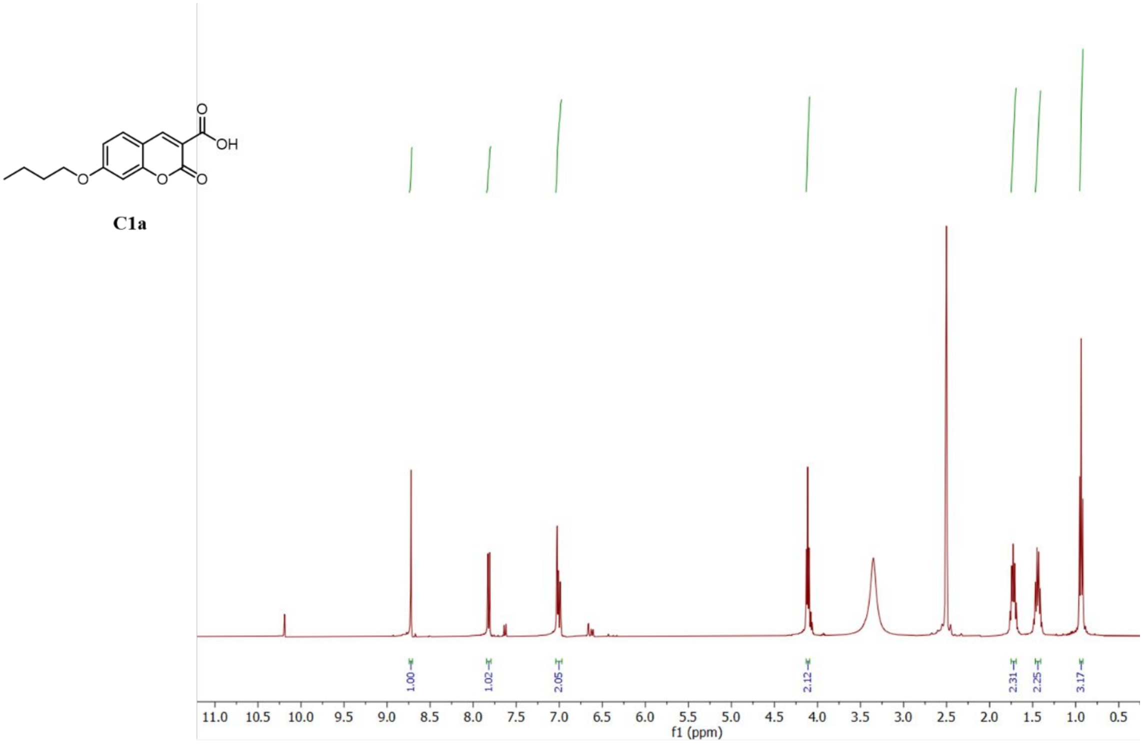

Figure S8. ^{1}H NMR of C1a

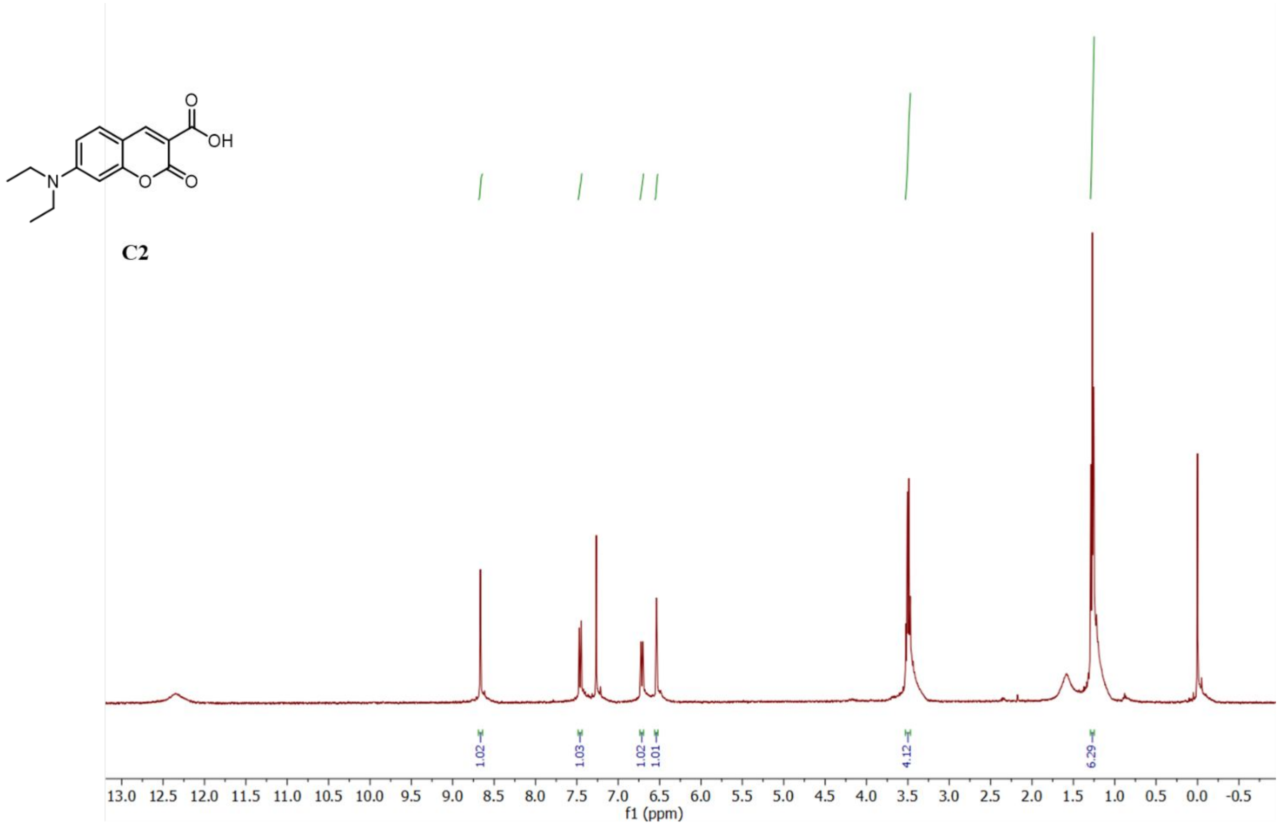

Figure S9. ^{1}H NMR of C2

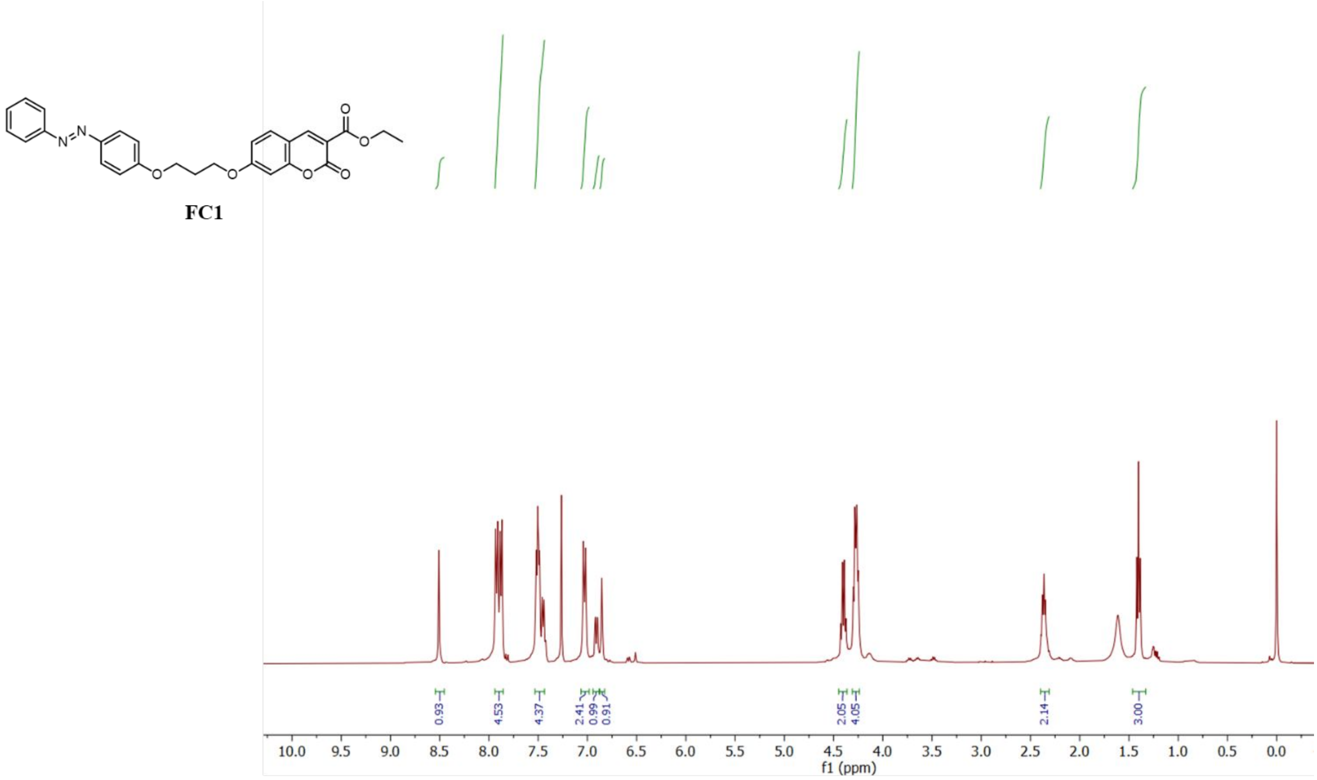

Figure S10. ^{1}H NMR of FC1

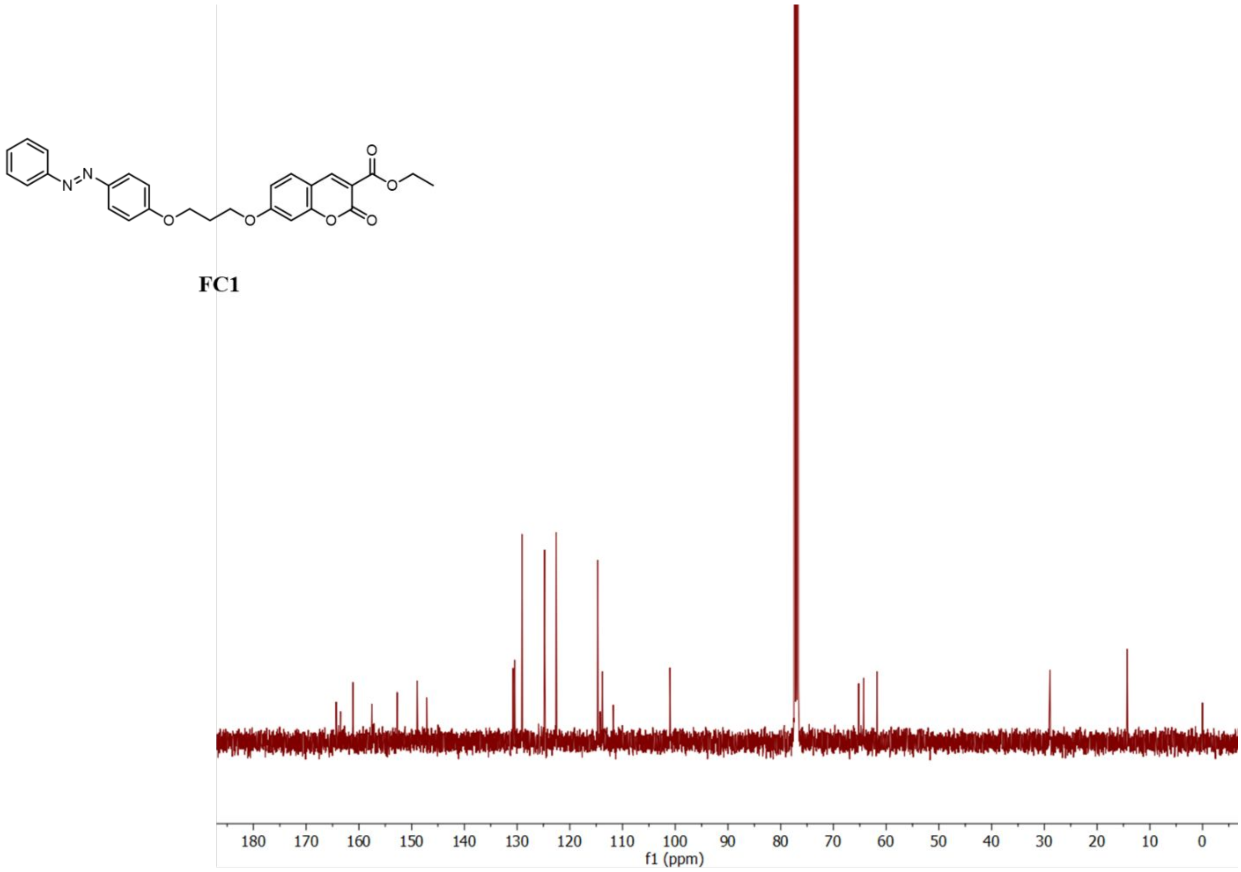

Figure S11. ^{13}C NMR of FC1

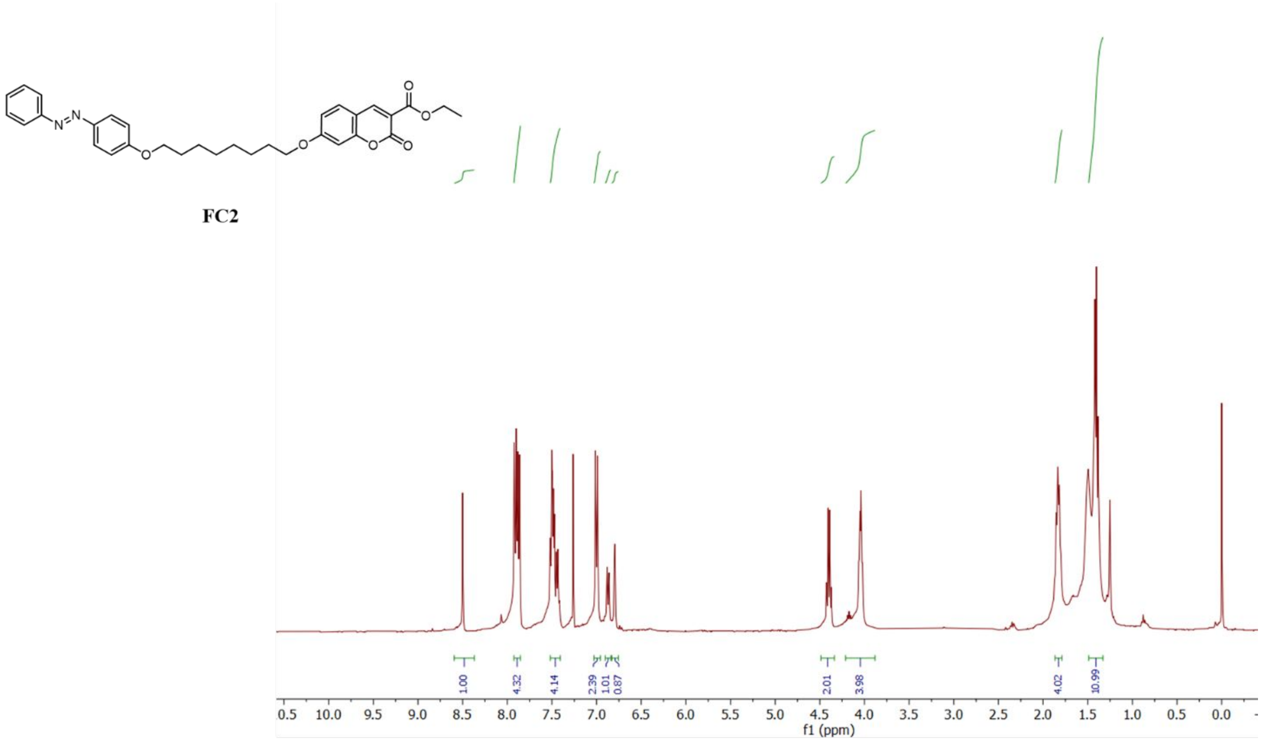

Figure S12. ^{1}H NMR of FC2

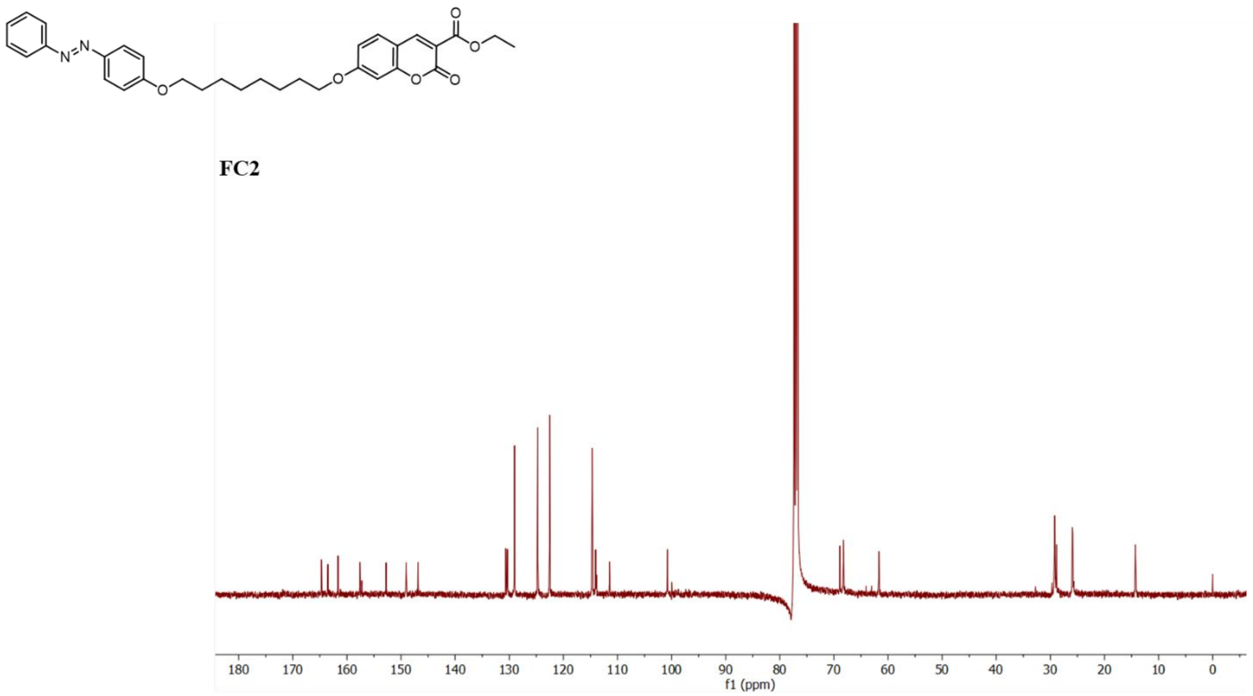

Figure S13. ^{13}**C NMR of FC2**

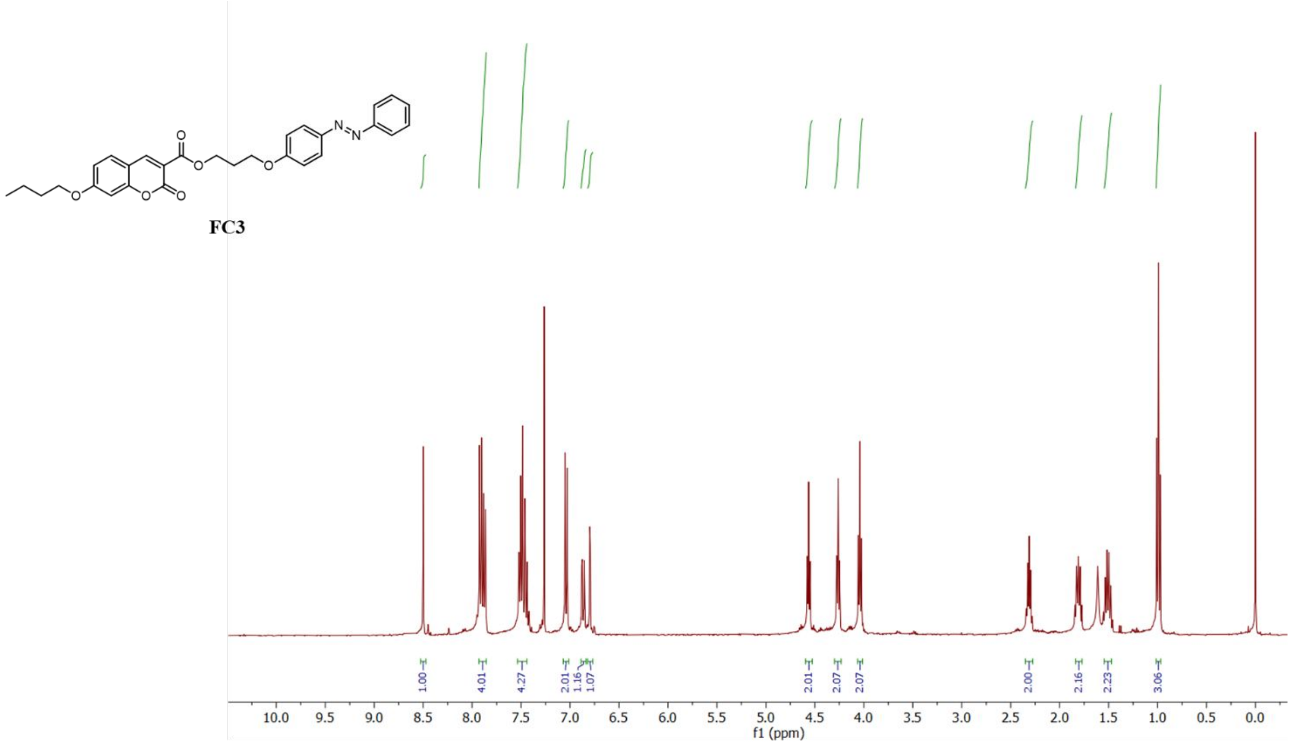

Figure S14. ^{1}**H NMR of FC3**

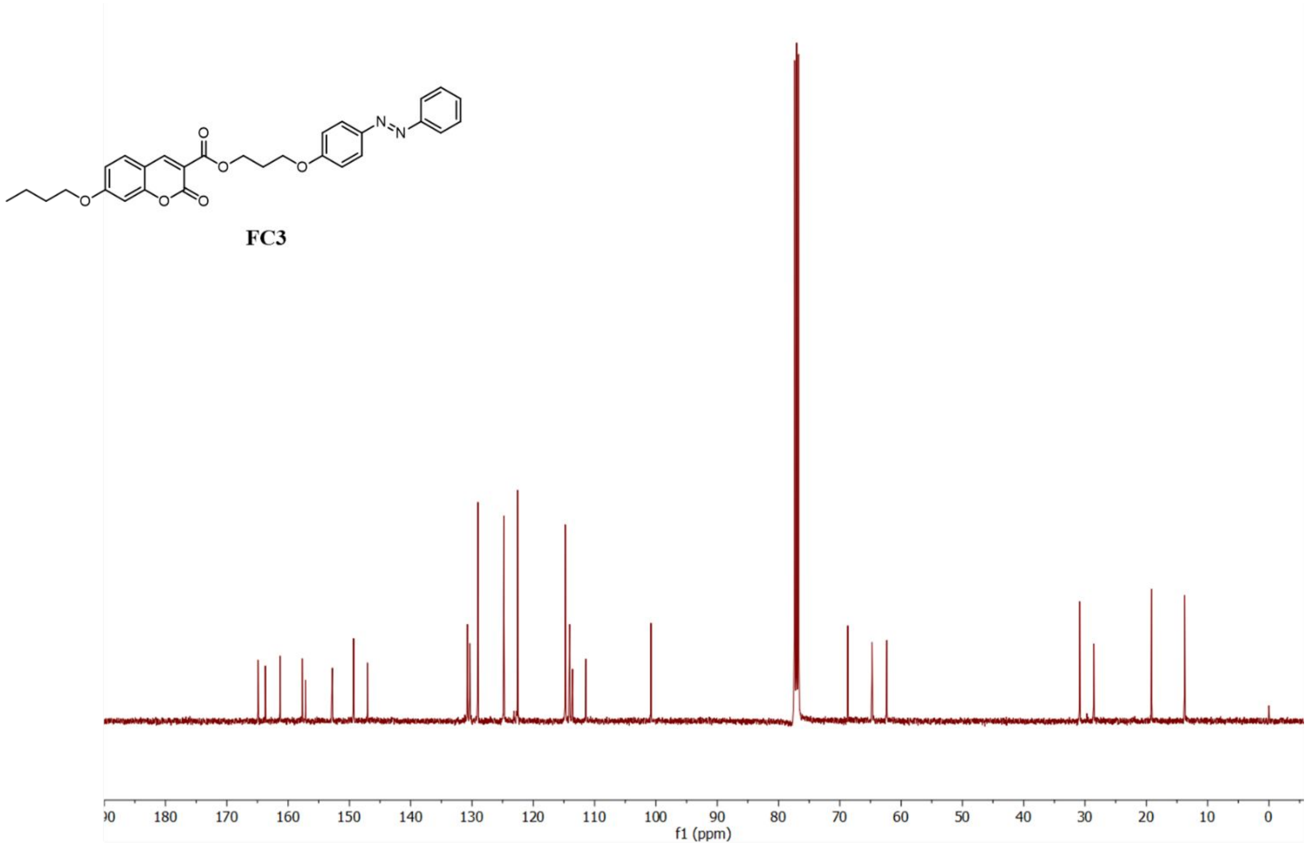

Figure S15. ^{13}**C NMR of FC3**

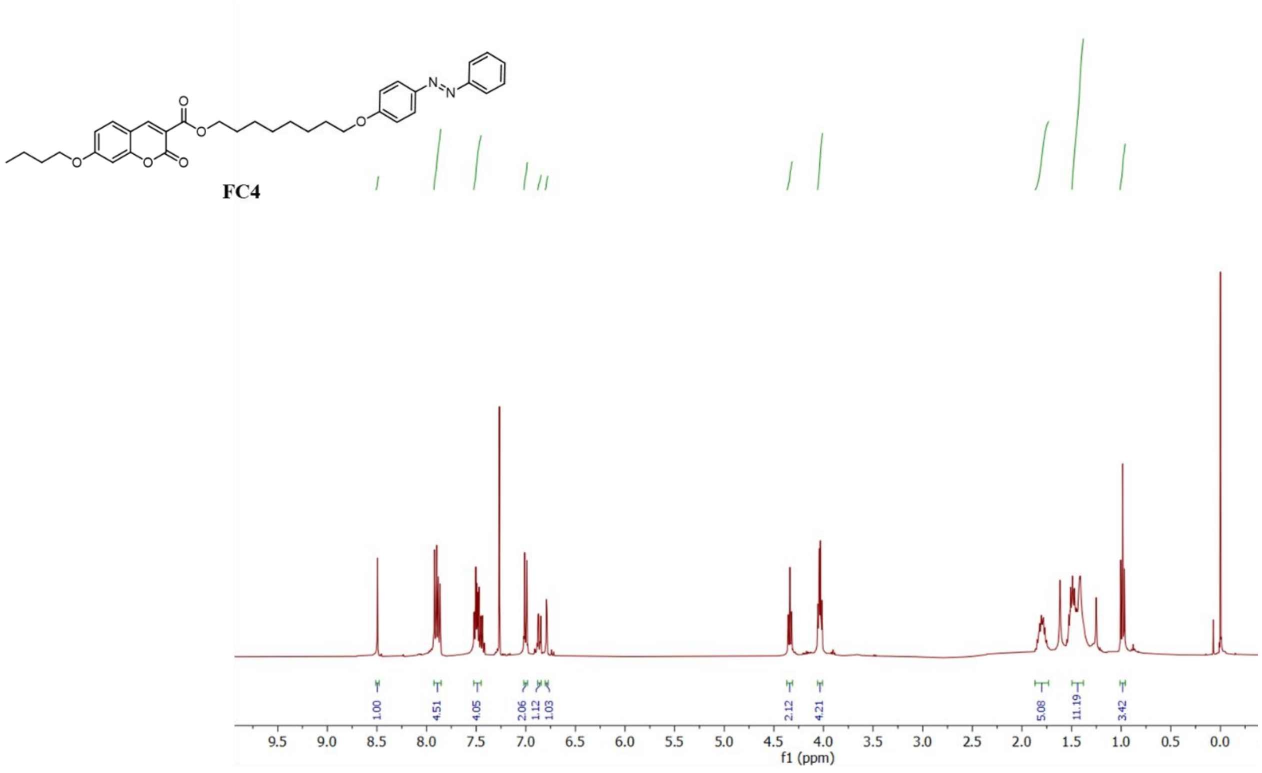

Figure S16. ^{1}**H NMR of FC4**

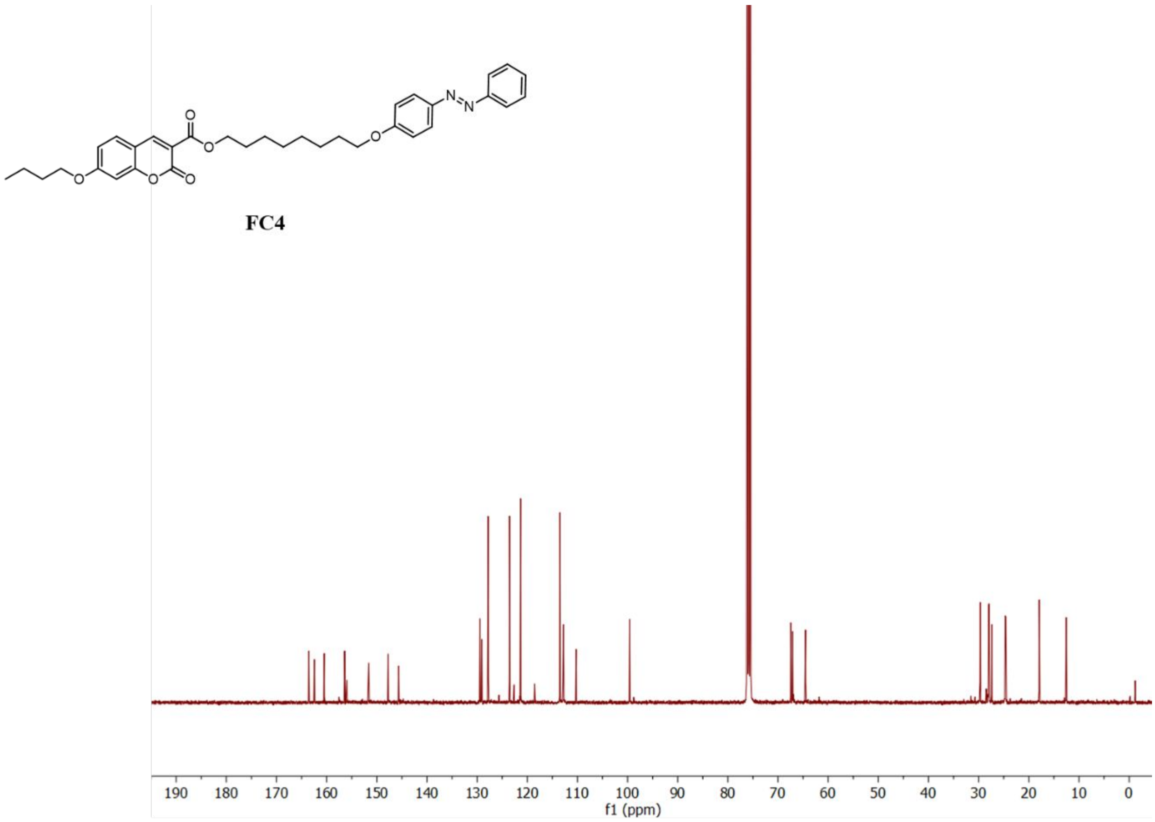

Figure S17. ^{13}C NMR of FC4

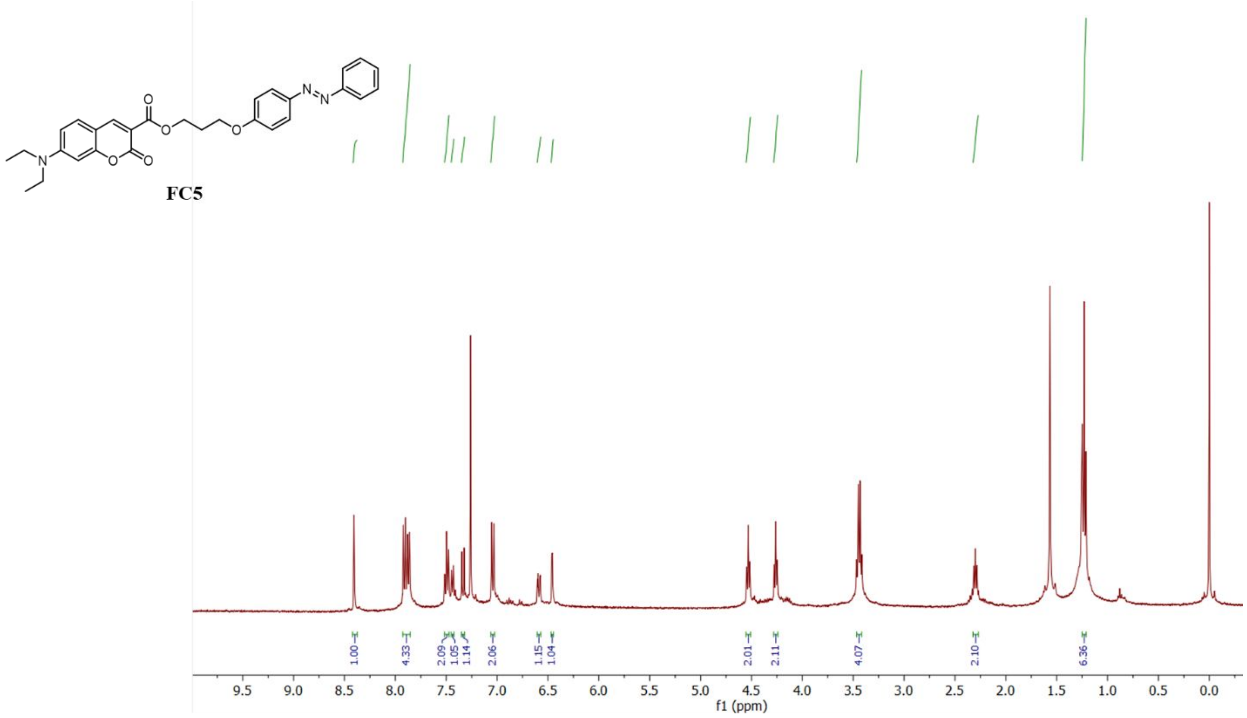

Figure S18. ^{1}H NMR of FC5

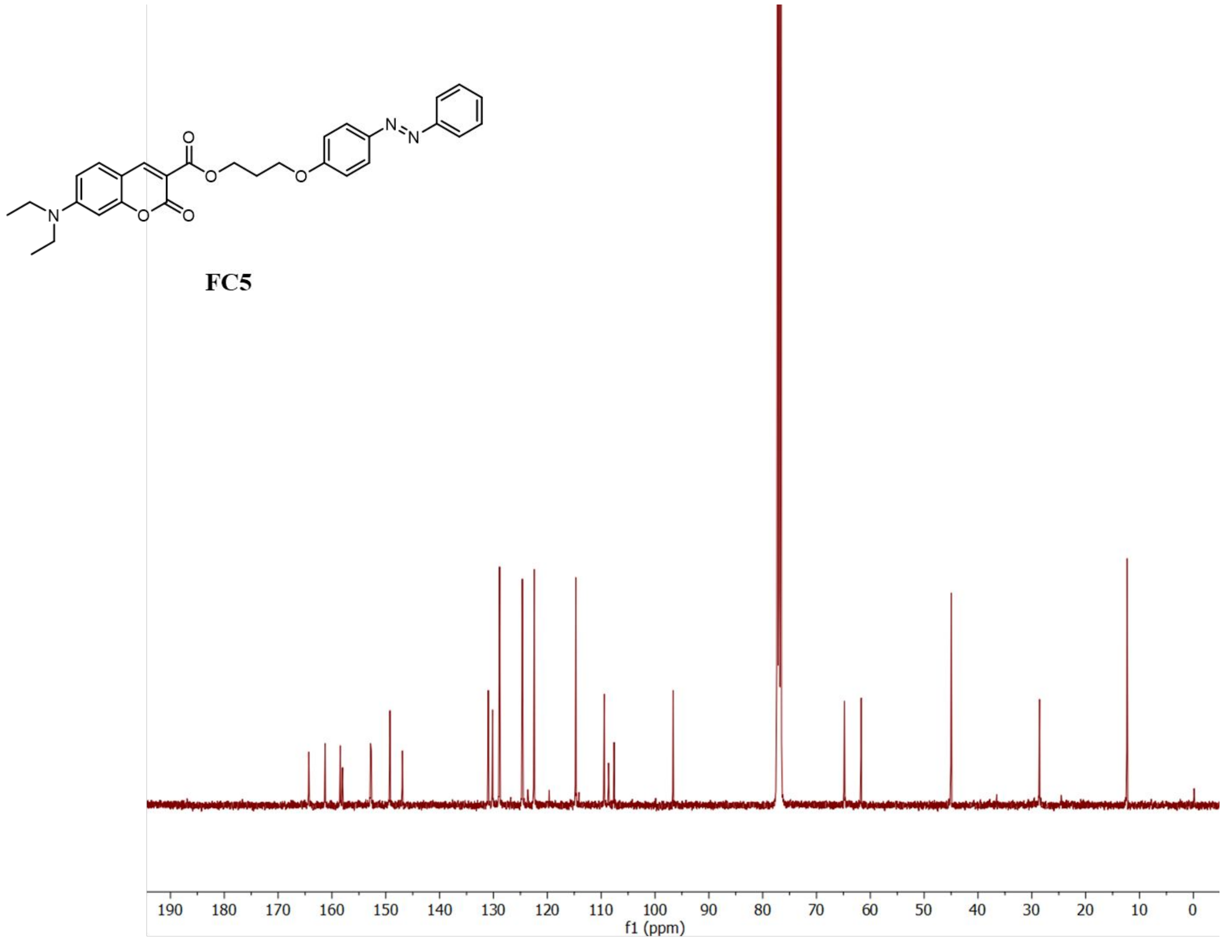

Figure S19. ^{13}C NMR of FC5

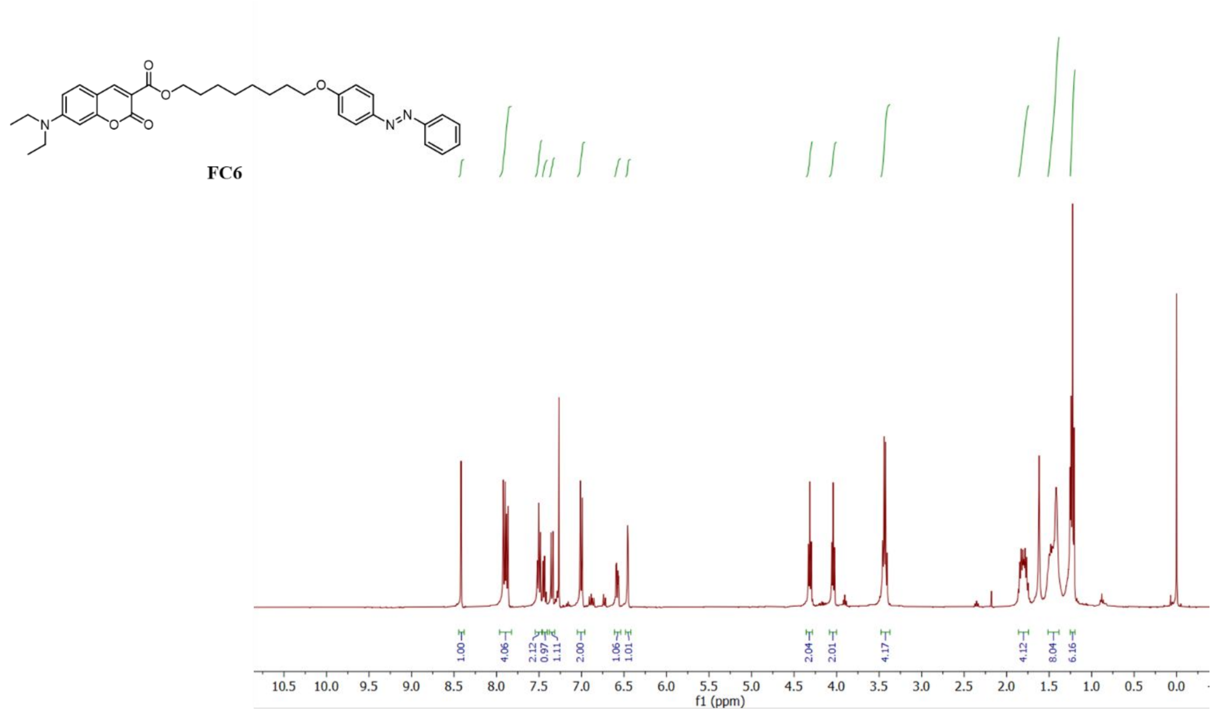

Figure S20. ^{1}H NMR of FC6

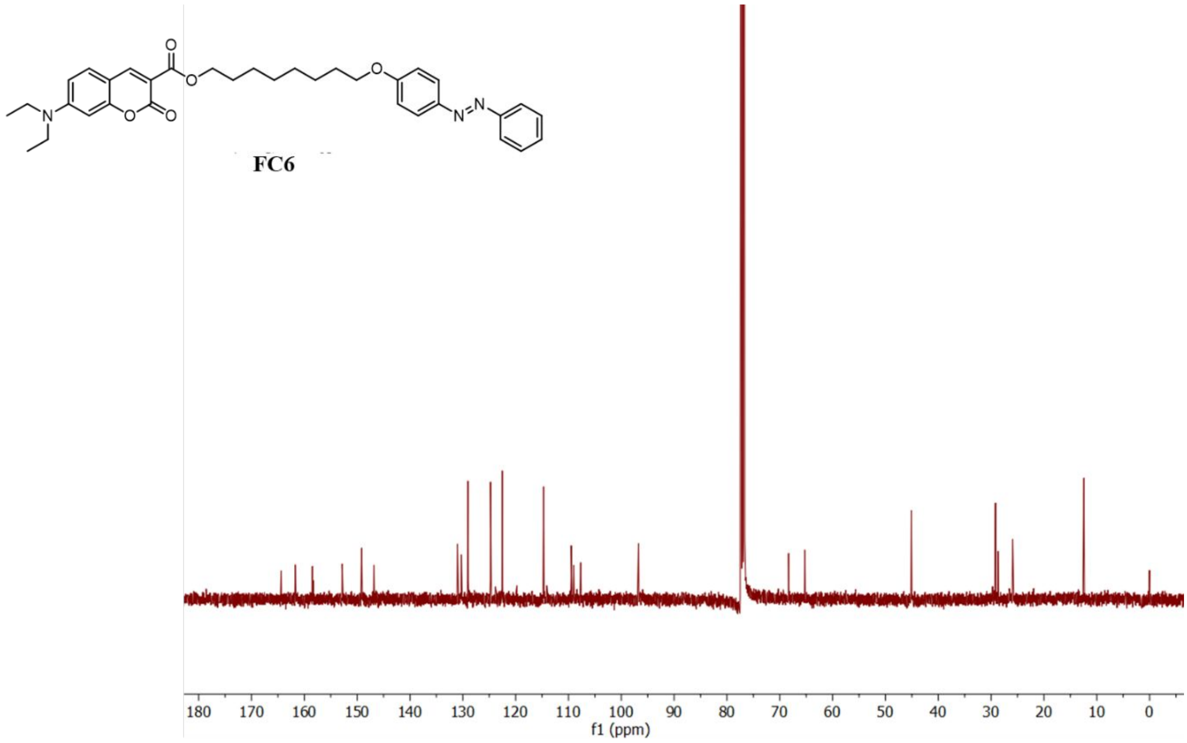

Figure S21. ^{13}C NMR of FC6

C. Infrared (IR) Spectra

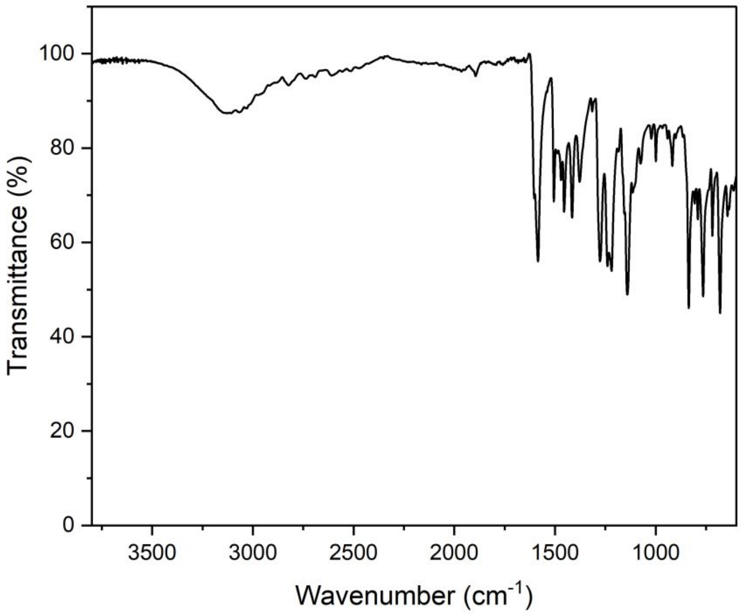

Figure S22. IR spectrum of 1

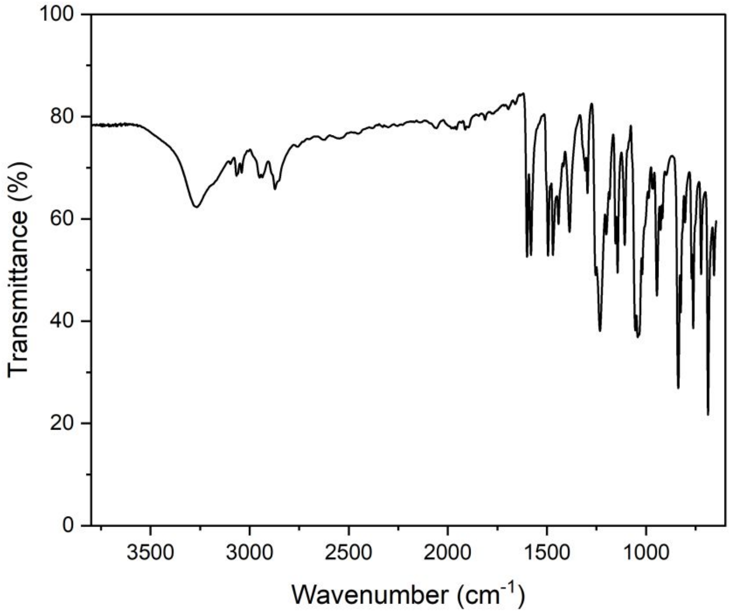

Figure S23. IR spectrum of 1a

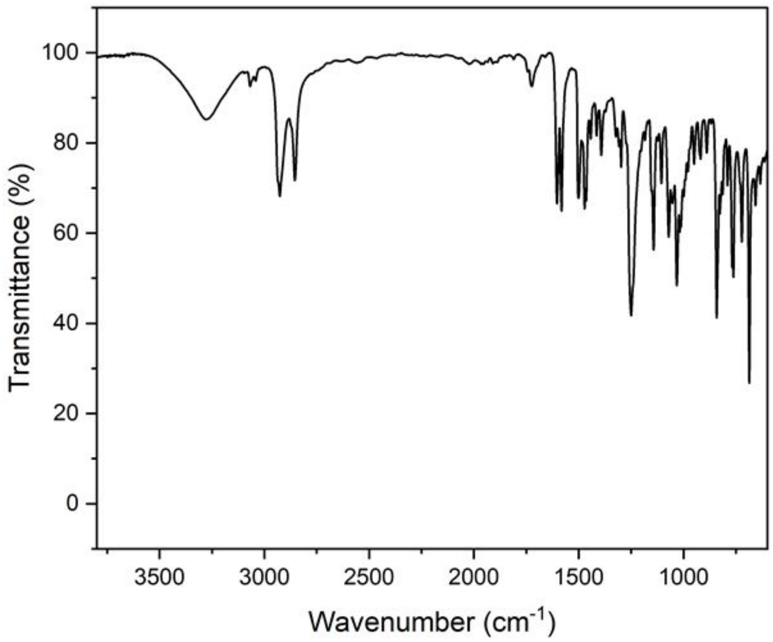

Figure S24. IR spectrum of 1b

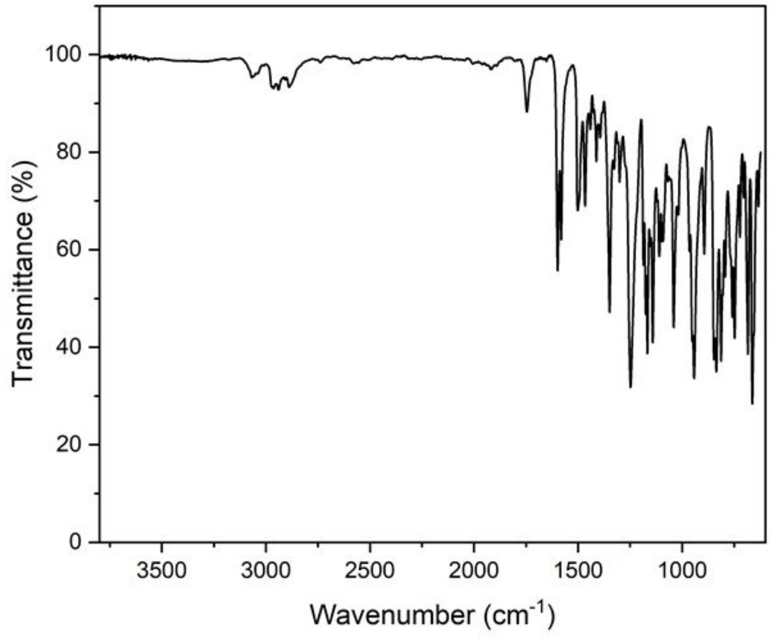

Figure S25. IR spectrum of 2a

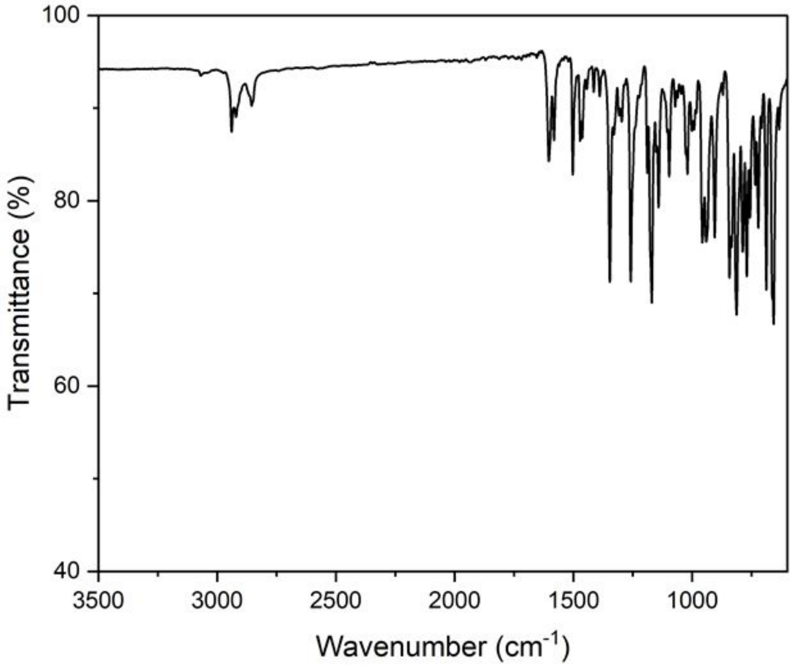

Figure S26. IR spectrum of 2b

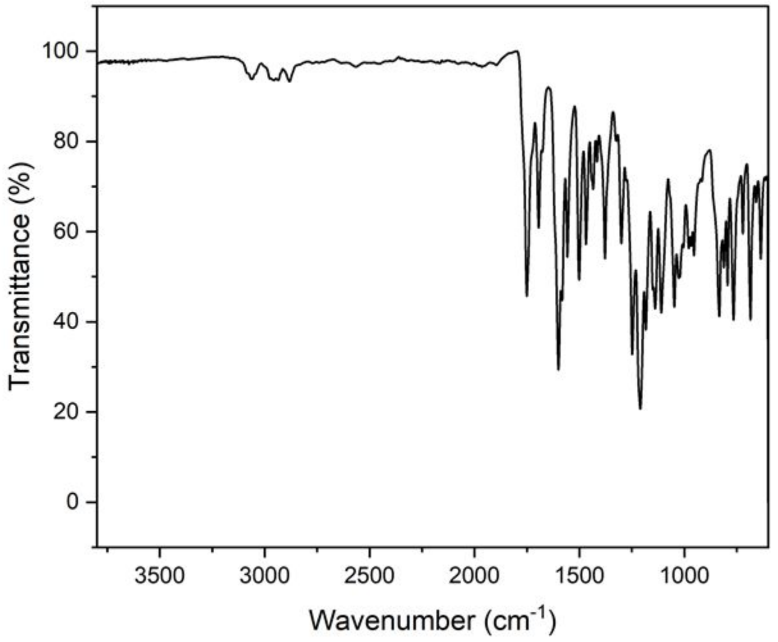

Figure S27. IR spectrum of FC1

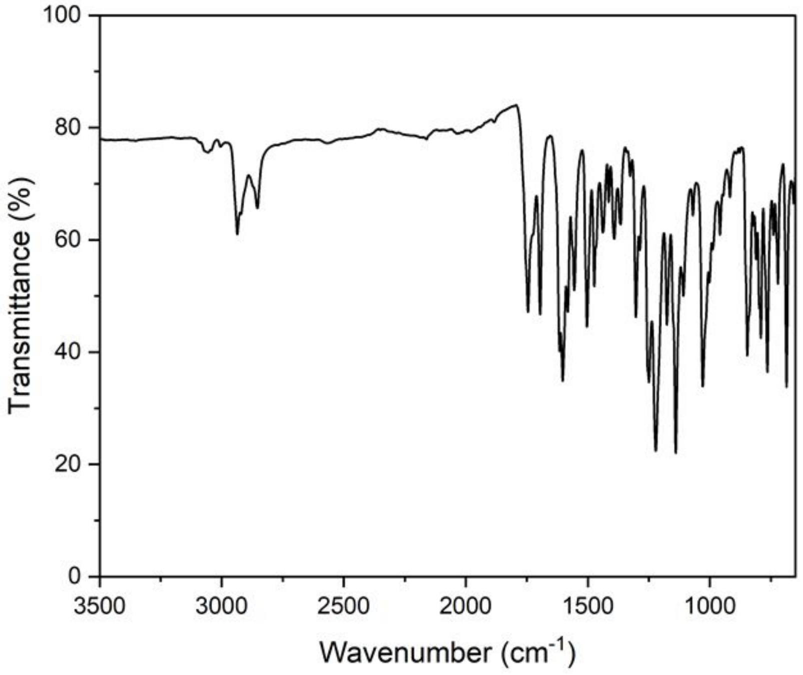

Figure S28. IR spectrum of FC2

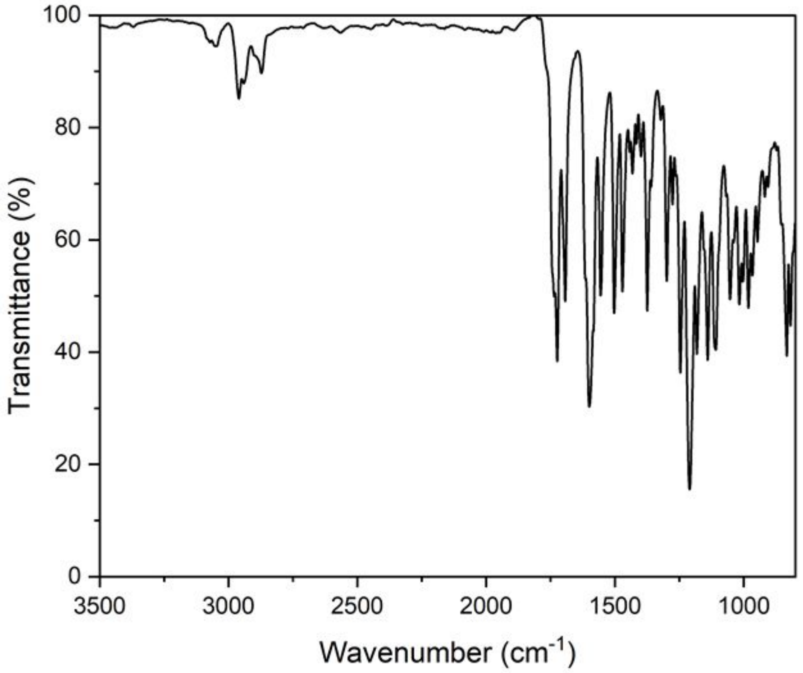

Figure S29. IR spectrum of FC3

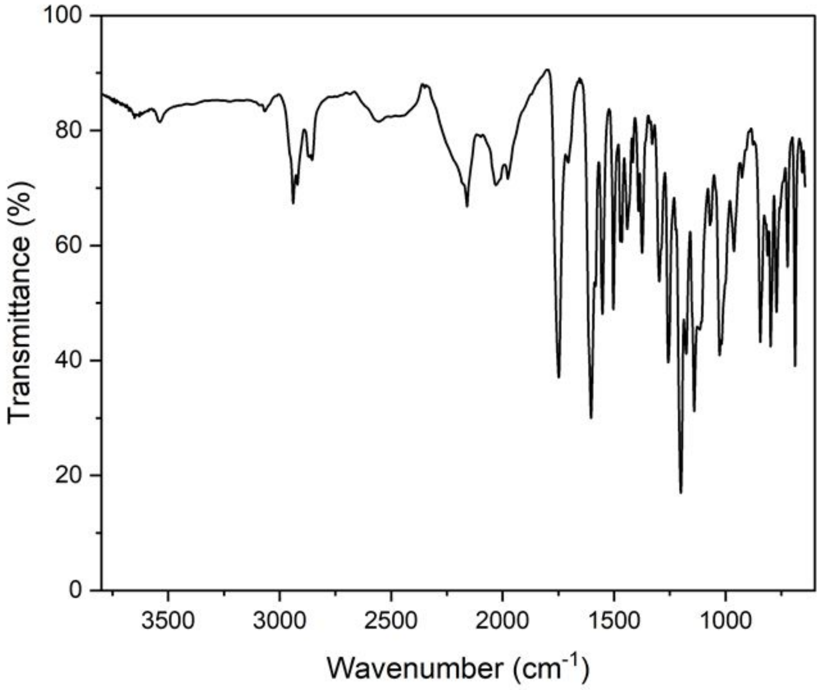

Figure S30. IR spectrum of FC4

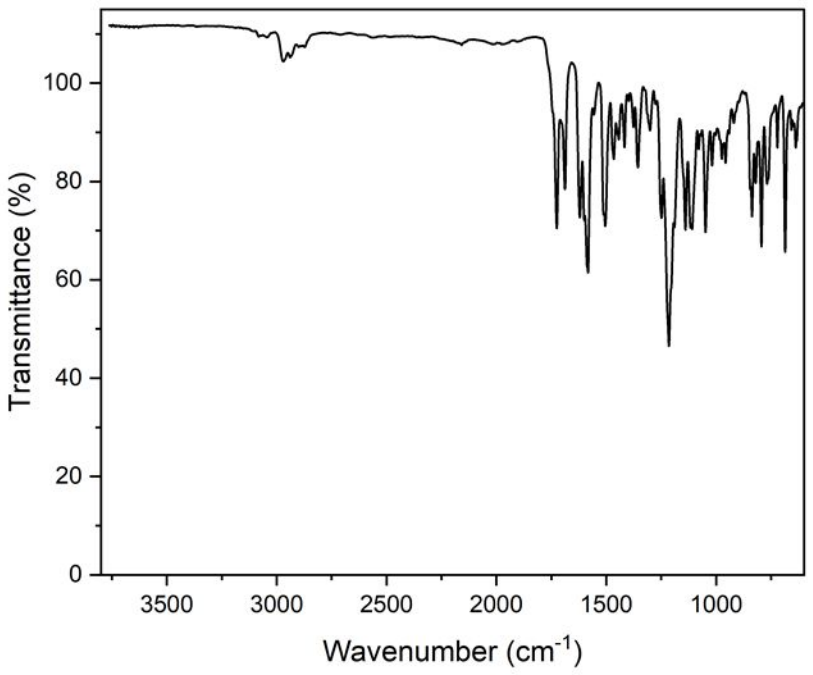

Figure S31. IR spectrum of FC5

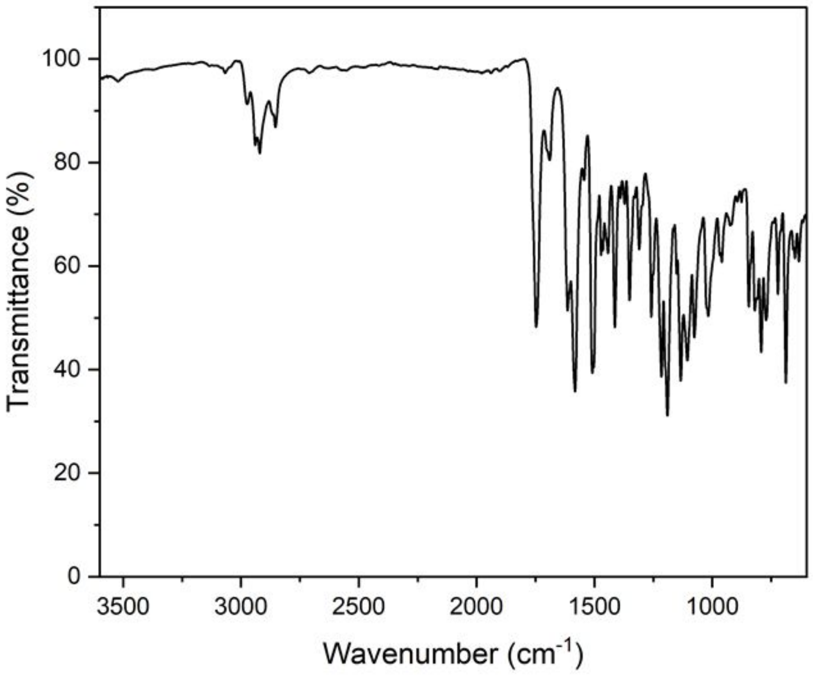

Figure S32. IR spectrum of FC6

D. High Resolution Mass Spectra (HRMS)

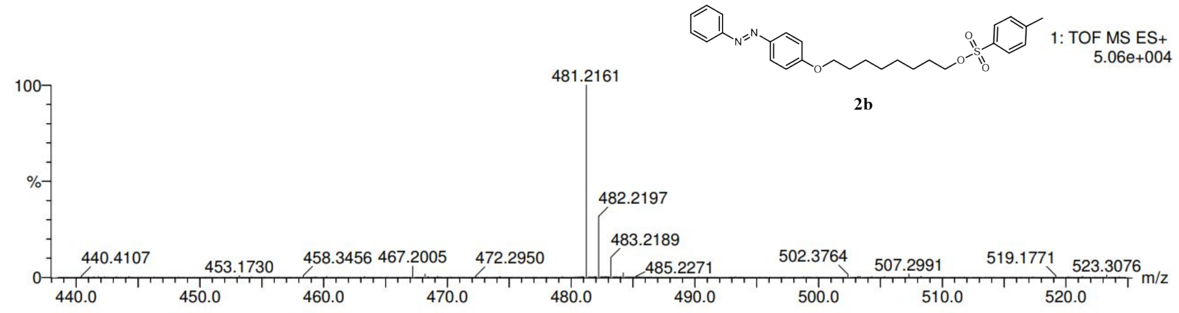

Minimum:				-1.5			
Maximum:		100.0	1000.0	50.0			
Mass	Calc. Mass	mDa	PPM	DBE	i-FIT	i-FIT (Norm)	Formula
481.2161	481.2161	0.0	0.0	12.5	379.0	0.0	C27 H33 N2 O4 S

Figure S33. HRMS spectrum of 2b

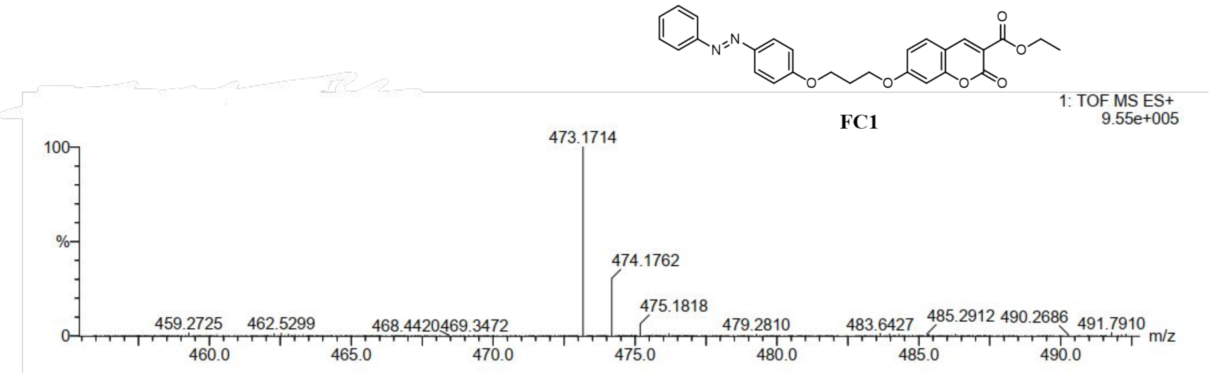

Minimum:				-5.5			
Maximum:		1000.0	1000.0	1000.0			
Mass	Calc. Mass	mDa	PPM	DBE	i-FIT	i-FIT (Norm)	Formula
473.1714	473.1713	0.1	0.2	16.5	1243.5	0.0	C27 H25 N2 O6

Figure S34. HRMS spectrum of FC1

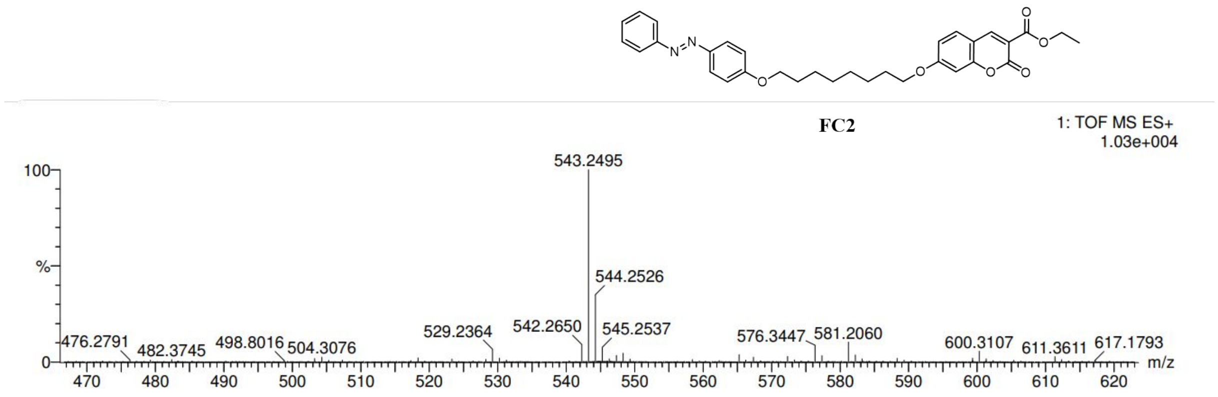

	Mass	Calc. Mass	mDa	PPM	DBE	i-FIT	i-FIT (Norm)	Formula
Minimum:					-1.5			
Maximum:			100.0	1000.0	50.0			
	543.2495	543.2495	0.0	0.0	16.5	199.3	0.0	C32 H35 N2 O6

Figure S35. HRMS spectrum of FC2

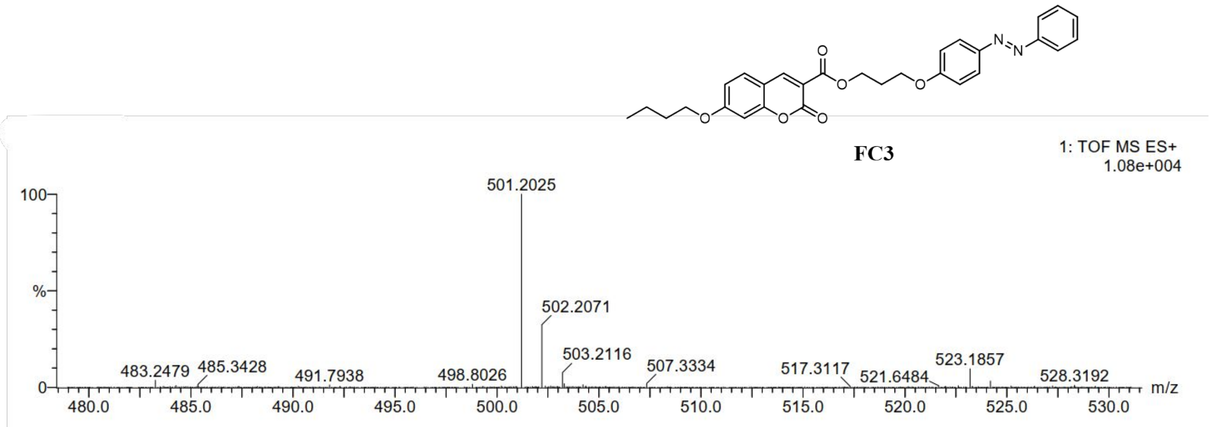

	Mass	Calc. Mass	mDa	PPM	DBE	i-FIT	i-FIT (Norm)	Formula
Minimum:					-5.5			
Maximum:			1000.0	1000.0	1000.0			
	501.2025	501.2026	-0.1	-0.2	16.5	609.5	0.0	C29 H29 N2 O6

Figure S36. HRMS spectrum of FC3

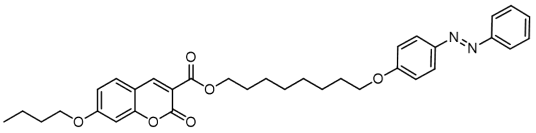

FC4

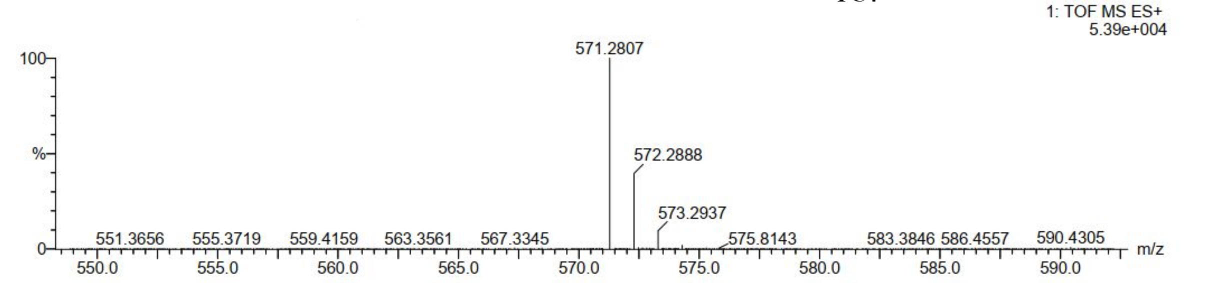

Minimum:				-5.5			
Maximum:		1000.0	1000.0	1000.0			
Mass	Calc. Mass	mDa	PPM	DBE	i-FIT	i-FIT (Norm)	Formula
571.2807	571.2808	-0.1	-0.2	16.5	789.6	0.0	C34 H39 N2 O6

Figure S37. HRMS spectrum of FC4

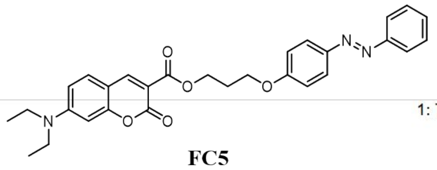

FC5

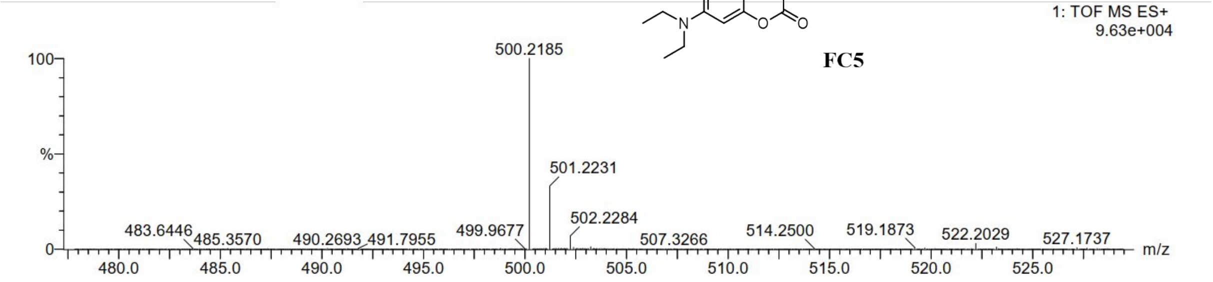

Minimum:				-5.5			
Maximum:		1000.0	1000.0	1000.0			
Mass	Calc. Mass	mDa	PPM	DBE	i-FIT	i-FIT (Norm)	Formula
500.2185	500.2185	0.0	0.0	16.5	1208.4	0.0	C29 H30 N3 O5

Figure S38. HRMS spectrum of FC5

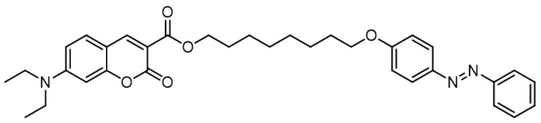

FC6

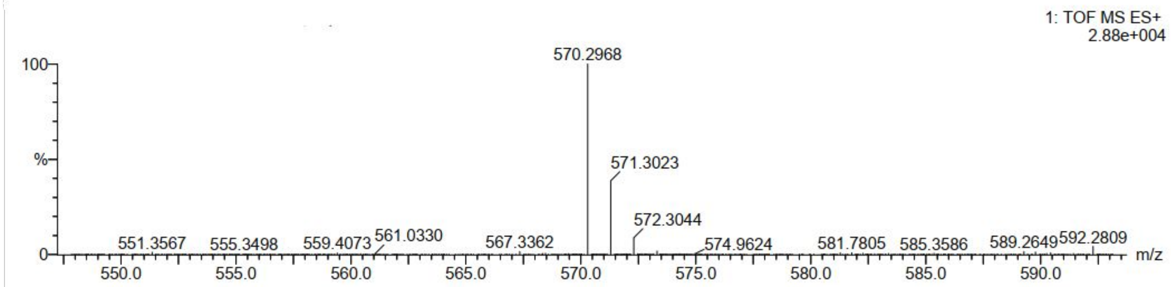

Minimum:				-5.5			
Maximum:		1000.0	1000.0	1000.0			
Mass	Calc. Mass	mDa	PPM	DBE	i-FIT	i-FIT (Norm)	Formula
570.2968	570.2968	0.0	0.0	16.5	785.4	0.0	C34 H40 N3 O5

Figure S39. HRMS spectrum of FC6

E. UV-Vis Spectra

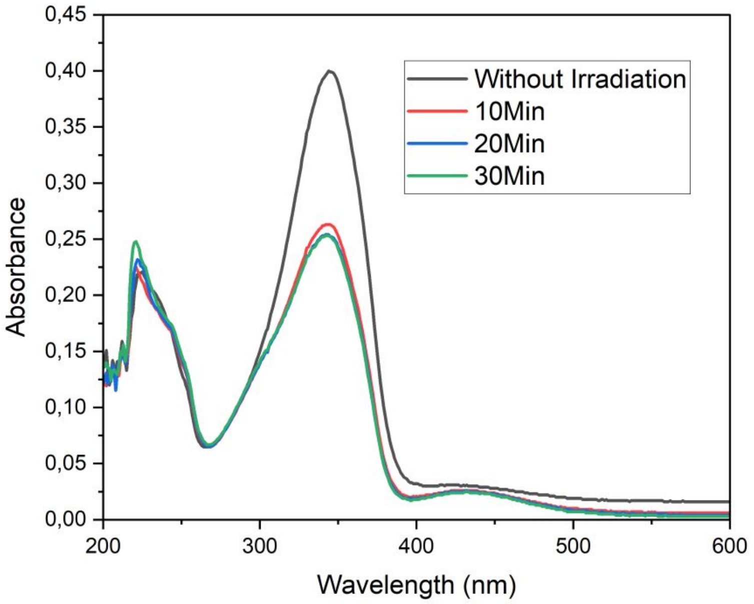

Figure S40. UV-Vis Spectra of FC1 upon irradiation in MeCN (10^{-5} M)

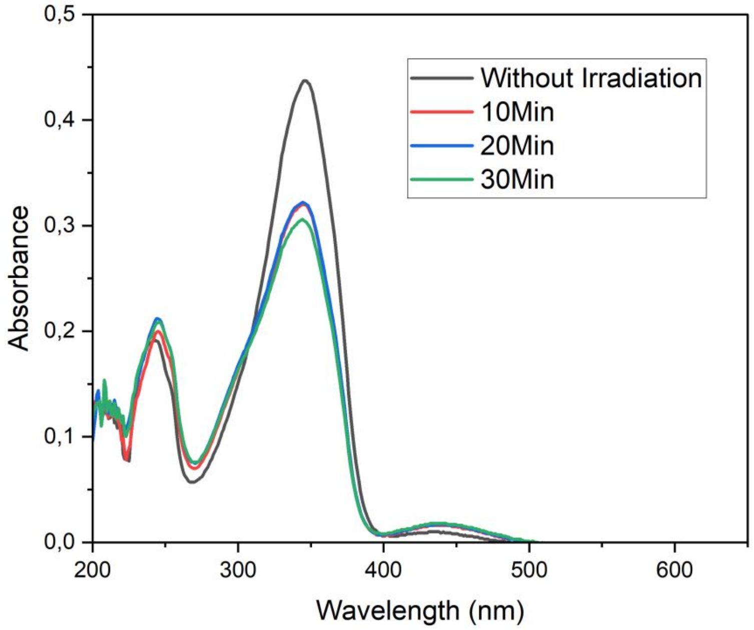

Figure S41. UV-Vis Spectra of FC1 upon irradiation in THF (10^{-5} M)

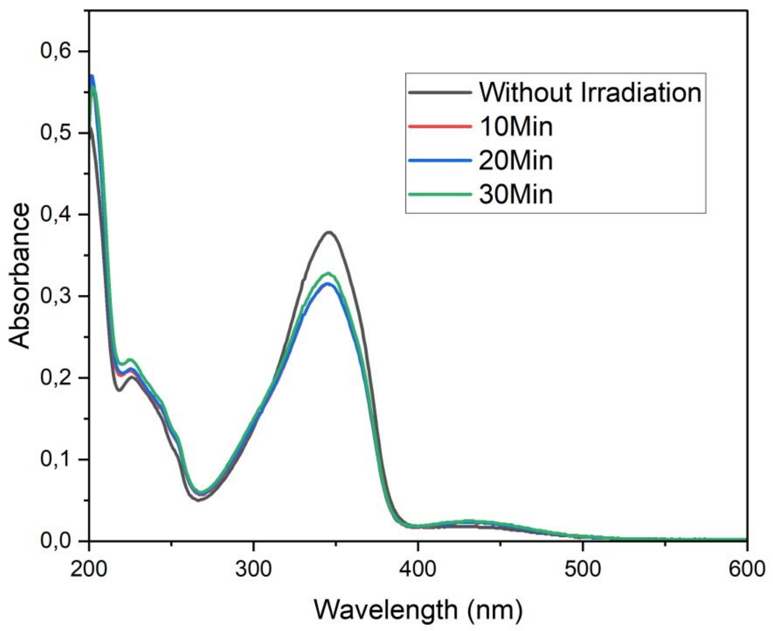

Figure S42. UV-Vis Spectra of FC1 upon irradiation in MeOH (10^{-5} M)

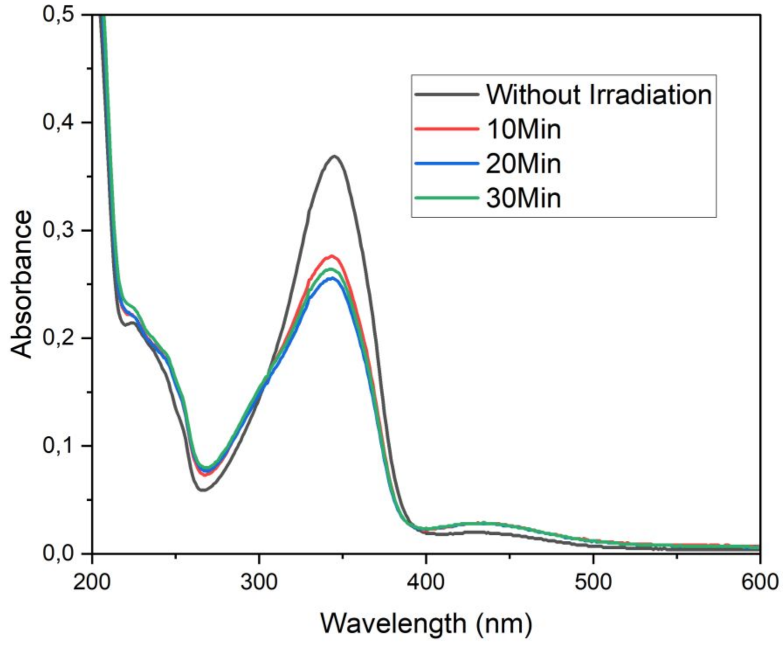

Figure S43. UV-Vis Spectra of FC2 upon irradiation in MeCN (10^{-5} M)

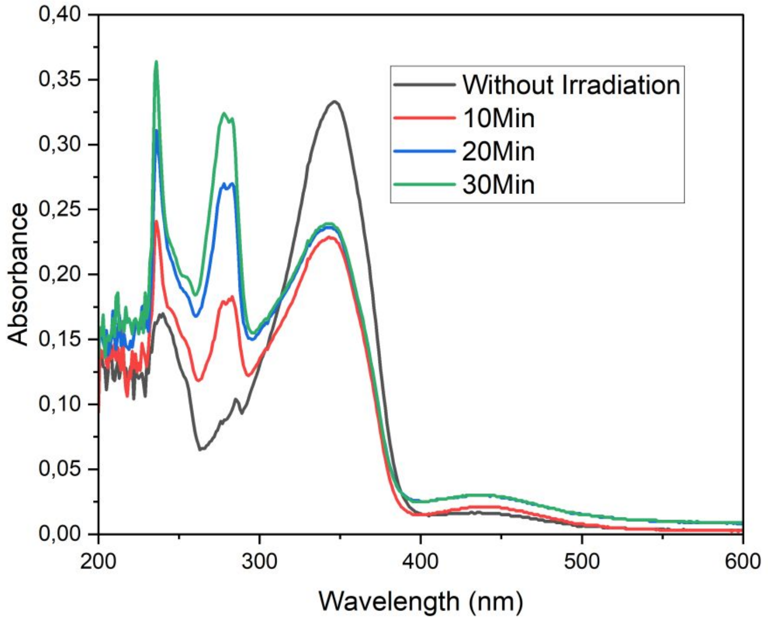

Figure S44. UV-Vis Spectra of FC2 upon irradiation in THF (10^{-5} M)

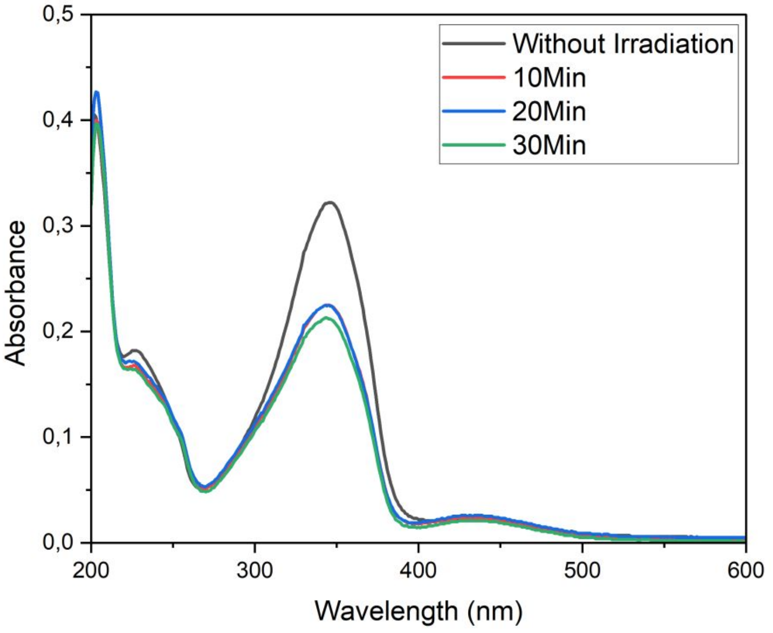

Figure S45. UV-Vis Spectra of FC2 upon irradiation in MeOH (10^{-5} M)

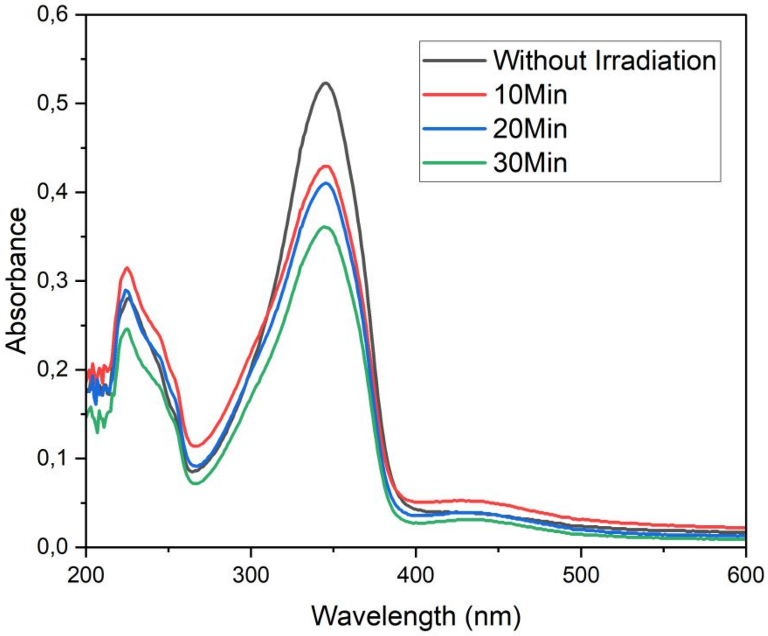

Figure S46. UV-Vis Spectra of FC3 upon irradiation in MeCN (10^{-5} M)

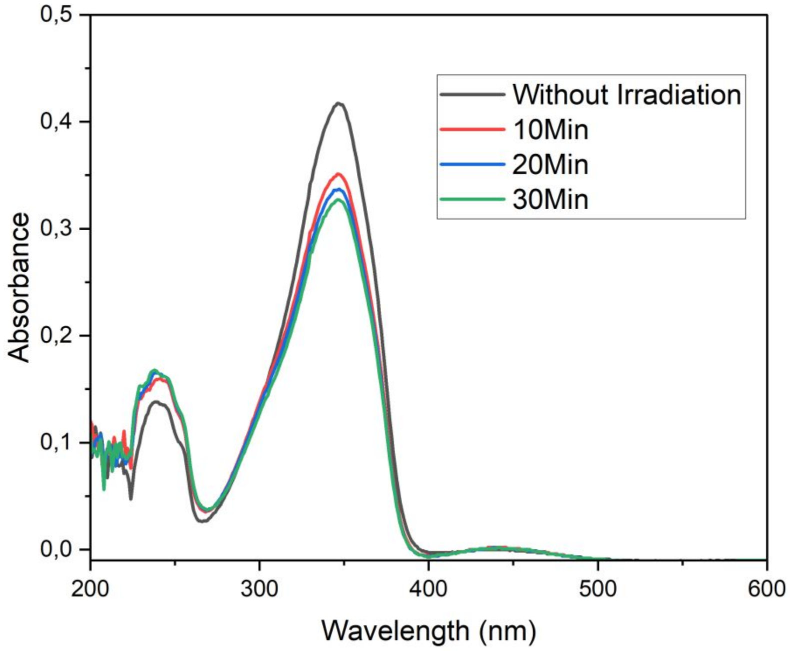

Figure S47. UV-Vis Spectra of FC3 upon irradiation in THF (10^{-5} M)

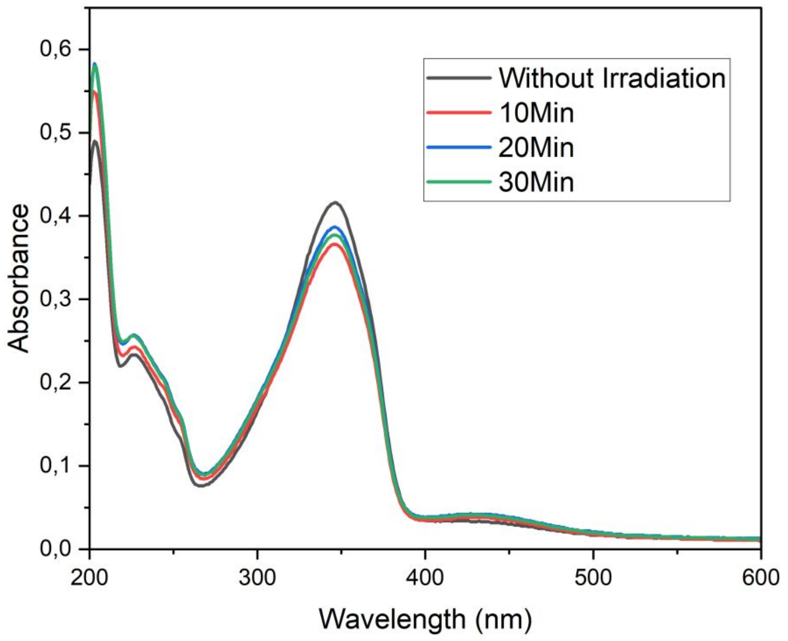

Figure S48. UV-Vis Spectra of FC3 upon irradiation in MeOH (10^{-5} M)

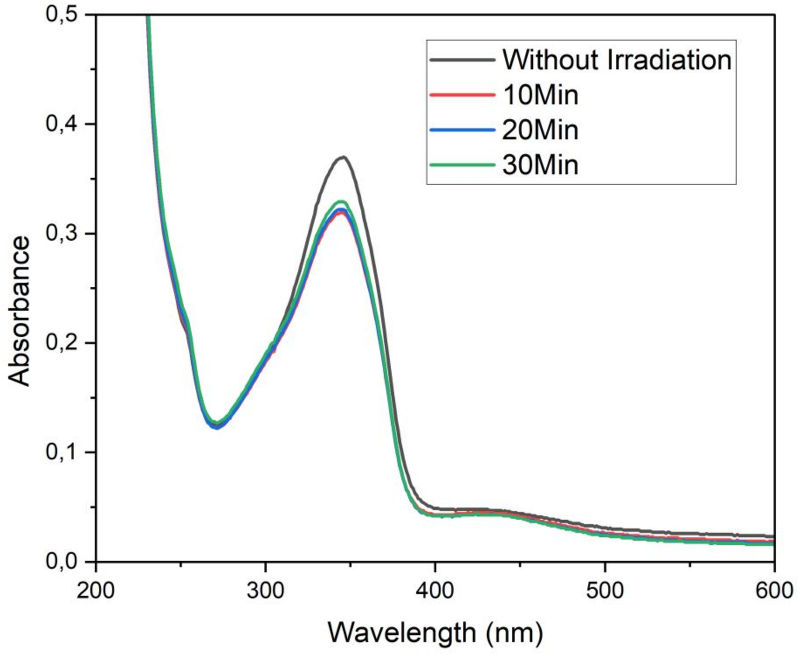

Figure S49. UV-Vis Spectra of FC4 upon irradiation in MeCN (10^{-5} M)

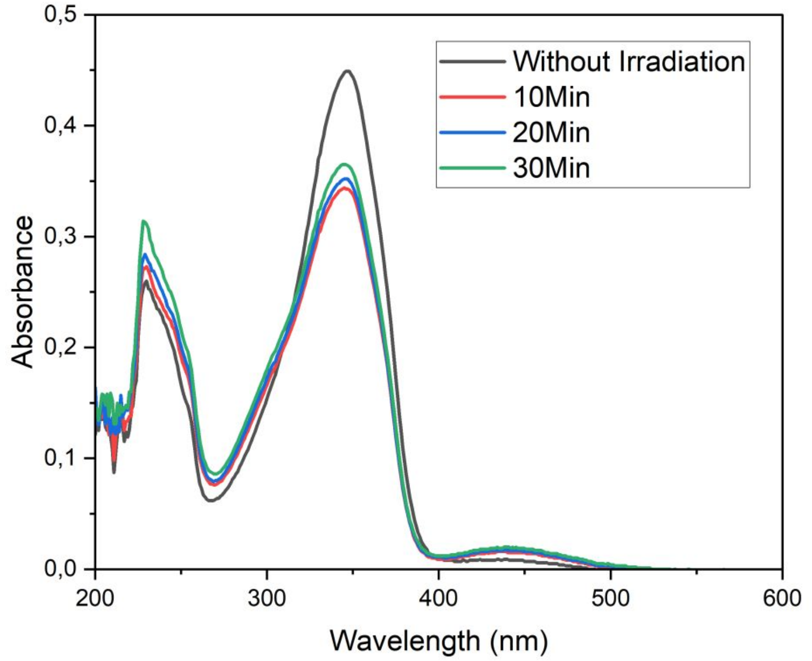

Figure S50. UV-Vis Spectra of FC4 upon irradiation in THF (10^{-5} M)

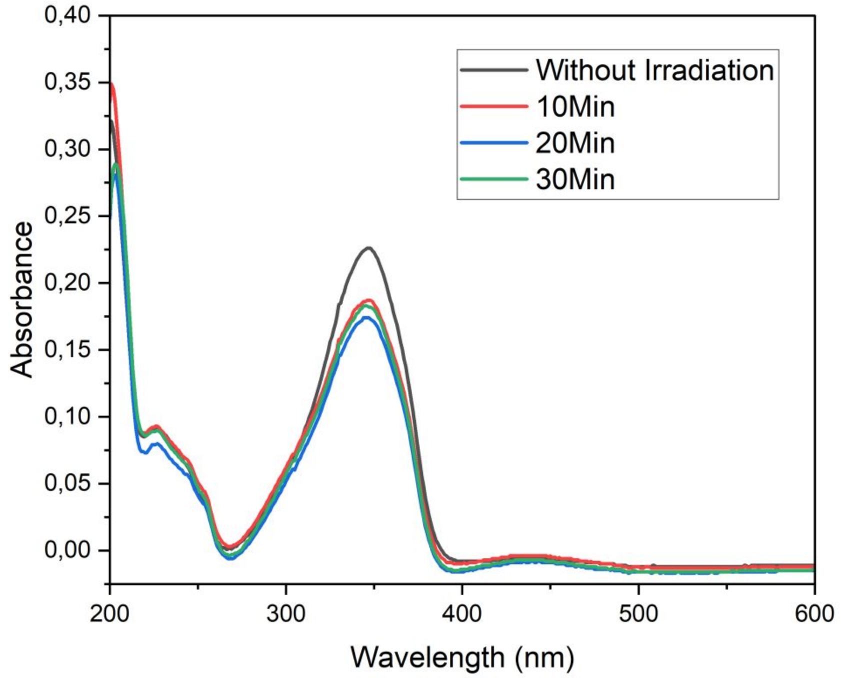

Figure S51. UV-Vis Spectra of FC4 upon irradiation in MeOH (10^{-5} M)

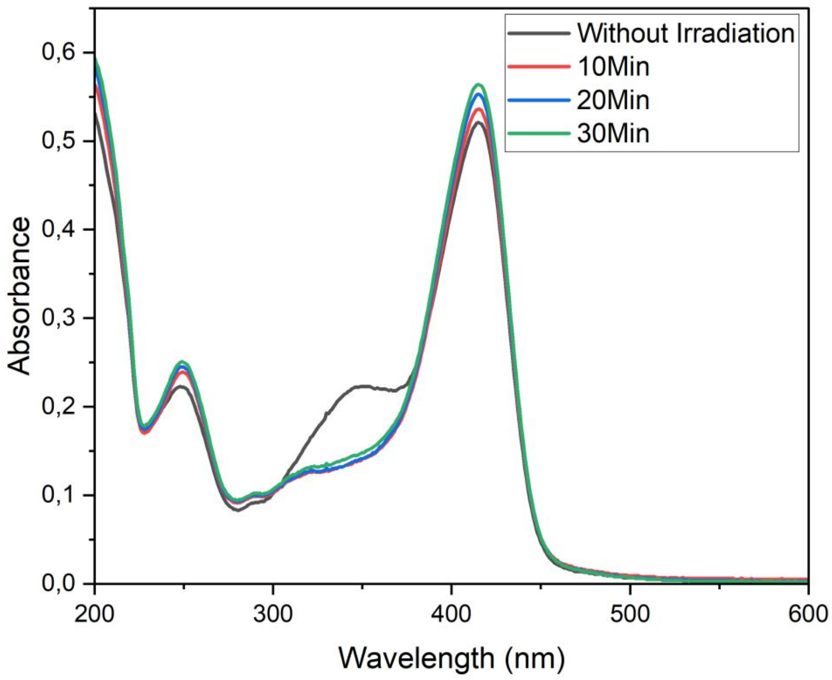

Figure S52. UV-Vis Spectra of FC5 upon irradiation in MeCN (10^{-5} M)

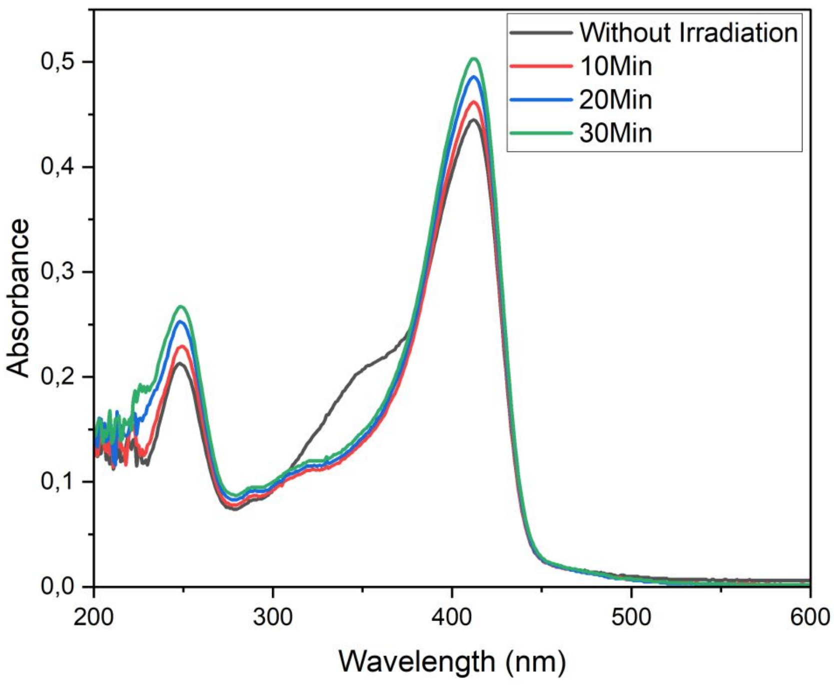

Figure S53. UV-Vis Spectra of FC5 upon irradiation in THF (10^{-5} M)

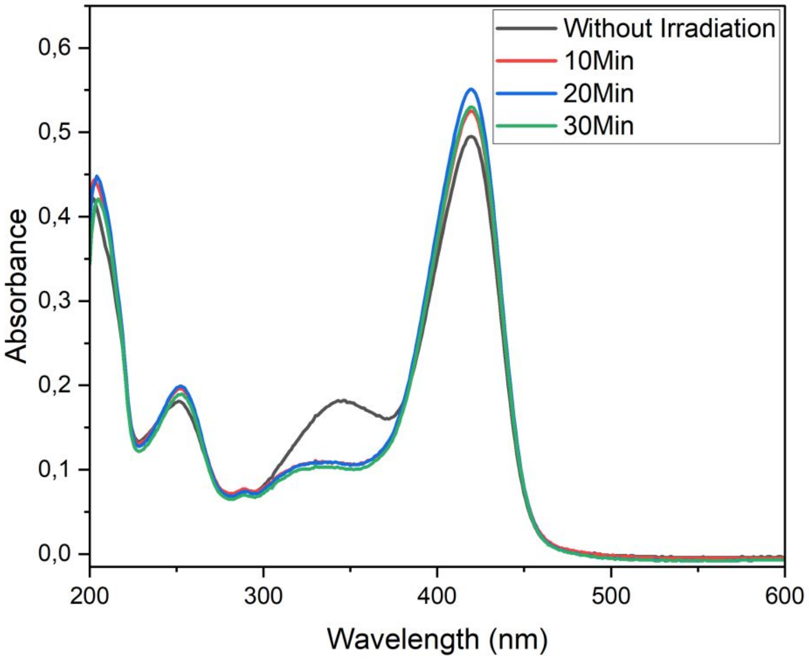

Figure S54. UV-Vis Spectra of FC5 upon irradiation in MeOH (10^{-5} M)

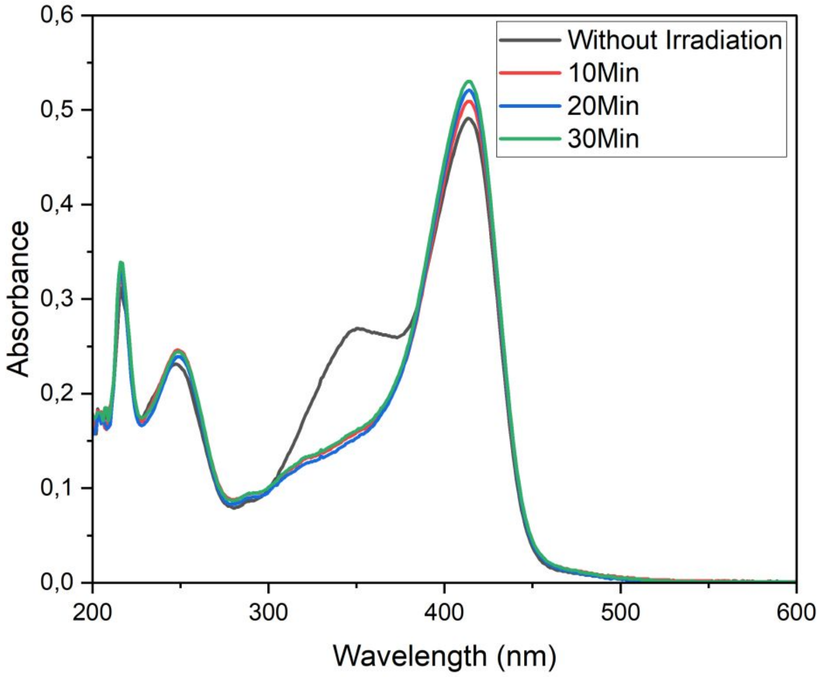

Figure S55. UV-Vis Spectra of FC6 upon irradiation in MeCN (10^{-5} M)

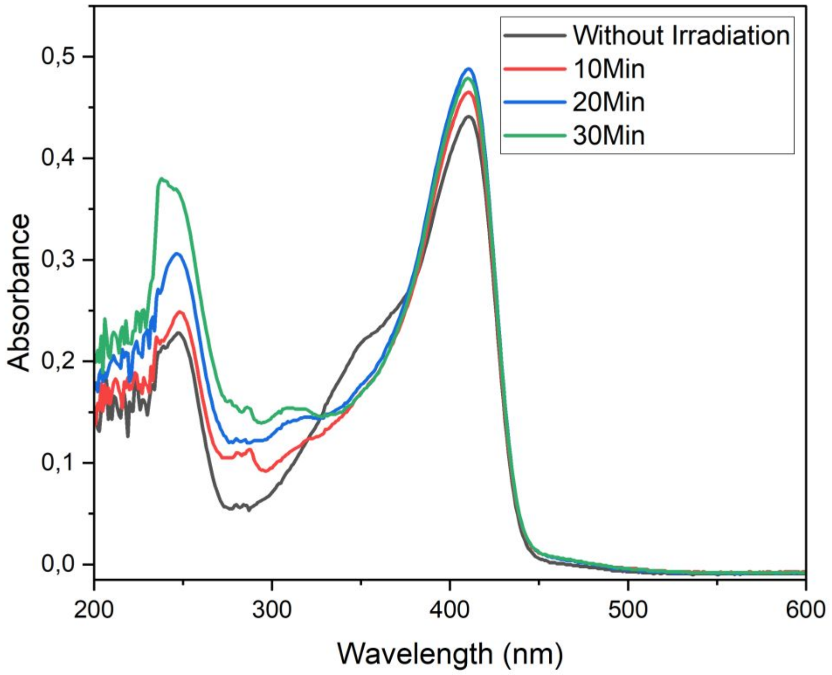

Figure S56. UV-Vis Spectra of FC6 upon irradiation in THF (10^{-5} M)

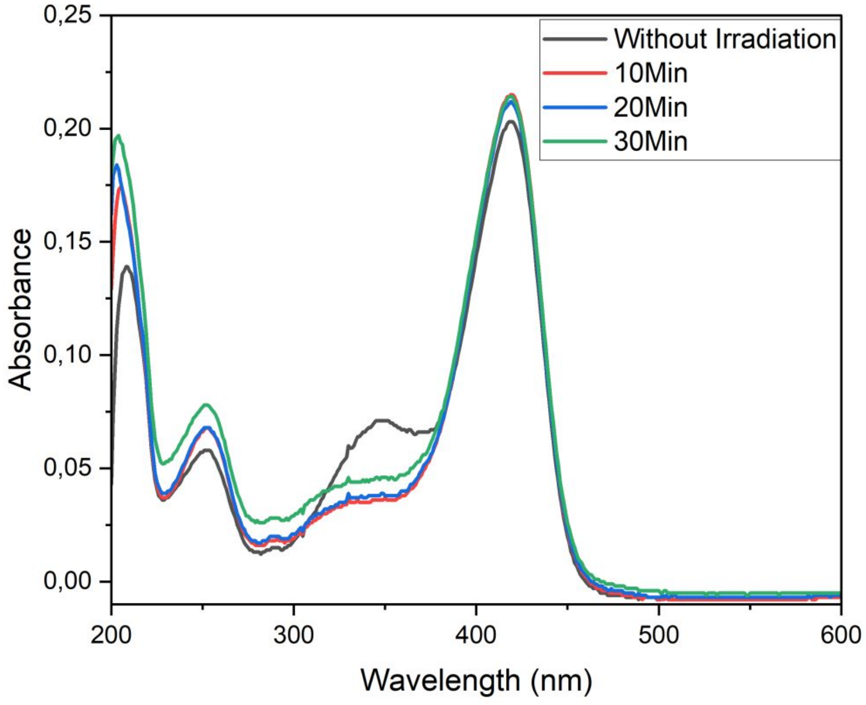

Figure S57. UV-Vis Spectra of FC6 upon irradiation in MeOH (10^{-5} M)

F. Fluorescence Spectra

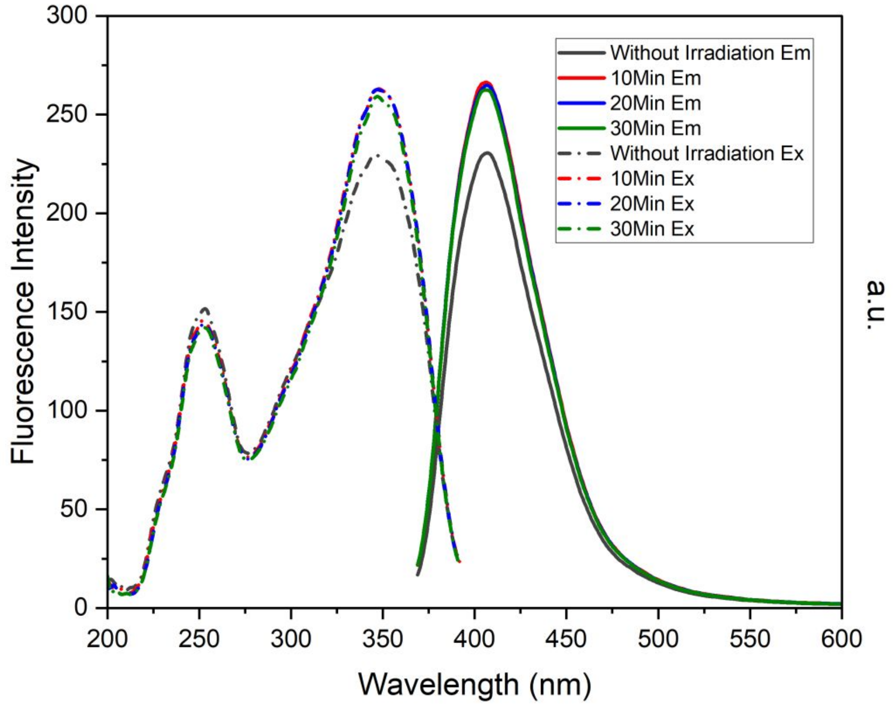

Figure S58. Fluorescence Spectra of FC1 upon irradiation in MeCN (10^{-5} M)

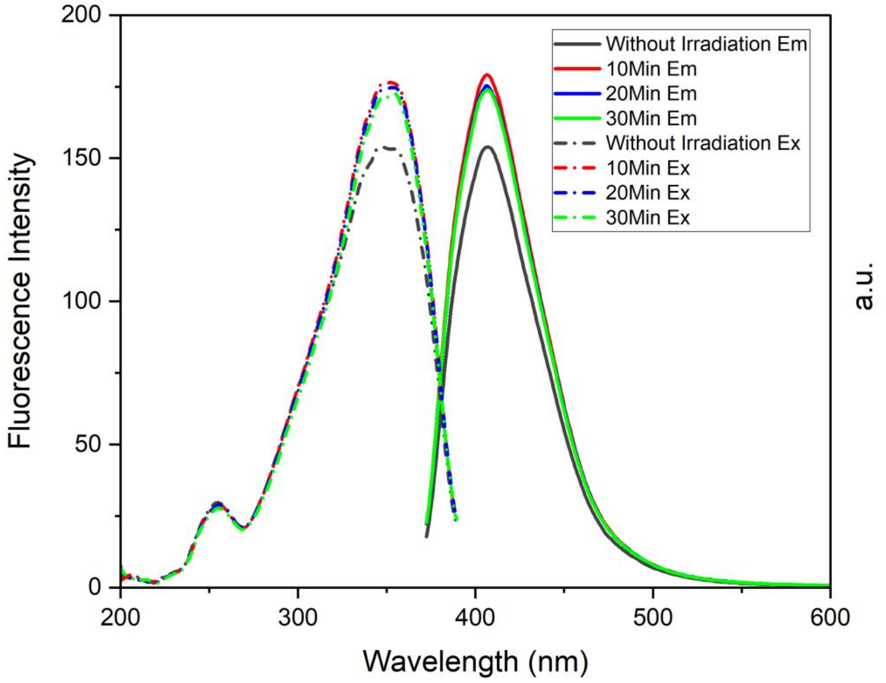

Figure S59. Fluorescence Spectra of FC1 upon irradiation in THF (10^{-5} M)

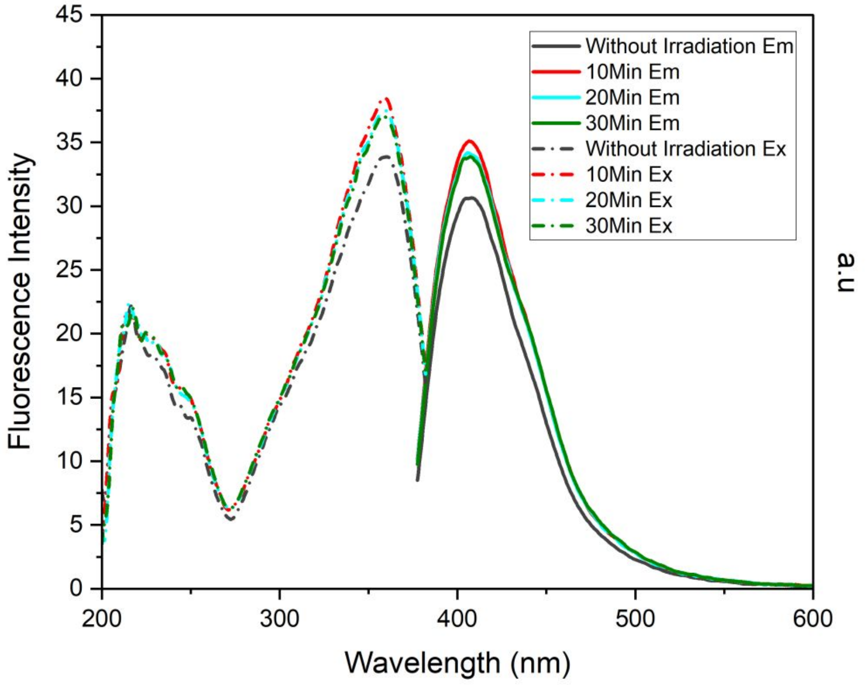

Figure S60. Fluorescence Spectra of FC1 upon irradiation in MeOH (10^{-5} M)

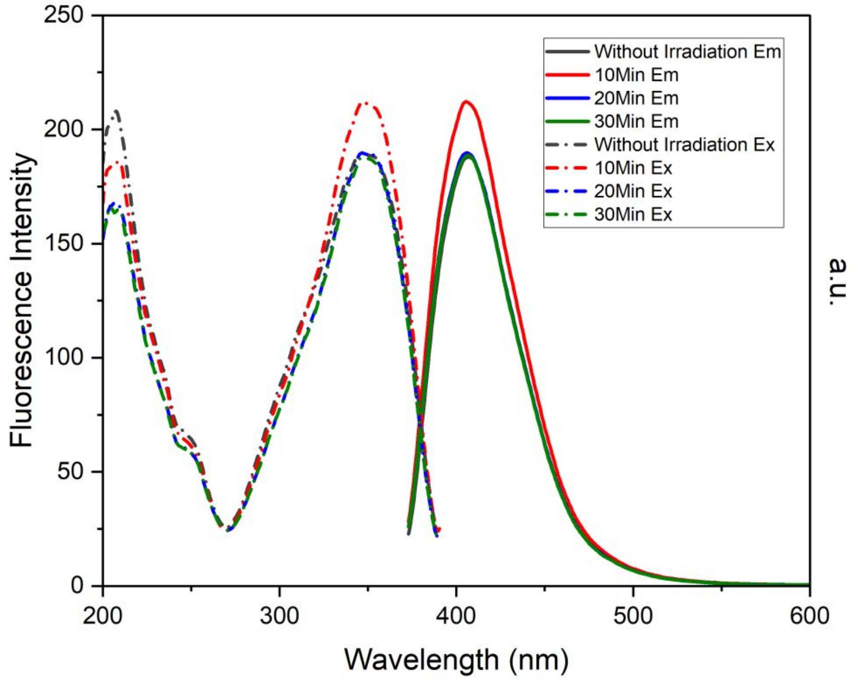

Figure S61. Fluorescence Spectra of FC2 upon irradiation in MeCN (10^{-5} M)

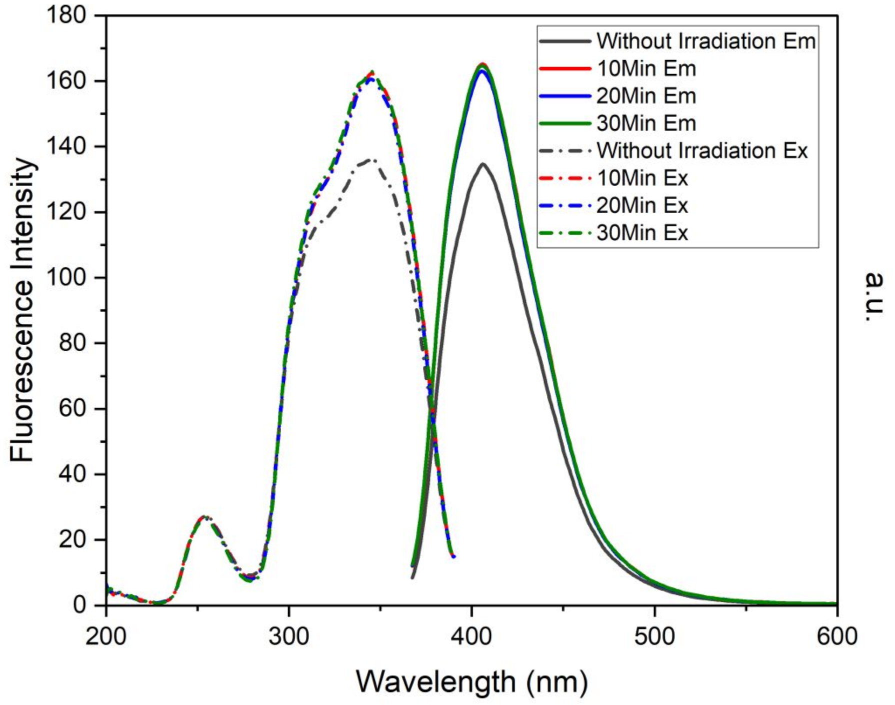

Figure S62. Fluorescence Spectra of FC2 upon irradiation in THF (10^{-5} M)

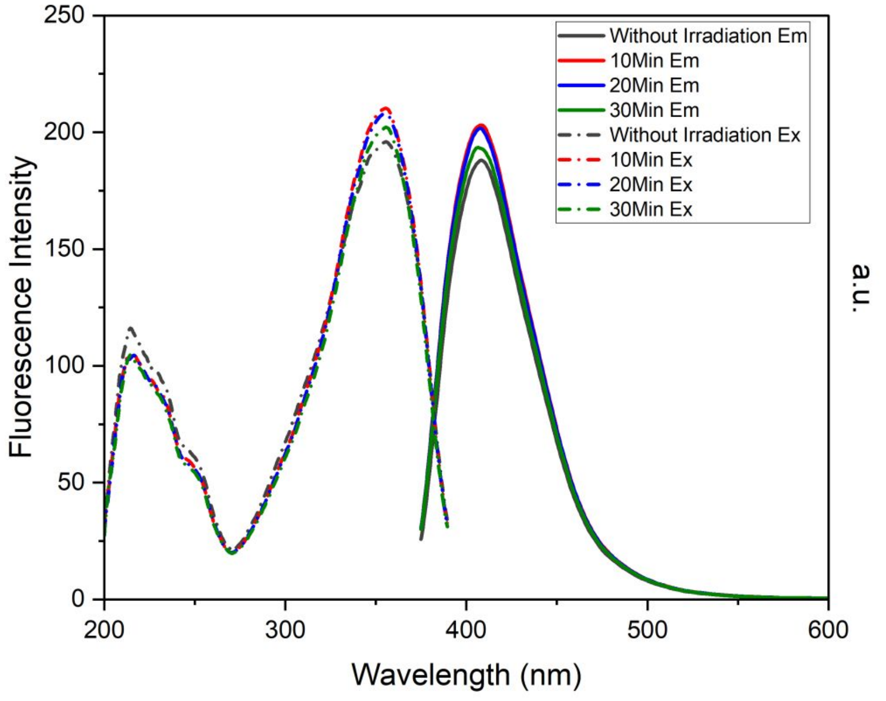

Figure S63. Fluorescence Spectra of FC2 upon irradiation in MeOH (10^{-5} M)

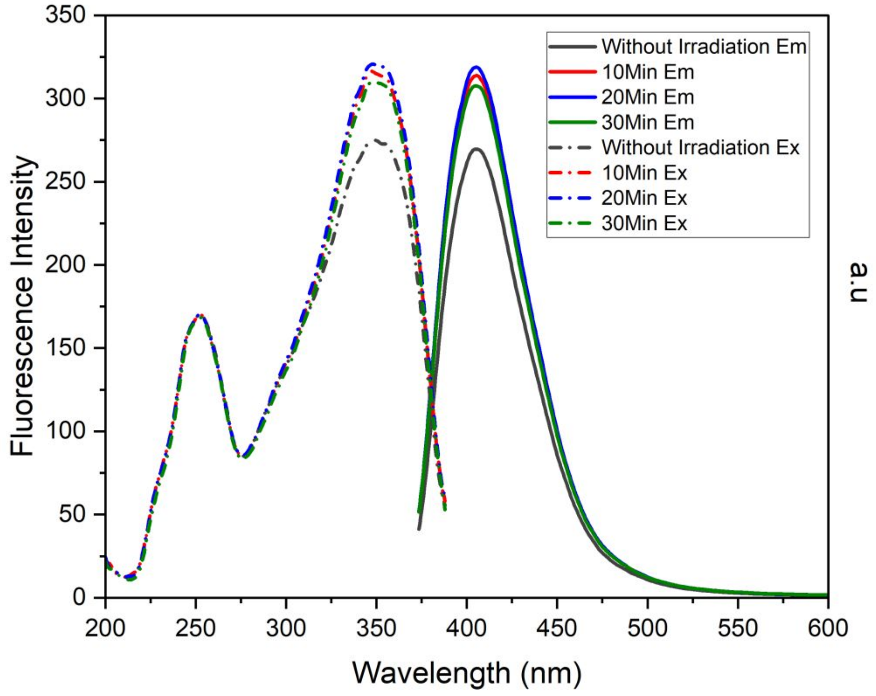

Figure S64. Fluorescence Spectra of FC3 upon irradiation in MeCN (10^{-5} M)

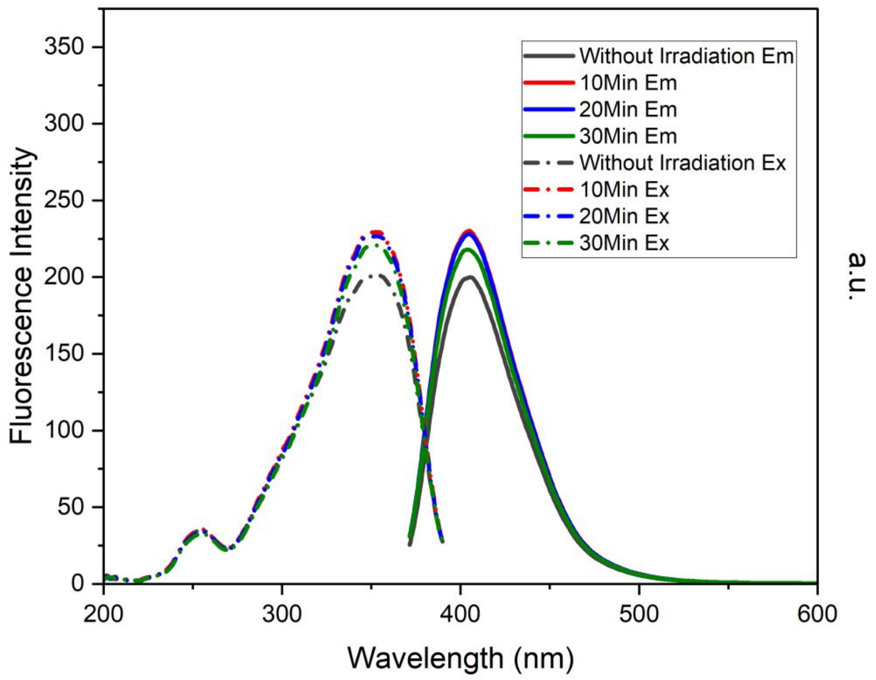

Figure S65. Fluorescence Spectra of FC3 upon irradiation in THF (10^{-5} M)

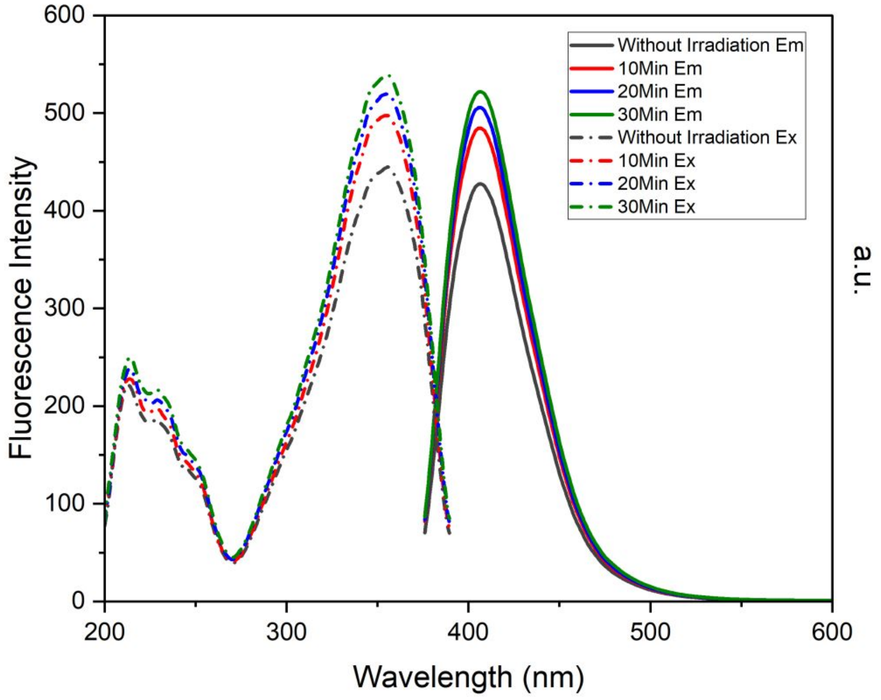

Figure S66. Fluorescence Spectra of FC3 upon irradiation in MeOH (10^{-5} M)

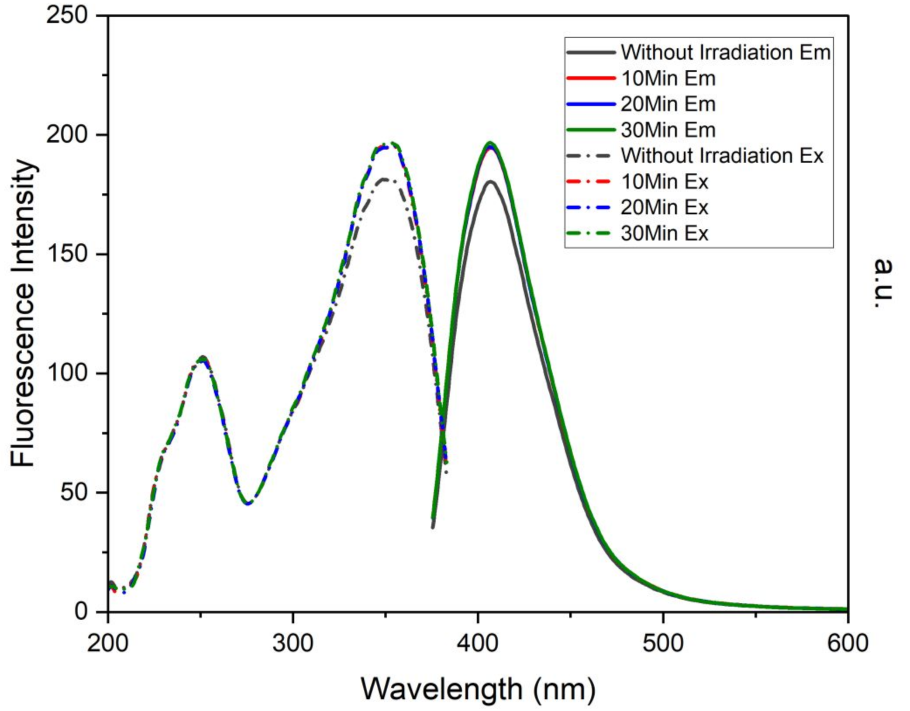

Figure S67. Fluorescence Spectra of FC4 upon irradiation in MeCN (10^{-5} M)

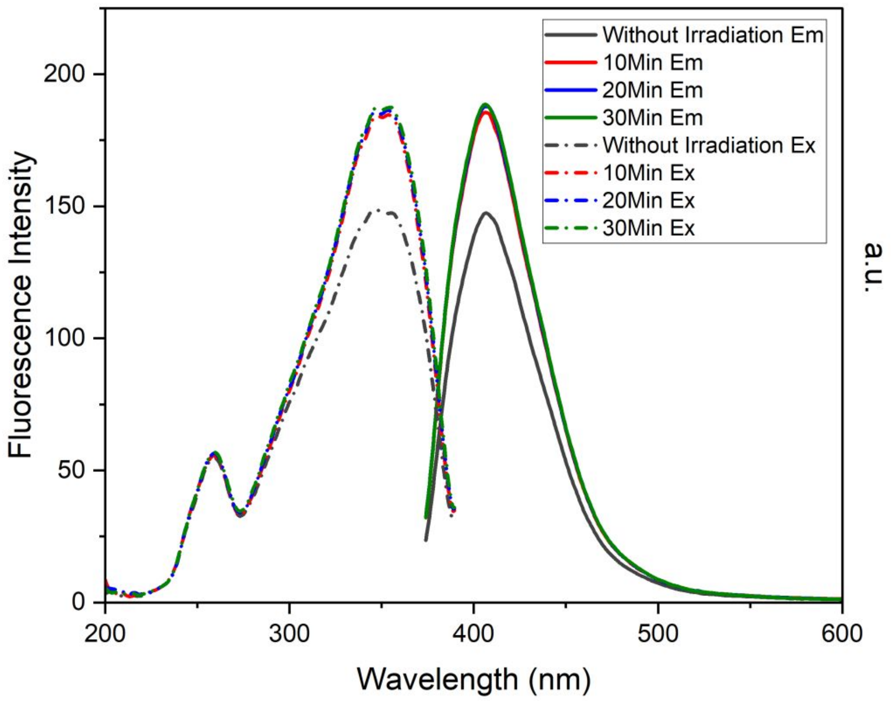

Figure S68. Fluorescence Spectra of FC4 upon irradiation in THF (10^{-5} M)

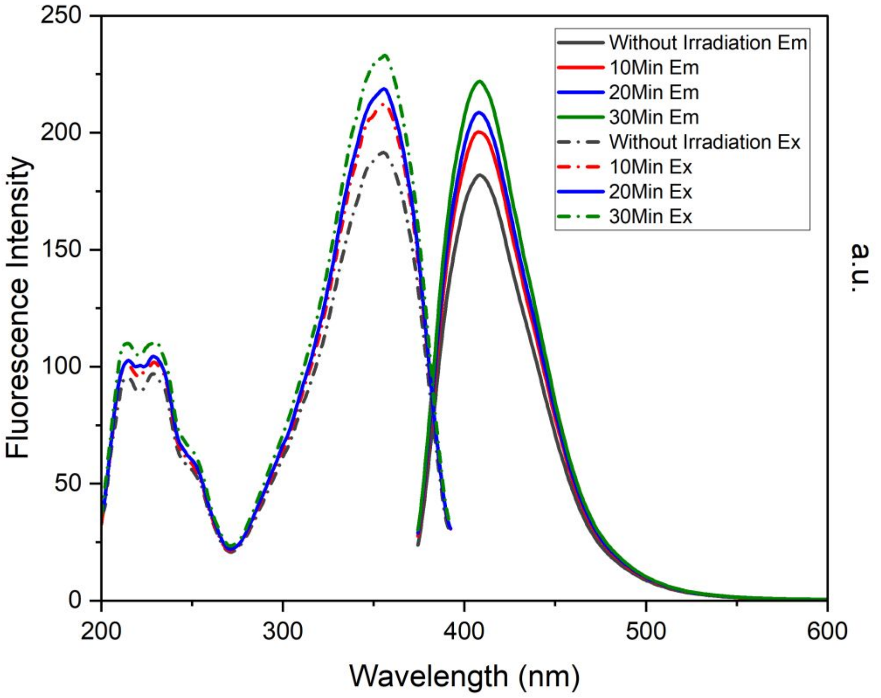

Figure S69. Fluorescence Spectra of FC4 upon irradiation in MeOH (10^{-5} M)

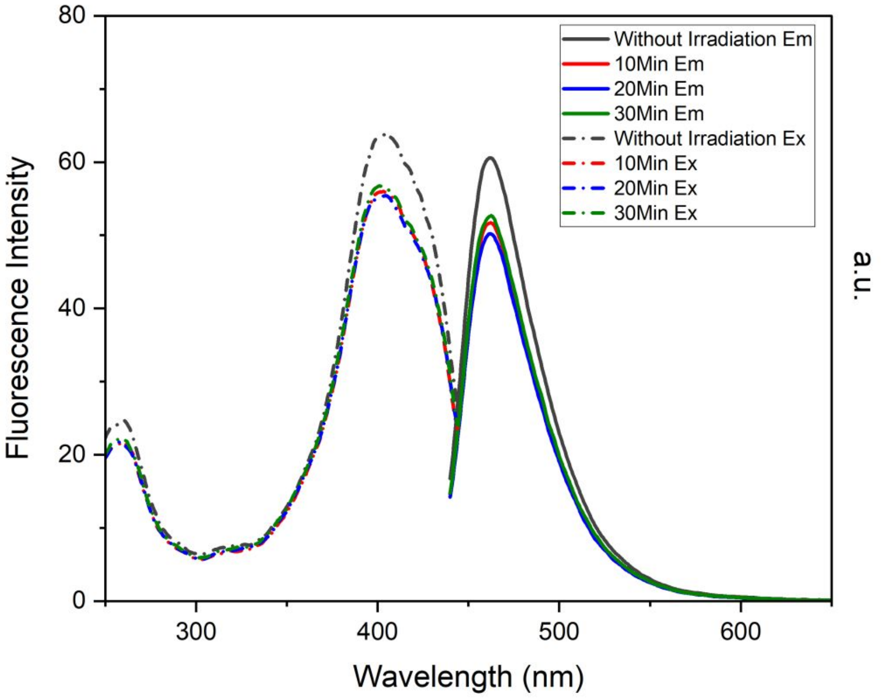

Figure S70. Fluorescence Spectra of FC5 upon irradiation in MeCN (10^{-5} M)

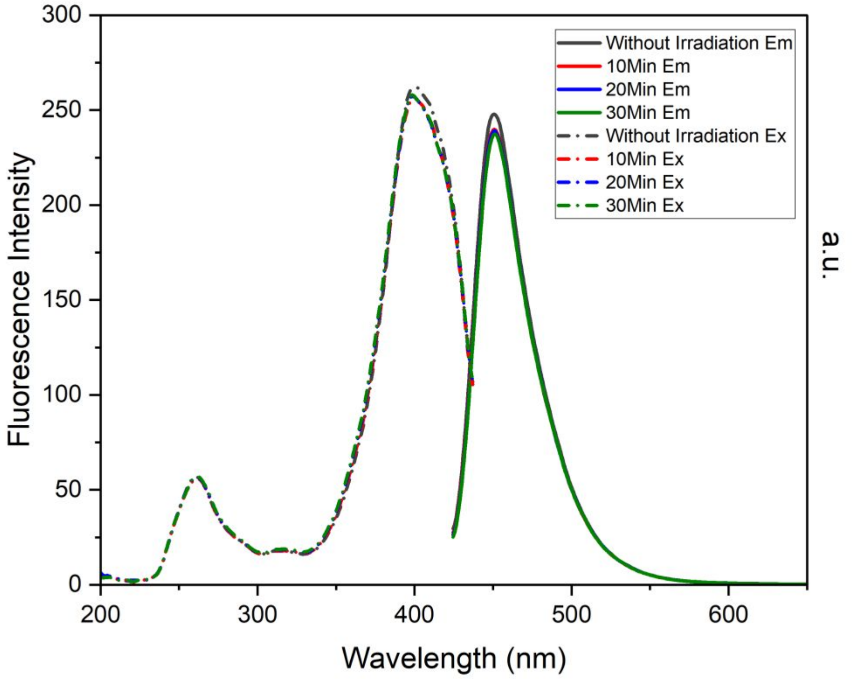

Figure S71. Fluorescence Spectra of FC5 upon irradiation in THF (10^{-5} M)

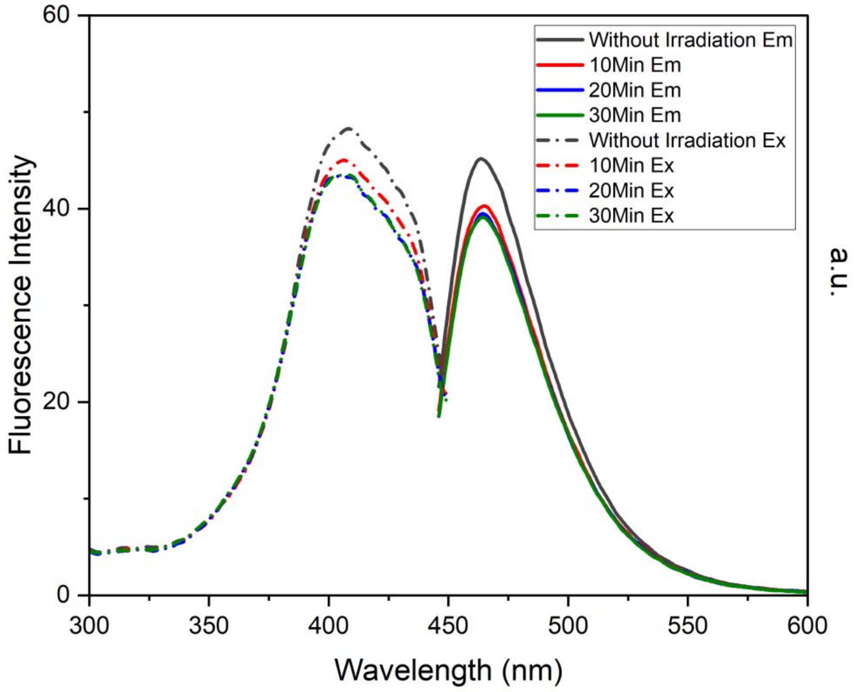

Figure S72. Fluorescence Spectra of FC5 upon irradiation in MeOH (10^{-5} M)

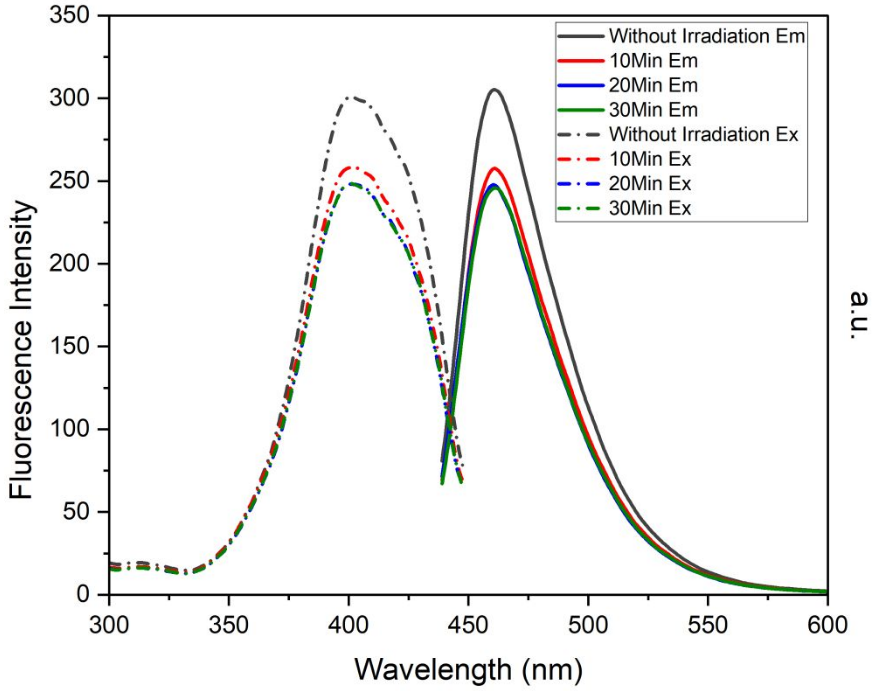

Figure S73. Fluorescence Spectra of FC6 upon irradiation in MeCN (10^{-5} M)

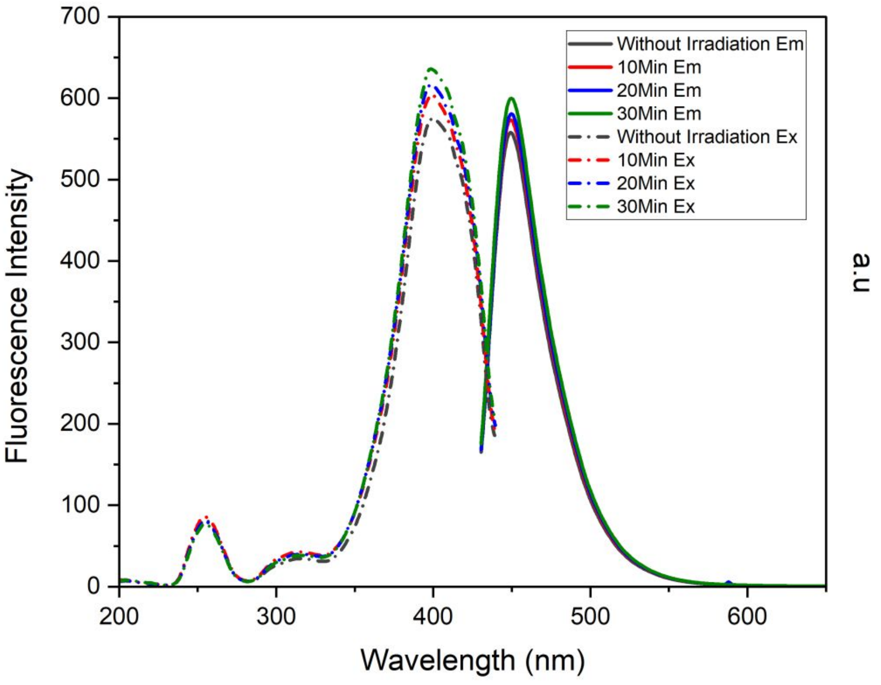

Figure S74. Fluorescence Spectra of FC6 upon irradiation in THF (10^{-5} M)

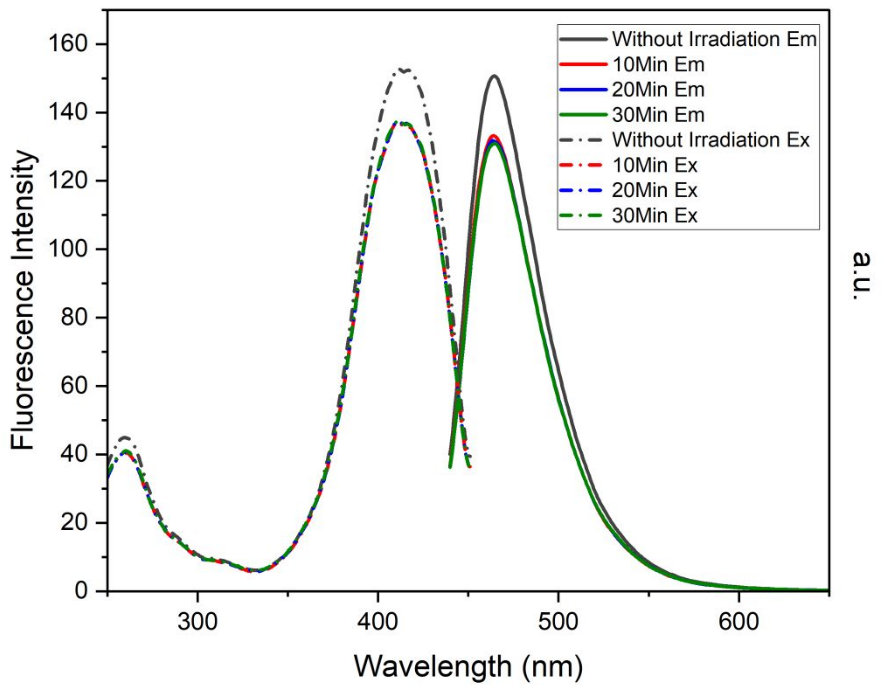

Figure S75. Fluorescence Spectra of FC6 upon irradiation in MeOH (10^{-5} M)

G. XYZ Coordinates of the Optimized Structures

For *trans*-FC1

Coordinates (Angstroms)

X	Y	Z
6.47853	-1.923249	-0.001464
7.837341	-1.533737	-0.001914
8.644774	-2.422309	-0.00274
8.076116	-0.080187	-0.000947
7.023525	0.789621	0.00013
5.668331	0.342498	0.00045
4.5508	1.196849	0.001541
3.259218	0.694832	0.001788
3.060927	-0.702872	0.00093
4.154102	-1.576541	-0.000151
5.439876	-1.051585	-0.000382
1.842593	-1.299031	0.0011
0.664373	-0.491358	0.001907
-0.526334	-1.441165	0.001621
-1.853079	-0.691122	0.00242
-2.88574	-1.671301	0.002284
-8.676871	0.785069	-0.00025
-8.314267	-0.424091	-0.000605
-10.080767	0.984433	-0.000902

-10.497329	2.322495	-0.000583
-11.855479	2.636031	-0.001165
-12.804969	1.61279	-0.002063
-12.391209	0.274339	-0.002382
-11.038427	-0.04501	-0.001809
-6.916758	-0.628665	0.000217
-6.49393	-1.968326	-0.000435
-5.144915	-2.286836	0.000238
-4.186174	-1.262194	0.001622
-4.596617	0.082018	0.002359
-5.952086	0.390694	0.001642
9.485146	0.411428	-0.001177
10.480867	-0.276249	-0.002126
9.525765	1.772327	-0.000213
10.84891	2.351843	-0.000399
10.686123	3.860325	0.000434
7.223413	1.856349	0.000783
4.711335	2.27178	0.002199
2.418036	1.376624	0.002646
3.996472	-2.648638	-0.000808
0.655372	0.153659	0.892071
0.654922	0.154771	-0.887444
-0.475326	-2.087704	-0.880994
-0.474884	-2.088782	0.883422

-1.94246	-0.050268	0.891929
-1.943076	-0.049388	-0.886383
-9.736205	3.096974	0.00012
-12.17232	3.675358	-0.000916
-13.864807	1.853062	-0.002515
-13.132535	-0.520599	-0.003078
-10.699019	-1.07476	-0.002046
-7.251133	-2.746414	-0.001514
-4.804687	-3.317318	-0.000295
-3.868607	0.885484	0.003553
-6.284772	1.4228	0.002199
11.390499	1.995861	0.881827
11.389864	1.996759	-0.883373
11.67111	4.339675	0.000303
10.140744	4.196837	-0.887419
10.141418	4.19594	0.889039

For *cis*-FC1

Coordinates (Angstroms)

X	Y	Z
-7.325824	-1.475377	0.56249
-7.541124	-0.068317	0.184444
-6.478944	0.731527	-0.124878

-5.133298	0.257788	-0.080127
-4.005509	1.041479	-0.384148
-2.72397	0.518734	-0.31561
-2.546966	-0.828	0.068784
-3.650707	-1.630769	0.377943
-4.926188	-1.086712	0.300928
-5.975587	-1.890823	0.602805
-8.937644	0.456944	0.159338
-9.924102	-0.123197	0.552587
-8.975958	1.712585	-0.365063
-1.339899	-1.438827	0.168694
-0.153636	-0.706307	-0.141836
1.020119	-1.657253	0.053407
2.353199	-0.991462	-0.266855
3.368407	-1.963797	-0.043758
4.668686	-1.625903	-0.280276
5.609885	-2.644608	-0.062073
6.954123	-2.407316	-0.299119
7.400801	-1.134965	-0.695777
6.455863	-0.126969	-0.926404
5.096629	-0.369821	-0.731104
8.792022	-1.040896	-1.021114
9.500481	-0.029727	-0.819297
9.084738	1.105938	-0.046468

9.363134	2.373571	-0.574626
9.102164	3.515435	0.180639
8.6157	3.39912	1.485175
8.386219	2.132067	2.028421
8.610091	0.985894	1.268374
-8.147319	-2.309011	0.830456
-10.283485	2.325545	-0.406884
-10.119596	3.709966	-1.005553
-6.663204	1.762464	-0.409919
-4.149916	2.077725	-0.678373
-1.874601	1.146058	-0.555756
-3.509086	-2.664217	0.671536
-0.069731	0.166471	0.521119
-0.200523	-0.344022	-1.178698
0.888485	-2.53282	-0.591201
1.031268	-2.013564	1.089047
2.522582	-0.115824	0.377333
2.380893	-0.650868	-1.312517
5.257009	-3.616948	0.266386
7.68664	-3.199284	-0.175328
6.773989	0.847486	-1.279402
4.386154	0.423699	-0.932384
9.77836	2.445042	-1.575731
9.299158	4.496786	-0.24255

8.433212	4.288896	2.081224
8.029381	2.034967	3.050418
8.433093	0.001511	1.689725
-10.690114	2.361387	0.608922
-10.948889	1.694919	-1.005037
-11.093228	4.209298	-1.056715
-9.709232	3.65315	-2.019064
-9.45032	4.325241	-0.39515

For *trans*-FC3

Coordinates (Angstroms)

X	Y	Z
-7.307835	1.738462	-0.00084
-6.030781	2.341054	-0.000694
-6.00824	3.541681	-0.001057
-4.893054	1.405716	-0.000202
-5.110683	0.056762	0.00008
-6.423433	-0.500084	-0.00009
-6.700843	-1.879864	0.000193
-8.002583	-2.353123	0.000023
-9.07352	-1.432308	-0.000443
-8.827055	-0.054252	-0.000734
-7.514554	0.397914	-0.000558

-10.380339	-1.787654	-0.00065
-10.720337	-3.170008	-0.00031
-3.513189	1.966893	0.00001
-3.214244	3.139152	0.000179
-2.579539	0.970416	0.000446
-1.211604	1.417467	0.00094
-0.331625	0.175241	0.000926
1.148597	0.537764	0.0015
1.882434	-0.684915	0.00162
7.44667	-0.930587	-0.000155
8.118078	0.138603	-0.000055
6.04547	-0.755785	0.000346
5.280396	-1.934067	0.000022
3.895386	-1.881119	0.000431
3.243711	-0.637867	0.001203
3.998764	0.548116	0.00159
5.387133	0.483978	0.001149
9.524414	-0.042948	-0.000496
10.282307	1.135877	-0.000511
11.674923	1.076825	-0.000934
12.317981	-0.162147	-0.001333
11.562741	-1.342098	-0.00131
10.173747	-1.290049	-0.000894
-4.257379	-0.613937	0.000448

-5.871543	-2.582403	0.00055
-8.186983	-3.420158	0.000245
-9.65249	0.647807	-0.001089
-11.810539	-3.204277	-0.000485
-10.339764	-3.67581	-0.896149
-10.340065	-3.675294	0.895947
-1.039178	2.043319	-0.881616
-1.039681	2.042916	0.883885
-0.552738	-0.435402	0.883595
-0.552172	-0.434982	-0.882174
1.407606	1.130963	-0.887281
1.406937	1.130738	0.890634
5.802748	-2.88595	-0.000586
3.292759	-2.783669	0.000156
3.51162	1.516546	0.002275
5.982636	1.390229	0.001438
9.754681	2.084954	-0.000192
12.256702	1.994509	-0.000949
13.40356	-0.212666	-0.001658
12.065838	-2.305692	-0.001617
9.572465	-2.192315	-0.000871

For *cis*-FC3

Coordinates (Angstroms)

X	Y	Z
6.721013	1.842625	-0.256622
5.42194	2.351759	-0.472624
5.346694	3.511437	-0.775487
4.328347	1.379714	-0.304961
4.604379	0.080908	0.016458
5.937572	-0.383693	0.216852
6.274015	-1.70724	0.556431
7.591859	-2.090308	0.743676
8.618848	-1.132802	0.591539
8.313189	0.191134	0.254379
6.985584	0.552893	0.070586
9.936608	-1.400841	0.751613
10.336081	-2.722874	1.096766
2.926235	1.850578	-0.481486
2.56631	2.99619	-0.627785
2.051612	0.802578	-0.448557
0.66394	1.160374	-0.581175
-0.136921	-0.133752	-0.551955
-1.633505	0.124173	-0.678376
-2.280406	-1.14588	-0.652938

-7.80792	-1.808852	-1.129767
-8.753978	-1.14287	-0.653007
-6.422847	-1.522933	-0.907299
-5.573089	-2.641097	-0.85141
-4.201291	-2.483514	-0.739178
-3.639177	-1.196556	-0.746471
-4.473185	-0.075846	-0.863635
-5.855832	-0.242071	-0.931746
-8.600275	-0.12733	0.349477
-7.952899	-0.370416	1.570324
-7.984531	0.5965	2.573033
-8.642538	1.811902	2.365699
-9.299335	2.045429	1.154946
-9.303454	1.069709	0.159728
3.783112	-0.619835	0.12818
5.478015	-2.438094	0.672655
7.822121	-3.115867	1.004256
9.105262	0.921522	0.138695
11.424024	-2.691576	1.168405
9.916133	-3.028234	2.062971
10.042513	-3.444167	0.324286
0.387766	1.834624	0.237457
0.5243	1.709823	-1.51846
0.184948	-0.78575	-1.371751

0.056793	-0.668336	0.384984
-1.996491	0.748127	0.151219
-1.864825	0.64497	-1.618917
-6.016062	-3.631846	-0.88877
-3.537896	-3.339768	-0.670574
-4.060715	0.925731	-0.90198
-6.490052	0.632349	-1.024371
-7.442191	-1.314473	1.730992
-7.490534	0.400216	3.521038
-8.657355	2.564505	3.149008
-9.82721	2.981323	0.992478
-9.841669	1.220085	-0.771649

For *trans*-FC5

Coordinates (Angstroms)

X	Y	Z
-6.987515	-1.899979	-0.295353
-8.068581	-0.965423	-0.215624
-7.737241	0.4027	-0.081535
-6.408229	0.805447	-0.058702
-5.341385	-0.111467	-0.152539
-5.67751	-1.477362	-0.269449
-6.164677	2.134564	0.068561

-4.869095	2.701784	0.11582
-3.76046	1.743104	0.014632
-4.019403	0.402576	-0.111026
-4.819433	3.896828	0.235283
-9.384263	-1.378878	-0.280783
-9.71972	-2.799981	-0.162313
-10.439923	-0.367538	-0.10336
-11.867039	-0.849655	-0.356051
-9.665848	-3.34402	1.271354
-2.368478	2.260349	0.051876
-2.029052	3.420268	0.122887
-1.462697	1.235986	-0.006311
-0.083691	1.639977	0.020355
0.755694	0.370478	-0.025106
2.24757	0.678651	0.003453
2.935871	-0.570188	-0.044749
4.297034	-0.576782	-0.035049
4.899484	-1.843657	-0.090552
6.281093	-1.951564	-0.086506
7.092131	-0.805834	-0.027183
6.483124	0.457782	0.028898
5.098487	0.577036	0.025212
8.484855	-1.036586	-0.029988
9.199398	0.003035	0.02391

10.597096	-0.236444	0.019428
11.402971	0.908472	0.081156
12.791922	0.791848	0.082115
13.383476	-0.471069	0.021162
12.580263	-1.617195	-0.040721
11.194625	-1.507697	-0.041948
-7.183597	-2.960306	-0.378246
-8.486736	1.175078	0.014528
-4.877505	-2.210558	-0.335987
-3.185015	-0.288034	-0.181527
-10.718723	-2.944824	-0.574844
-9.054837	-3.372554	-0.814869
-10.379176	0.066796	0.907069
-10.235599	0.448216	-0.805622
-12.537492	0.011157	-0.263739
-11.988585	-1.257312	-1.365185
-12.198676	-1.600175	0.368314
-9.919372	-4.410464	1.283148
-8.667993	-3.224509	1.705414
-10.378447	-2.819829	1.917912
0.11663	2.295493	-0.834766
0.102801	2.223468	0.928848
0.504466	-0.268849	0.828737
0.52329	-0.194654	-0.934875

2.537568	1.299814	-0.856155
2.519407	1.221573	0.920061
4.261497	-2.720375	-0.136436
6.765622	-2.922318	-0.129391
7.113913	1.338628	0.074995
4.649727	1.562868	0.069208
10.914826	1.877344	0.127622
13.411034	1.683504	0.130152
14.466057	-0.566357	0.021593
13.043152	-2.599607	-0.088319
10.556658	-2.383086	-0.089633

For *cis*-FC5

Coordinates (Angstroms)

X	Y	Z
-6.653686	-1.686392	0.670389
-7.674046	-0.693793	0.522158
-7.26394	0.611253	0.165352
-5.916779	0.904454	-0.003166
-4.908543	-0.067809	0.156007
-5.324316	-1.372955	0.496872
-5.597347	2.178982	-0.34296
-4.274513	2.634058	-0.556188

-3.226561	1.618431	-0.385915
-3.562457	0.332989	-0.047307
-4.155476	3.79241	-0.854421
-9.006012	-0.993499	0.733344
-9.445821	-2.390401	0.793596
-10.00246	0.063584	0.492575
-11.425895	-0.25762	0.944232
-9.518692	-3.086513	-0.571768
-1.810486	2.019292	-0.587056
-1.401838	3.131106	-0.837075
-0.972204	0.945951	-0.451601
0.424243	1.239439	-0.623195
1.180653	-0.072983	-0.47027
2.683257	0.118453	-0.634364
3.289335	-1.16334	-0.479897
4.643628	-1.269635	-0.581748
5.163752	-2.566826	-0.442313
6.527789	-2.782309	-0.551931
7.412931	-1.706209	-0.736908
6.88778	-0.416597	-0.892017
5.512465	-0.196974	-0.827325
8.784168	-2.061418	-0.943305
9.75886	-1.380159	-0.553642
9.654686	-0.25776	0.334708

10.39193	0.887911	0.007167
10.437472	1.963832	0.892007
9.79592	1.883119	2.130454
9.102915	0.719913	2.476476
9.022172	-0.346975	1.583967
-6.91173	-2.705274	0.925615
-7.965669	1.415881	-0.000535
-4.571486	-2.147439	0.621144
-2.77232	-0.401948	0.069765
-8.783133	-2.939237	1.46831
-10.424448	-2.41304	1.273978
-9.674092	0.952521	1.041491
-10.013427	0.339842	-0.573963
-12.038824	0.638375	0.800953
-11.887623	-1.06333	0.364955
-11.465367	-0.52106	2.00647
-9.847302	-4.125474	-0.452577
-10.230109	-2.580555	-1.23399
-8.542448	-3.090788	-1.066975
0.578545	1.689721	-1.610119
0.731066	1.980069	0.124222
0.979275	-0.503568	0.517226
0.826924	-0.792208	-1.217592
2.921662	0.528666	-1.626403

3.076316	0.813268	0.121901
4.473637	-3.387618	-0.274936
6.937399	-3.785976	-0.487796
7.54889	0.420985	-1.084137
5.132209	0.808145	-0.968297
10.917178	0.91985	-0.943034
10.991863	2.858741	0.62226
9.84907	2.714078	2.828248
8.620035	0.642871	3.447131
8.484656	-1.250704	1.852586

H. The Wavelength Profile of the Irradiation Source

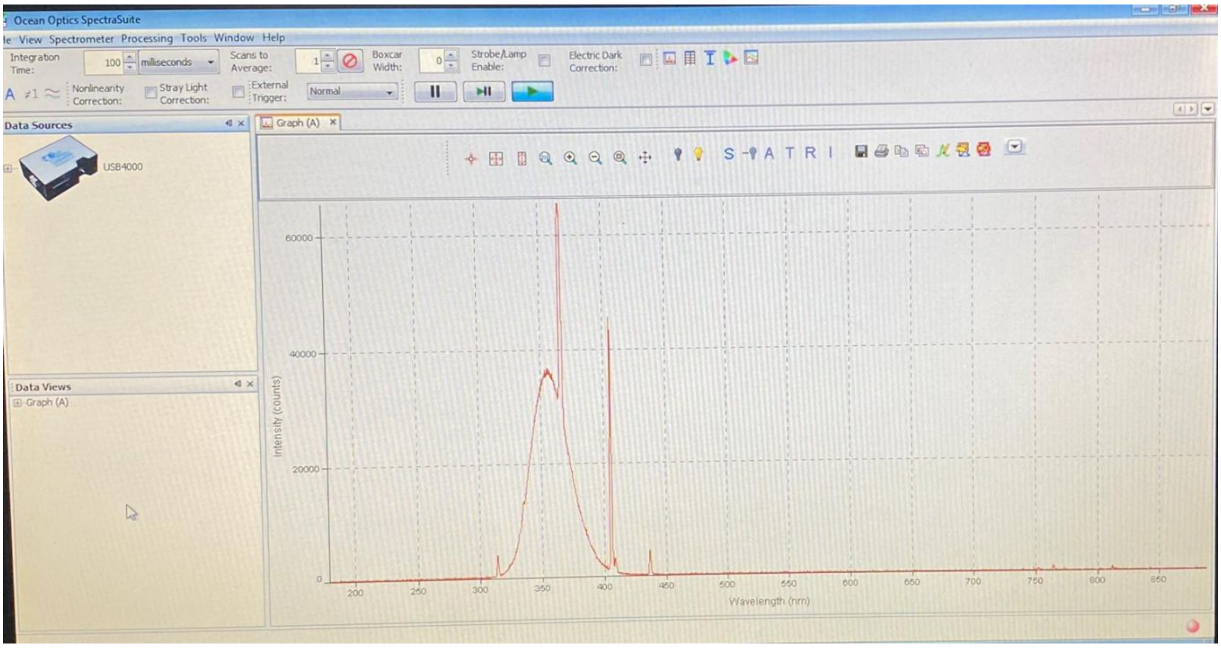

Figure S76. The Wavelength Profile of the Irradiation Source

I. ^{1}H NMR of the FC3 Before and After Irradiation

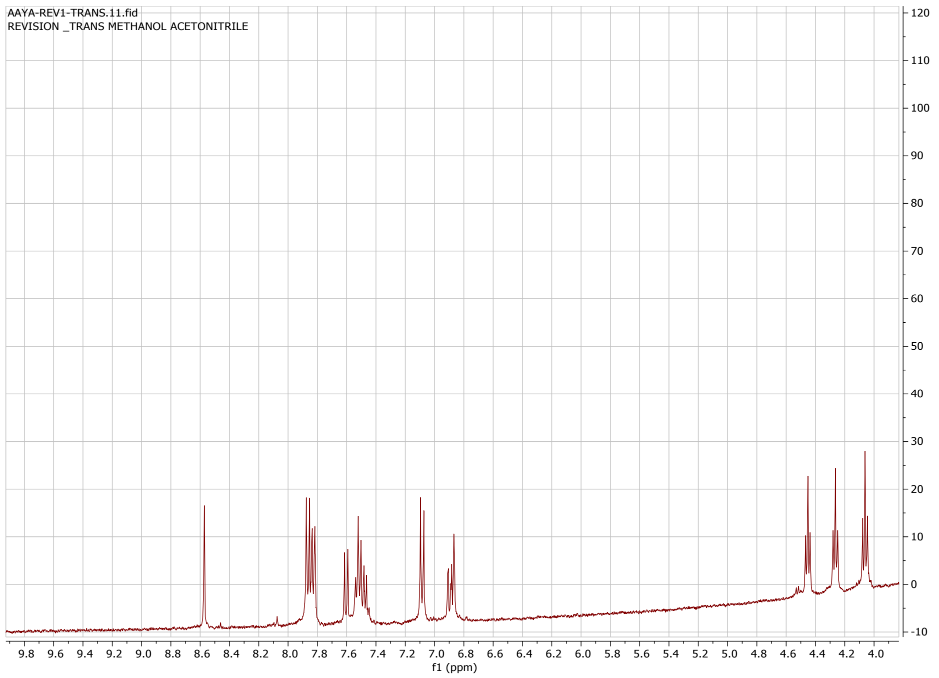

Figure S77. ^{1}H NMR of FC3 Before Irradiation in Acetonitrile and CD3OD (0.01g in 20 ml, ca. 10-3 M)

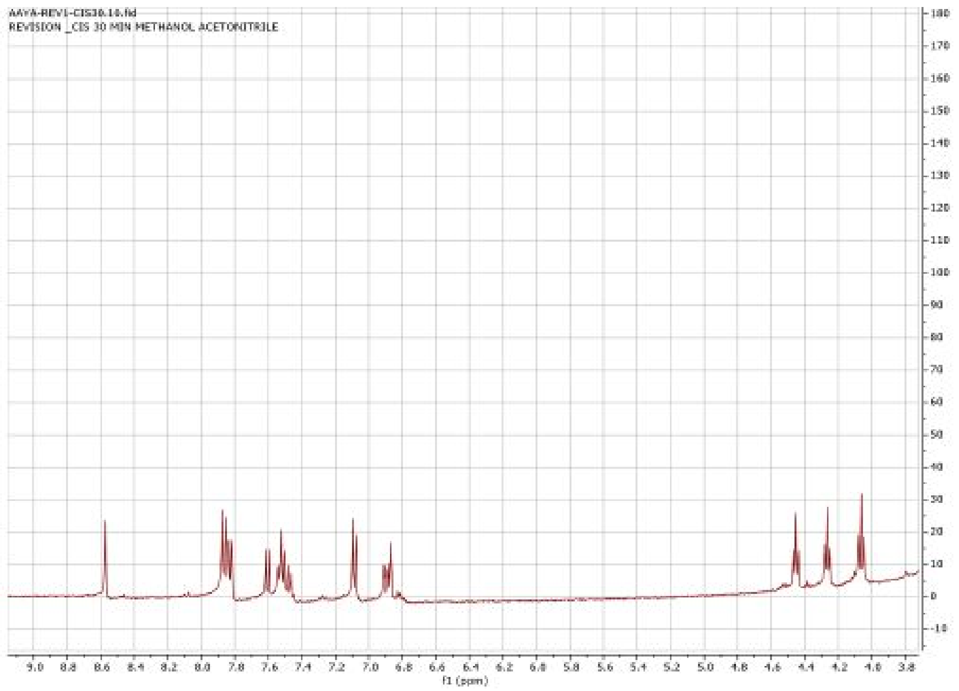

Figure S78. ^{1}H NMR of FC3 after 30 min irradiation in acetonitrile and CD3OD (0.01g in 20 ml)

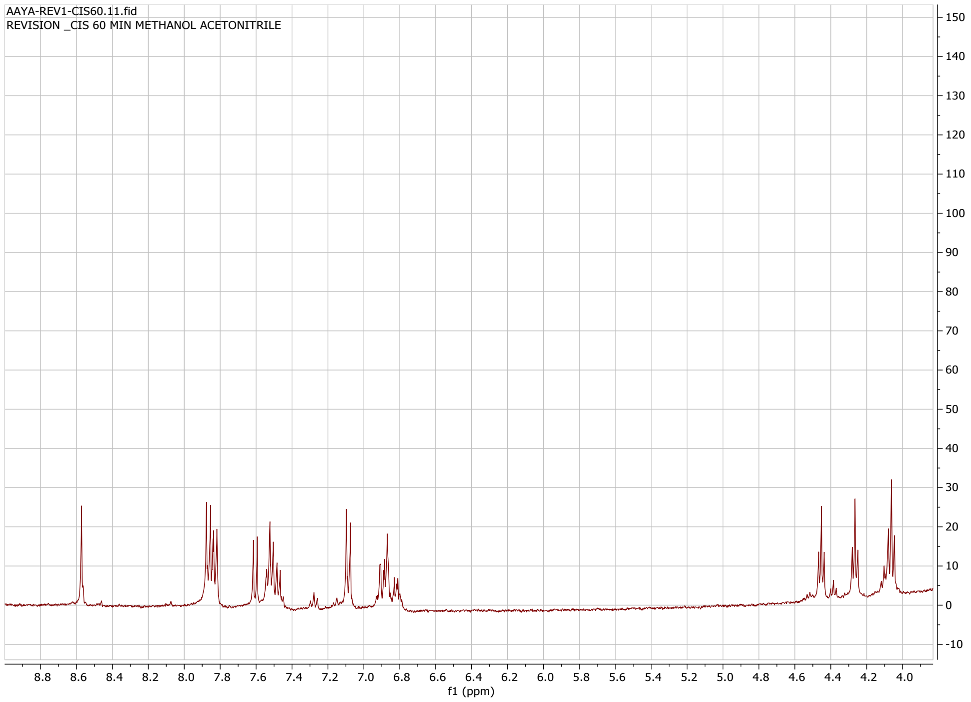

Figure S79. **^{1}H NMR of FC3 after 60 min irradiation in acetonitrile and CD3OD (0.01g in 20 ml, *ca.* 10^{-3} M)**

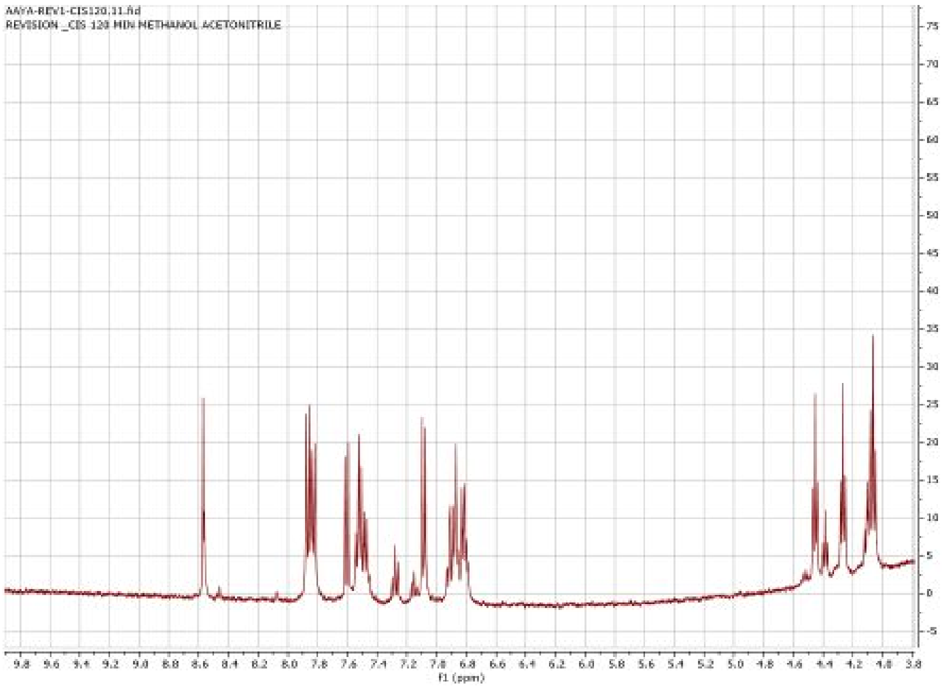

Figure S80. **^{1}H NMR of FC3 after 120 min irradiation in acetonitrile and CD3OD (0.01g in 20 ml , *ca.* 10^{-3} M)**

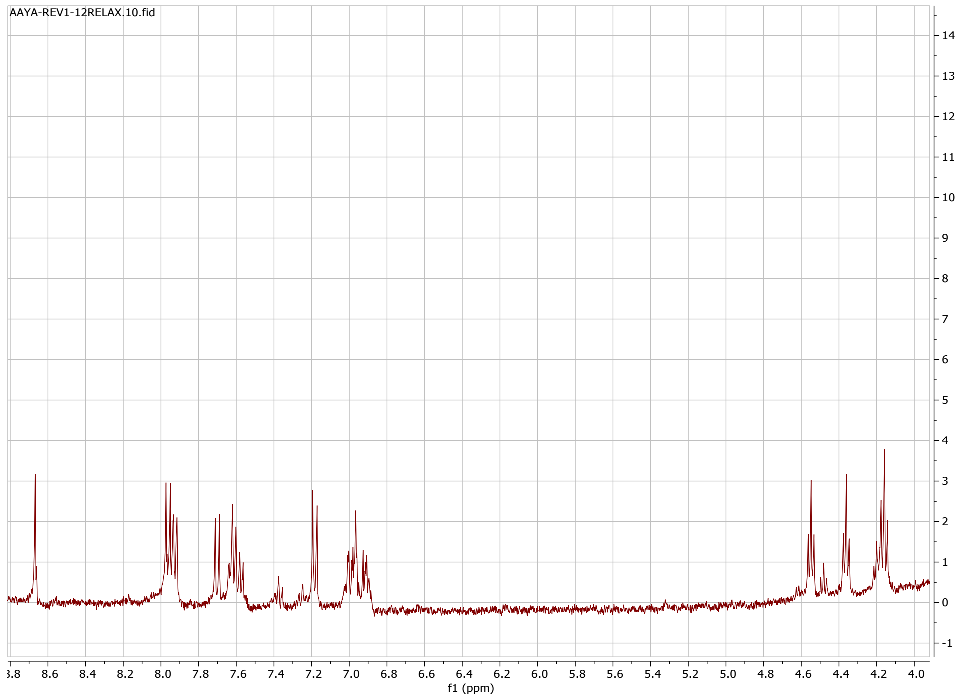

Figure S81. ^{1}H NMR of FC3 after irradiation, waited in the dark at room temperature for 12 h (0.01g in 20 ml)

J. Concentration-Dependent Fluorescence Studies of FC3

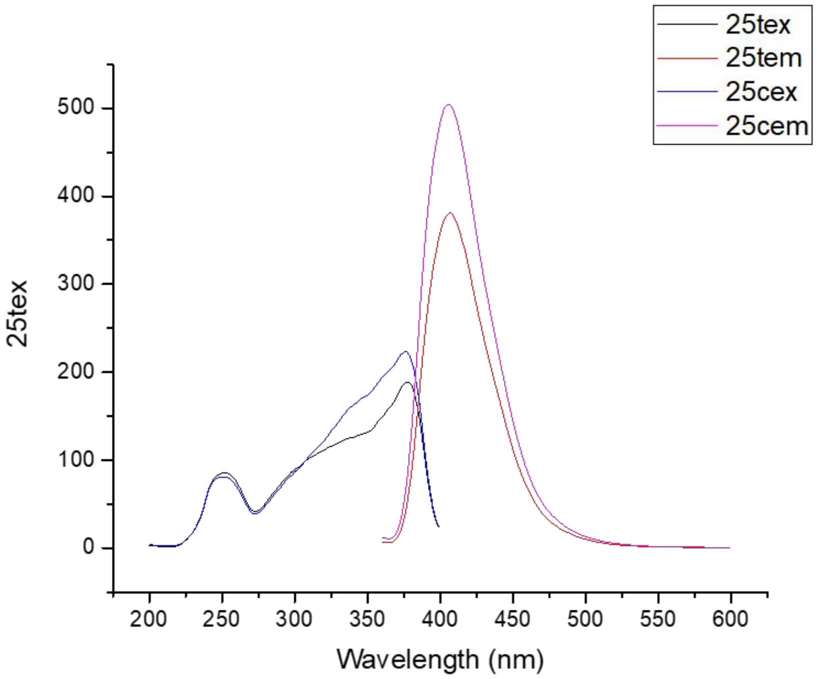

Figure S82. Excitation and Emission Spectra of FC3 (2.5x10-5 M) (t: trans, c: cis, ex: excitation, em: emission)

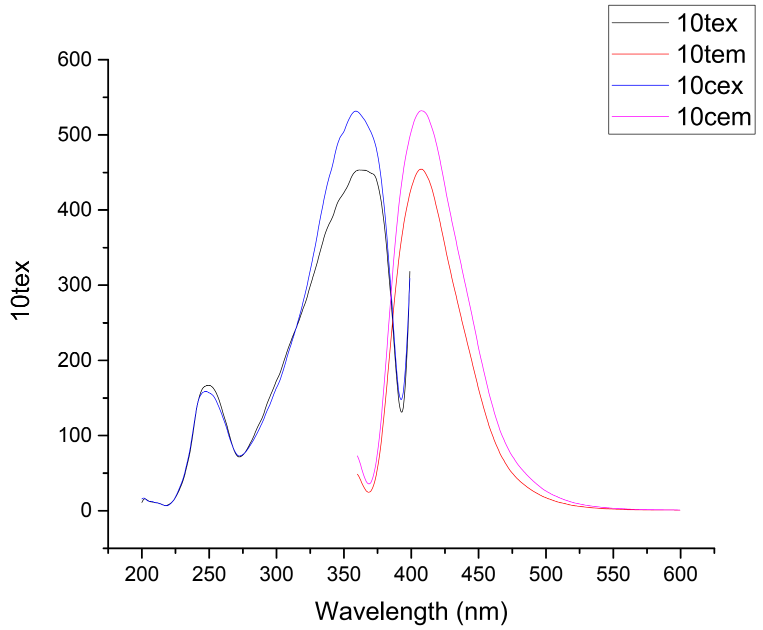

Figure S83. Excitation and Emission Spectra of FC3 (1x10-5 M) (t: trans, c: cis, ex: excitation, em: emission)

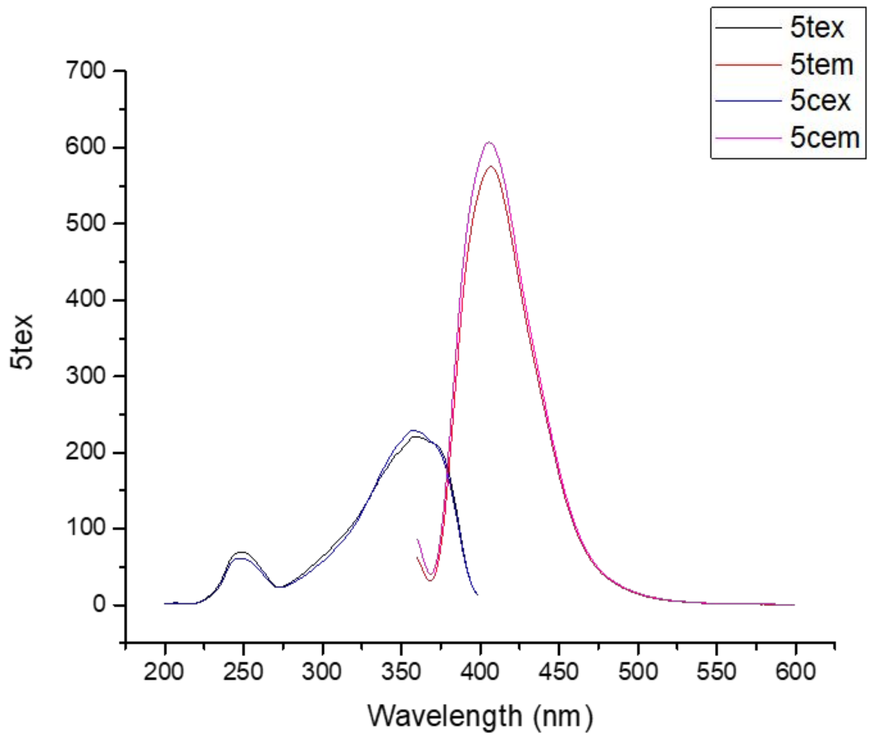

Figure S84. Excitation and Emission Spectra of FC3 (0.5x10-5 M) (t: trans, c: cis, ex: excitation, em: emission)
